# GLUL mediates FOXO3 O-GlcNAcylation to regulate the osteogenic differentiation of BMSCs and senile osteoporosis

**DOI:** 10.1038/s41418-025-01543-2

**Published:** 2025-07-11

**Authors:** Lu Zhang, Bao Qi, Yanpeng Li, Xiao Liang, Zifang Zhang, Tao Yang, Shu Jia, Xu Gao, Shang Chen, Guangjun Jiao, Yangyang Li, Hongming Zhou, Yunzhen Chen, Yanming Li, Bin Zhang, Gang Li, Chunyang Meng

**Affiliations:** 1 Department of Spine Surgery, Affiliated Hospital of Jining Medical University, Jining, 272029 China; 2 Postdoctoral Mobile Station of Shandong University of Traditional Chinese Medicine, Jinan, 250355 China; 3 Key laboratory of cell and biomedical Technology of Shandong Province, Affiliated Hospital of Jining Medical University, Jining, 272000 China; 4 Clinical Research Team of Spine & Spinal Cord Diseases, Medical Research Center, Affiliated Hospital of Jining Medical University, Jining, 272000 China; 5 Department of Spine Center, Xinhua Hospital Affiliated to Shanghai Jiaotong University School of Medicine, Shanghai, 200092 China; 6 Qingdao Medical College of Qingdao University, Qingdao, 266003 China; 7 Department of Spine Surgery, Qilu Hospital of Shandong University, Jinan, 250012 China; 8 Department of Pathology, The First Affiliated Hospital of Wenzhou Medical University, Wenzhou, 325000 China; 9 Department of Orthopaedics, Linyi Central Hospital, Linyi, 276401 China; 10 Department of Spine Surgery, Shandong Provincial Hospital Affiliated to Shandong First Medical University, Jinan, 250021 China; 11 Department of Laboratory Medicine, Affiliated Hospital of Jining Medical University, Jining Medical University, Jining, 272000 China; 12 Department of Orthopaedics, Shandong University of Traditional Chinese Medicine, Jinan, 250014 China

**Keywords:** Diseases, Metabolomics, Ageing

## Abstract

The abnormal osteogenic differentiation of bone marrow mesenchymal stem cells (BMSCs) is an important cause of senile osteoporosis (SOP). Glutamine synthetase (GLUL) is a key enzyme in glutamine biosynthesis; however, its functional role in SOP remains unclear. Here, we found that GLUL expression was downregulated in the BMSCs of SOP patients. Mice with BMSC-specific *Glul*-knockout (KO) exhibited dysplasia of the skull and phalanges and osteoporosis due to disordered osteogenic differentiation. Mechanistically, GLUL competitively bound to the Tripartite Motif Containing 25 (TRIM25) SPRY subunit, reduced the ubiquitin-mediated degradation of UDP-N-acetylglucosamine pyrophosphorylase 1 (UAP1) and increased the synthesis of uridine 5-diphosphate N-acetylglucosamine (UDP-GlcNAc), thereby regulating the O-linked β-N-acetylglucosamine modification (O-GlcNAcylation) of serine 296 residues and increasing Forkhead Box O3 (FOXO3) stability to reduce oxidative stress. Moreover, blocking the O-GlcNAcylation of FOXO3 at Ser296 inhibited osteogenic differentiation. Finally, GLUL supplementation specifically in BMSCs slowed bone loss in SOP model mice. Overall, our study suggests that GLUL plays an important role in regulating osteogenic differentiation and bone development, which may have implications for SOP treatment.

**Figure:**
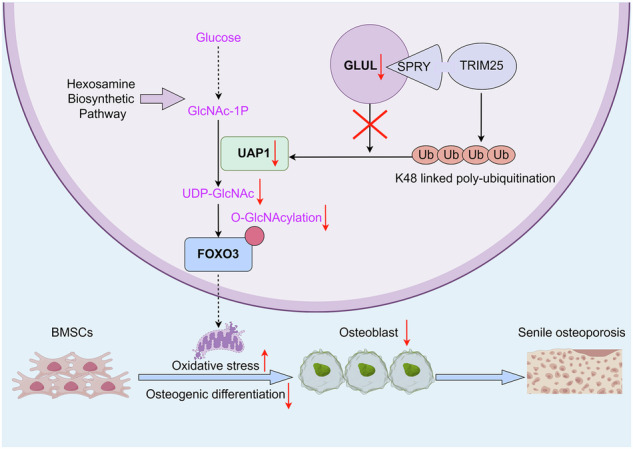
Schematic illustration of the molecular mechanism by which GLUL mediates FOXO3 O-GlcNAcylation to regulate the osteogenic differentiation of BMSCs and senile osteoporosis. The graphical abstract was created by figdraw2.0.

Schematic illustration of the molecular mechanism by which GLUL mediates FOXO3 O-GlcNAcylation to regulate the osteogenic differentiation of BMSCs and senile osteoporosis. The graphical abstract was created by figdraw2.0.

## Introduction

Population aging is a global trend, and SOP-related health problems will become increasingly prominent. SOP is characterized by decreased bone mass, destruction of the bone microstructure, and susceptibility to fracture [1, 2]. SOP generally refers to osteoporosis in individuals over the age of 70, and SOP-induced fractures increase disability and mortality in elderly individuals [3, 4]. Changes in the quantity and function of BMSCs, especially decreases in their osteogenic differentiation, are among the key causes of SOP [5, 6]. Thus, understanding how BMSC osteogenic differentiation is regulated will provide insights for the development of novel therapeutic strategies for SOP.

GLUL is responsible for converting glutamate and ammonia into glutamine, and it plays important roles in various physiological and pathological processes [7–9]. Studies have shown that when glutamine is present at normal physiological levels, the glutamine synthesis activity of GLUL in vascular endothelial cells is almost negligible. However, the absence of GLUL significantly affects the growth of vascular buds during vascular development [10]. In an Alzheimer’s disease mouse model, GLUL expression decreases with age, which may lead to defects in glutamate transmission and affect cognitive function [11]. Nevertheless, the role of GLUL in SOP has not been characterized.

O-linked β-N-acetylglucosamine modification (O-GlcNAcylation) is a posttranslational modification of proteins in which N-acetylglucosamine is added to serine or threonine residues [12]. The hexosamine biosynthesis pathway (HBP) provides the UDP-GlcNAc that is necessary for this modification [13, 14]. UAP1 is one of the key enzymes that regulates the HBP pathway to generate substrates [13, 15]. O-GlcNAcylation is closely associated with a variety of physiological processes, including signal transduction, transcriptional regulation, protein stability, and cell survival [16–18]. Moreover, this modification is closely related to osteoblast metabolism and bone formation [19, 20]. Additionally, the O-GlcNAcylation of key transcription factors modulates the osteogenic/lipogenic differentiation of BMSCs [21]. Although studies have shown that GLUL is involved in protein O-GlcNAcylation [22], its regulatory role in this process has not been fully characterized.

Protein ubiquitination is a dynamic posttranslational modification that participates in almost all aspects of eukaryotic biology, including osteogenic differentiation [23–25]. E3 ubiquitin ligases determine the precise substrate specificity of ubiquitination [26, 27]. However, the detailed mechanism by which ubiquitination regulates UDP-GlcNAc generation is still poorly understood.

In this study, we investigated the role of GLUL in the progression of SOP. We revealed the molecular mechanism by which GLUL increases UDP-GlcNAc biosynthesis and induces protein O-GlcNAcylation, and we described the importance of FOXO3 O-GlcNAcylation for BMSC osteogenic differentiation. Importantly, our study reveals a new link between GLUL- and HBP-mediated O-GlcNAcylation modification, and we propose a novel strategy for treating SOP.

## Results

### GLUL plays an important role in the osteogenic differentiation of BMSCs and the progression of SOP

**Fig. 1:**
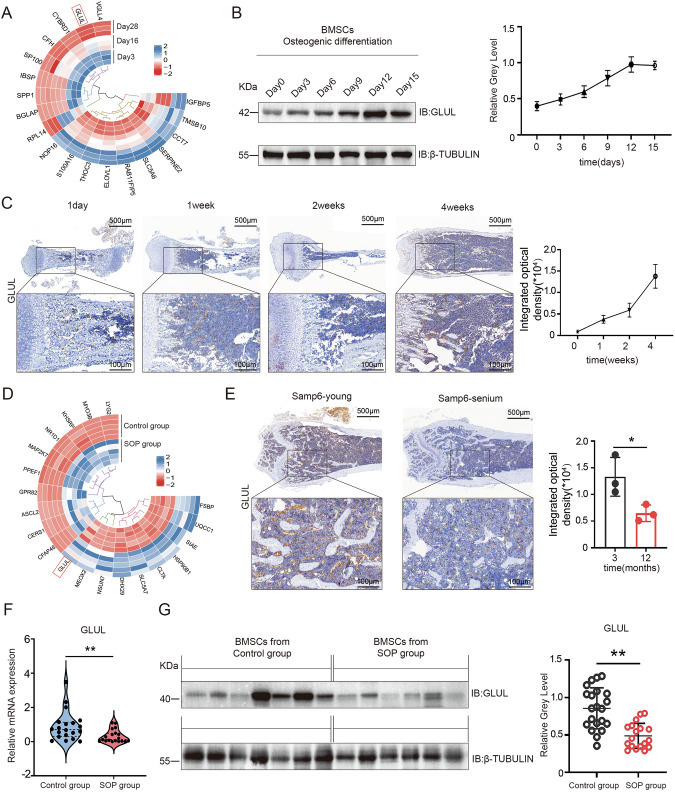
GLUL plays an important role in the osteogenic differentiation of BMSCs and the progression of SOP. **A** Heatmap of the metabolic and transcriptional changes that occur during the osteogenic differentiation of human mesenchymal stem cells according to the GSE191136 dataset. **B** Western blotting analysis of GLUL expression during the osteogenic differentiation of BMSCs. **C** Immunohistochemical staining analysis of GLUL expression in mouse femur sections at different times after birth. **D** Heatmap of the effects of SOP on human mesenchymal stem cells according to the GSE35956 dataset. **E** Immunohistochemical staining analysis of GLUL expression in femur sections from young and old samp6 mice (*n *= 3). **F** Quantification of GLUL expression in bone samples from patients with SOP. **G** Western blotting analysis of GLUL expression in BMSCs from patients in the SOP group (*n * = 6) and the control group (*n *= 7). ** *P *< 0.01 vs. other groups; *P < 0.05 vs. other groups.

We first used the Gene Expression Omnibus database to examine the expression of GLUL during the osteogenic differentiation of stem cells. Compared with that in the control group, the expression of GLUL was upregulated during the osteogenic differentiation of BMSCs (Figs. 1A and S1A). Then, we induced the osteogenic differentiation of BMSCs from C57BL/6 mice and measured the expression of GLUL at different time points. We found that the expression of GLUL increased with time (Fig. 1B). Then, immunohistochemical staining was performed on femoral sections from the mice on the first day and in the first week, second week, and fourth week after birth. The results revealed that GLUL expression gradually increased during bone development (Fig. 1C).

Owing to the close correlation between the disordered osteogenic differentiation of BMSCs and the occurrence of osteoporosis, we analysed data related to animal models and human osteoporosis; we found that GLUL expression differed in the BMSCs from animals and humans with different types of osteoporosis. That is, GLUL was upregulated in a postmenopausal osteoporosis animal model (Fig. S1B). However, GLUL expression was downregulated in the BMSCs of elderly patients with osteoporosis (SOP) (Fig. 1D). To confirm this conclusion, we measured the expression of GLUL in the femoral tissues of SAM-P6 mice at 4 and 12 months of age, and we found that GLUL expression was downregulated in aged SAM-P6 mice (Figs. 1E and S1C). GLUL expression also tended to decrease in bone and BMSCs from SOP patients (Figs. 1F, G and S1D). These data suggest that GLUL may play an important role in the osteogenic differentiation of aged BMSCs and the pathogenesis of SOP.

### Mesenchymal stem cell-specific *Glul* deficiency leads to abnormal bone formation

**Fig. 2:**
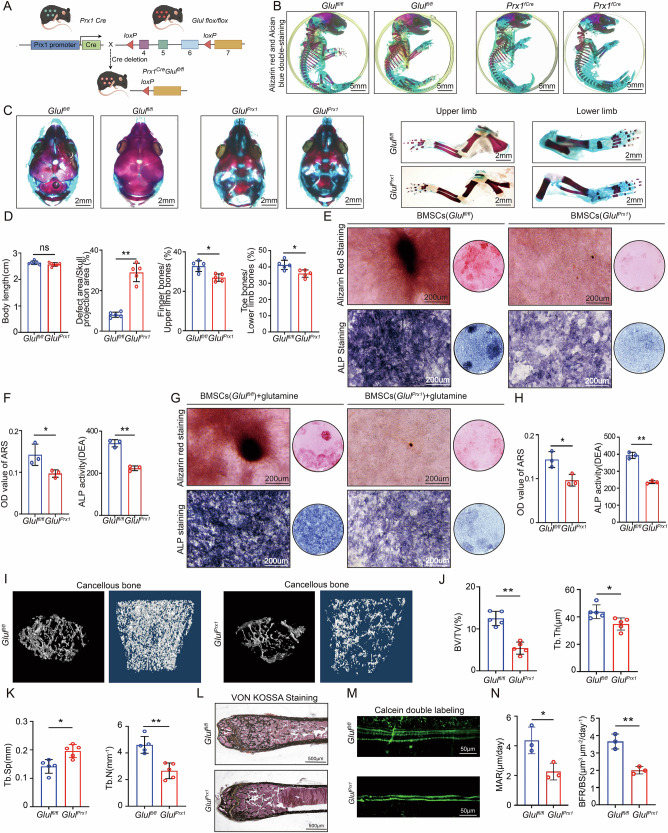
Mesenchymal stem cell-specific *Glul* deficiency leads to abnormal bone formation. **A** Construction strategies for BMSC-specific *Glul-*KO (*Glul*^*Prx1*^) mice. **B** Representative images of Alizarin Red-Alcian Blue double staining of tissues from *Glul*^*Prx1*^ and *Glul*^*fl/fl*^ mice(*n *= 5). **C** Representative images of Alizarin Red-Alcian Blue double staining of the skull, upper limb, and lower limb from *Glul*^*Prx1*^ and *Glul*^*fl/fl*^ mice (*n *= 5). **D** Quantitative analysis of Alizarin Red-Alcian Blue double staining. **E** Representative images of ARS and ALP staining of BMSCs from *Glul*^*Prx1*^ and *Glul*^*fl/fl*^ mice. **F** Quantitative analysis of ARS and ALP staining. **G** Representative images of ARS and ALP staining of BMSCs from *Glul*^*Prx1*^ and *Glul*^*fl/fl*^ mice after treatment with glutamine. **H** Quantitative analysis of ARS and ALP staining. **I** Representative micro-CT images of trabecular bone from the femoral metaphysis of *Glul*^*Prx1*^ and *Glul*^*fl/fl*^ mice (*n *= 5). **J** Quantitative analysis of cancellous bone volume (BV/TV, %), trabecular thickness (Tb.Th). **K** Quantitative analysis of trabecular number (Tb.N), and trabecular separation (Tb.Sp). **L** Representative images of VON KOSSA staining (*n *= 3). **M** Calcein double-label staining image. **N** Quantitative analysis of the MAR and BFR/BS in *Glul*^*Prx1*^ and *Glul*^*fl/fl*^ mice (*n *= 3). **P < 0.01 vs. other groups; *P < 0.05 vs. other groups.

To explore the role of GLUL in the commitment of mesenchymal stem cells to the osteoblast lineage, we generated *Prx1*Cre and *Glul* flox/flox mice (referred to as *Glul*^*Prx1*^ mice) (Figs. 2A and S2A), in which limb and craniofacial mesenchymal cells were the sites of *Prx1Cre* activity. We used Western blotting to confirm that *Glul* was knocked out in limb BMSCs (Fig. S2B). The survival and fertility of the *Glul*^*Prx1*^ mice were normal after birth. However, compared with *Glul*^*fl/fl*^ mice, *Glul*^*Prx1*^ mice presented delayed bone development, especially in limb bones (Fig. 2B–D), and these effects were independent of the sex of the mice. To evaluate the role of GLUL in BMSC osteogenic differentiation, BMSCs from *Glul*^*fl/fl*^ and *Glul*^*Prx1*^ mice were cultured with osteogenic medium to induce differentiation, and sufficient glutamine was added to eliminate the effects of insufficient GLUL enzyme activity. The results indicate that *Glul* knockout (KO) led to a significant decrease in cellular ALP activity and mineralization (Fig. 2E–H). The results of haematoxylin‒eosin staining indicated a decrease in trabecular bone in the femurs of *Glul*^*Prx1*^ mice (Fig. S2D). Moreover, the Western blotting and immunohistochemical staining results suggested that *Glul-*KO reduced the expression of osteogenic marker genes (RUNX2, CoL1A1, OSX, and SPP1) (Fig. S2C and S2E).

To further determine the function of GLUL in the skeletal system, we compared the static bone parameters of femurs from *Glul*^*fl/fl*^ and *Glul*^*Prx1*^ mice with a microquantitative computed tomography (micro-CT) system. We found that the bone mass per unit volume (BV/TV), trabecular number (Tb.N) and trabecular thickness (Tb.Th) of 16-week-old *Glul*^*Prx1*^ mice decreased (Fig. 2I–K) and that the trabecular separation (Tb.Sp) increased. Von Kossa staining revealed a decrease in bone mineral deposition in 16-week-old *Glul*^*Prx1*^ mice (Fig. 2L), and the calcium double-labelling experiment suggested that the bone formation rate of *Glul*^*Prx1*^ mice decreased (Fig. 2M), the bone mineral apposition rate (MAR) decreased, and the same to bone formation rate/bone surface (BFR/BS) (Fig. 2N). Overall, there are obstacles to bone development and BMSC osteogenic differentiation in conditional *Glul-*KO mice.

### GLUL regulates HBP metabolism and affects UDP-GlcNAc generation

**Fig. 3:**
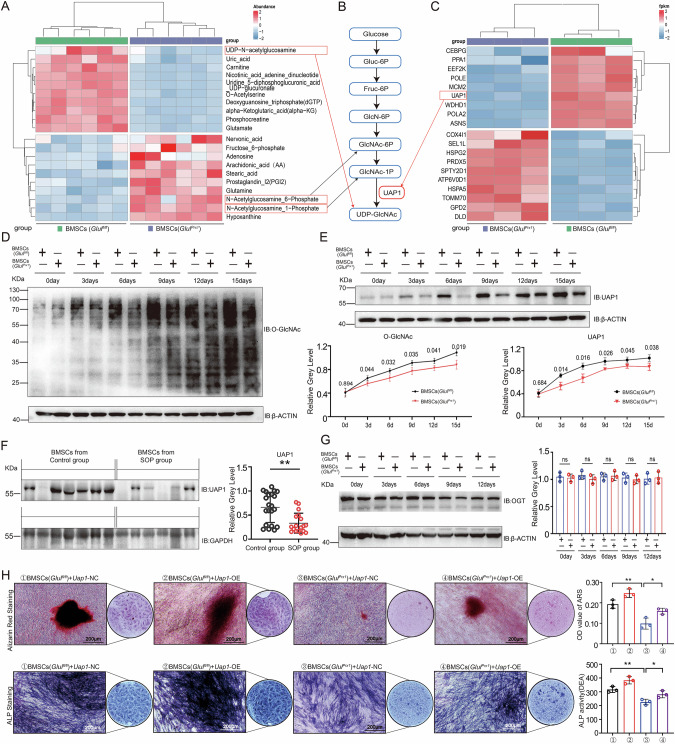
GLUL regulates HBP metabolism and affects UDP-GlcNAc generation. **A** Heatmap of metabolomic assays of the BMSCs from *Glul*^*Prx1*^ and *Glul*^*fl/fl*^ mice (*n *= 6). **B** Schematic diagram of the hexosamine biosynthetic pathway. **C** Heatmap of the proteomics analysis of BMSCs from *Glul*^*Prx1*^ and *Glul*^*fl/fl*^ mice (*n *= 3). **D** Western blotting analysis of O-GlcNAcylation levels in BMSCs from *Glul*^*Prx1*^ and *Glul*^*fl/fl*^ mice at different osteogenic differentiation timepoints. The numbers on the line graph represent the *P *-values comparing the relative protein expression levels between the two groups. **E** Western blotting analysis of UAP1 expression in BMSCs from *Glul*^*Prx1*^ and *Glul*^*fl/fl*^ mice at different osteogenic differentiation timepoints. The numbers on the line graph represent the *P *-values comparing the relative UAP1 expression levels between the two groups. **F** Western blotting analysis of UAP1 expression in BMSCs from patients in the SOP group (*n *= 6) and the control group (*n *= 7). **G** Western blotting analysis of OGT levels in BMSCs from *Glul*^*Prx1*^ and *Glul*^*fl/fl*^ mice at different osteogenic differentiation timepoints. **H** Representative images of ARS and ALP staining of BMSCs from *Glul*^*Prx1*^ and *Glul*^*fl/fl*^ mice after transfection with the UAP1 overexpression or control plasmid. **P < 0.01 vs. other groups; *P < 0.05 vs. other groups.

Next, we investigated the specific mechanism by which GLUL regulates osteogenic differentiation. We performed proteomic and metabolomic assays on BMSCs from *Glul*^*Prx1*^ and *Glul*^*fl/fl*^ mice after osteogenic differentiation. Component analysis revealed that *Glul* KO significantly altered the intracellular metabolic characteristics of BMSCs (Fig. S3A-C). The results of liquid chromatography‒mass spectrometry (LC‒MS/MS) revealed a significant decrease in the levels of UDP-GlcNAc (HBP metabolic end product) in *Glul*-KO cells (Figs. 3A and S3B). Surprisingly, the levels of Fruc-6P, GlcNAc-6P, and GlcNAc-1P, which are intermediate products of HBP metabolism, exhibited opposite trends. These findings indicate that the key regulatory point that affects the generation of UDP-GlcNAc in *Glul*-KO cells is the final step of the HBP metabolic pathway (Fig. 3A, B), in which UAP1 catalyses the generation of UDP-GlcNAc from GlcNAc-1P.

By analysing proteomic data, we found that UAP1 expression was downregulated in *Glul*-KO cells (Fig. 3C), and this result was confirmed by Western blotting (Fig. 3E). UAP1 expression also tended to decrease in BMSCs from SOP patients (Fig. 3F). Moreover, there was no significant change in OGT expression (Fig. 3G). Therefore, GLUL affects HBP flux by regulating UAP1 expression. Research has shown that changes in the UDP-GlcNAc concentration can directly affect the level of protein O-GlcNAcylation [22]. We found that during the osteogenic differentiation of BMSCs, the overall level of protein O-GlcNAcylation increased (Fig. 3D), and the expression level of UAP1 also tended to increase (Fig. 3E). To some extent, supplementation with UAP1 rescued the disordered osteogenic differentiation of *Glul*-KO cells (Figs. 3H and S3E). Therefore, GLUL regulates UDP-GlcNAc biosynthesis by affecting the UAP1 expression level, thereby regulating protein O-GlcNAcylation and affecting BMSC osteogenic differentiation.

### GLUL regulates UAP1 expression by interacting with Tripartite Motif Containing 25 (TRIM25)

**Fig. 4:**
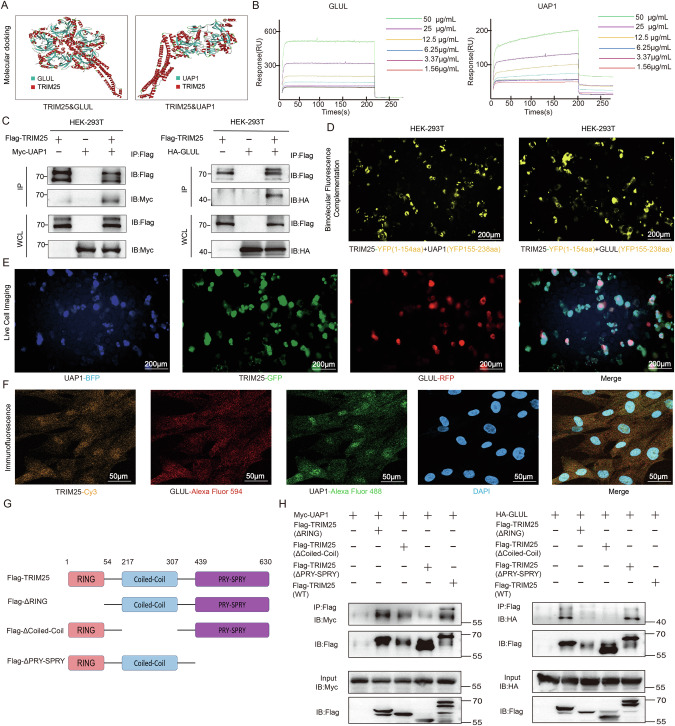
GLUL competently binds to the TRIM25-SPRY subunit and UAP1. **A** Molecular docking image of the TRIM25 protein and the GLUL/UAP1 protein. **B** The results of the Biacore assay to assess the binding strength of GLUL/UAP1 to TRIM25. **C** Representative co-IP images of TRIM25 with GLUL/UAP1 in HEK-293T cells. **D** Representative images of bimolecular fluorescence complementation experiments between TRIM25 carrying YFP (aa 1–154) and GLUL/UAP1 carrying YFP (aa 155–238). **E** Representative images of live-cell imaging after cotransfection of GFP-TRIM25, RFP-GLUL, and BFP-UAP1 into HEK293T cells. **F** Representative images of immunofluorescence staining for TRIM25, GLUL and UAP1 in BMSCs. **G** Schematic diagram of TRIM25 truncation. **H** Representative images of co-IP experiments conducted with Myc-UAP1, HA-GLUL, and Flag-TRIM25 full-length and truncated samples. **P < 0.01 vs. other groups; *P < 0.05 vs. other groups.

To explore the mechanism by which GLUL regulates UAP1 expression, we consulted the NCBI database and found that TRIM25 and TRIM67 are potential interacting proteins between GLUL and UAP1 (Fig. S4A). The molecular docking results suggest that TRIM25 has potential binding sites with GLUL and UAP1 (Fig. 4A). Biacore experiments confirmed the interaction between the TRIM25 protein and GLUL or UAP1 (Fig. 4B). Moreover, co-immunoprecipitation (co-IP) experiments were conducted with the HEK293T cell line and BMSCs. The results revealed that TRIM25 coimmunoprecipitated with exogenous and endogenous GLUL and UAP1 (Figs. 4C and S4C). The bimolecular fluorescence complementation (BiFC) results suggested that, owing to the interaction of TRIM25 with GLUL or UAP1, the two molecular fragments of the YFP carried by TRIM25 reformed active fluorescent groups (Fig. 4D and Videos 1 and 2). The interactions of the three molecules in living cells were also recorded (Fig. 4E and Video 3). In addition, endogenous TRIM25 was shown to colocalize with GLUL and UAP1 in the cytoplasm of BMSCs (Fig. 4F). The molecular structure of TRIM25 includes a RING Finger domain (aa 1–54), a coiled coil domain (aa 217–307), and a SPRY domain (aa 389–630) (Fig. 4G). We conducted co-IP experiments on different TRIM25 mutants, which are shown in the figure. The absence of the SPRY domain caused the binding affinity of TRIM25 for GLUL or UAP1 to be lost (Fig. 4H). Therefore, the SPRY domain is necessary for the interaction of TRIM25 with GLUL or UAP1. We hypothesize that the competitive binding of GLUL to the TRIM25 SPRY domain inhibits its interaction with UAP1.

**Fig. 5:**
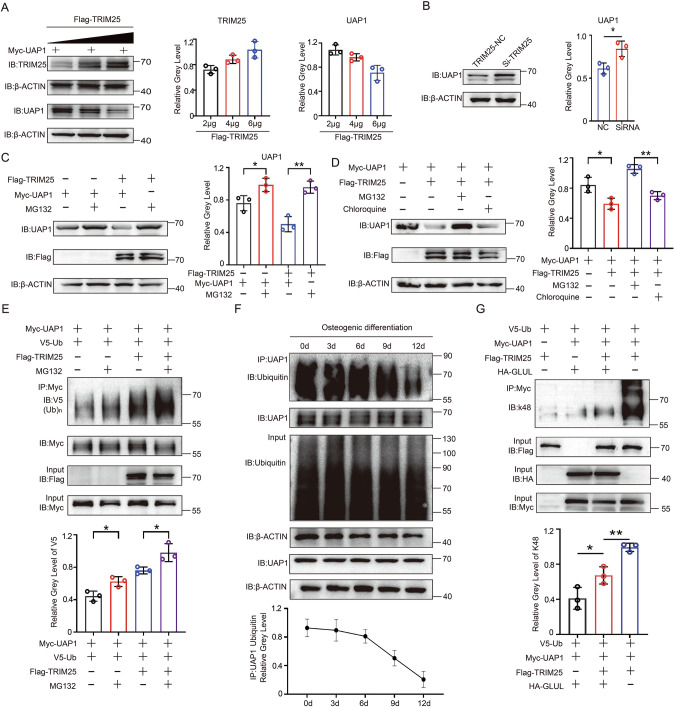
GLUL reduces the K48 ubiquitin connectivity level of TRM25 to UAP1. **A** Representative Western blotting images of HEK-293T cells cotransfected with the TRIM25 overexpression vector and UAP1 in a dose-dependent manner. **B** Representative image of a Western blotting experiment showing UAP1 expression levels after cell transfection with si-TRIM25. **C** Representative image of a Western blotting experiment showing UAP1 expression levels after cell transfection with si-TRIM25. **D** Representative images of Western blotting analysis of proteins that were extracted from HEK293T cells that were transfected with the Myc-UAP1 and Flag-TRIM25 overexpression plasmids and treated with MG132. **E** Representative images of Western blotting analysis of proteins that were HEK293T cells that were transfected with the Myc-UAP1 and Flag-TRIM25 overexpression plasmids and treated with MG132 and chloroquine. **F** A Myc-UAP1 overexpression plasmid was transfected into BMSCs, and osteogenic differentiation was induced. Proteins were extracted from the cells at different timepoints, and representative images were obtained after co-IP experiments with anti-UAP1 antibodies. **G** The V5 Ub overexpression plasmid was transfected into HEK293T cells, which were then cotransfected with Flag-TRIM25, Flag-TRIM25+Myc-UAP1 + HA-GLUL, or Myc-UAP1 + HA-GLUL overexpression plasmids. Subsequent Western blot images and quantitative analysis revealed the  K48-linked ubiquitination of UAP1 in different cell groups. **P < 0.01 vs. other groups; *P < 0.05 vs. other groups.

Research has shown that TRIM25 promotes substrate degradation through the ubiquitin proteasome pathway. We investigated whether TRIM25 regulates the degradation of UAP1. The results indicate that the level of TRIM25 was inversely proportional to the level of the UAP1 protein (Figs. 5A, B and S4B). In addition, TRIM25 significantly reduced UAP1 expression in a dose-dependent manner (Fig. 5A). When the proteasome inhibitor MG132 was added, the level of the UAP1 protein in HEK293T cells was significantly increased (Fig. 5C), but the lysosome inhibitor chloroquine did not restore UAP1 expression (Fig. 5D). These results indicate that TRIM25 promotes UAP1 degradation through the proteasome pathway. Lys48-linked polyubiquitin chains target proteins for degradation via proteasome-dependent processes [28]. The interaction between TRIM25 and UAP1 led to the induction of UAP1 Lys48 ubiquitination (Fig. 5E), whereas during BMSC osteogenic differentiation, UAP1 Lys48 ubiquitination gradually decreased (Fig. 5F). In this process, GLUL reduces TRIM-mediated the Lys48 ubiquitination of UAP1 (Fig. 5G), which is the specific mechanism by which TRIM25 regulates UAP1 expression.

### O-GlcNAcylation of FOXO3-Ser296, which is regulated by GLUL, promotes the osteogenic differentiation of BMSCs

**Fig. 6:**
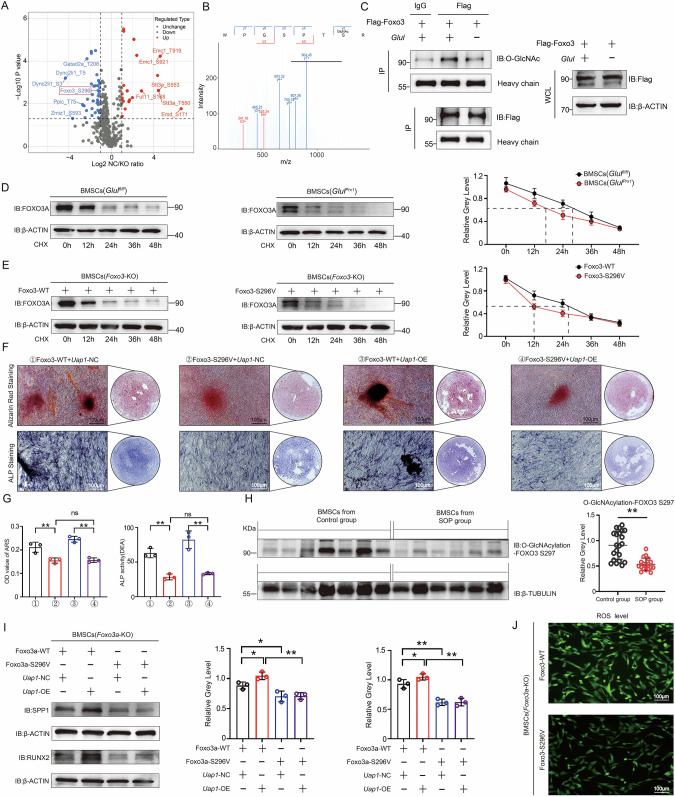
O-GlcNAcylation of FOXO3-Ser296, which is regulated by GLUL, promotes the osteogenic differentiation of BMSCs. **A** Volcano plots of BMSCs from *Glul*^*Prx1*^ (*n *= 3) and *Glul*^*fl/fl*^ (*n *= 3) mice subjected to 4D DIA O-GlcNAcylation profiling analysis. **B** Mass spectrometry image of the O-GlcNAcylation modification of the FOXO3 Ser296 residue. **C** The results of the co-IP experiment on the effect of Glul knockout on the level of O-GlcNAcylation modification of FOXO3 in BMSCs. **D** Representative images of target protein decay at different time points in BMSCs from *Glul*^*Prx1*^ and *Glul*^*fl/fl*^ mice. **E** Representative images of target protein attenuation at different time points were obtained by transfecting wild-type or S296V-mutant FOXO3 plasmids into *Foxo3*-KO BMSCs. **F** Representative images of ARS and ALP staining of *Foxo3*-KO BMSCs after transfection with wild-type or S296V-mutant FOXO3 plasmids. **G** Quantitative analysis of ARS and ALP staining. **H** Representative images of FOXO3-Ser297 O-GlcNAcylation levels as determined by Western blotting analysis of BMSCs from patients in the SOP and control groups. **I** Western blotting analysis of the expression of osteogenesis-related genes in *Foxo3*-KO BMSCs after transfection with wild-type or S296V-mutant FOXO3 plasmids. **J** Representative images of ROS detection. **P < 0.01 vs. other groups; *P < 0.05 vs. other groups.

To further explore how GLUL-mediated regulation of protein O-GlcNAcylation affects the osteogenic differentiation of BMSCs, we used 4D DIA O-GlcNAcylation omics analysis (PTM BIO, Hangzhou, China) to identify O-GlcNAcylation-modified proteins specifically regulated by GLUL. A total of 73 candidate proteins and 129 modification sites were identified (Figs. 6A, B and S5A -F). We subsequently focused on FOXO3, which is closely associated with processes such as cellular ageing and osteogenic differentiation. The Flag-*Foxo3* overexpression plasmid was transfected into BMSCs (*Glul*^*fl/fl*^) and BMSCs (*Glul*^*Prx1*^), anti-Flag antibody was used for co-IP to assess O-GlcNAcylation levels [29, 30]. The result confirmed that the O-GlcNAcylation level of FOXO3 in BMSCs was reduced upon *Glul* knockout or *Uap1* knockdown (Figs. 6C and S5H-I). The omics analysis suggested that Ser296 (S296) is the main O-GlcNAcylation modification site in FOXO3. S296 and surrounding amino acid sequences are highly conserved in vertebrates (Fig. S5G). Motif analysis of O-GlcNAcylation modification sites revealed that the polar residue (SADDSPSTSSKWPGSPTSRSSDE), in which FOXO3-Ser296 is located, conforms to the XxxxxxxSxxSRxxxxxxxxxxxx motif (Fig. S5B-C, Supplementary Table 1). Next, we generated a site-specific mutant of FOXO3 (S296V) and screened BMSCs with CRISPR/Cas9 to knock out *Foxo3* (Fig. S5J). We conducted a series of actinomycete ketone tracking experiments to evaluate the stability of FOXO3. Compared with that in cells that were transfected with the empty vector, the half-life of FOXO3 in *Glul*-KO cells was shorter (Fig. 6D). Compared with wild-type cells, the S296V mutation shortened the half-life of FOXO3 from 24 hours to 12 hours (Fig. 6E). These results indicate that O-GlcNAcylation of S296 can stabilize FOXO3.

To further investigate whether the absence of O-GlcNAcylation in FOXO3-S296 affects BMSC osteogenic differentiation, we transiently overexpressed WT *Foxo3* or the S296V mutant in *Foxo3*-KO BMSCs and induced osteogenic differentiation. Alizarin Red and ALP staining indicated that the *Foxo3* S296V mutation weakened the osteogenic differentiation potential of the BMSCs, and this effect was not reversed by UAP1 (Fig. 6F, G). Correspondingly, the expression of osteogenic marker genes (RUNX2 and SPP1) was downregulated (Fig. 6I). *Foxo3* S296V mutation leads to increased levels of reactive oxygen species (ROS) in BMSCs (Fig. 6J). We generated anti-O-GlcNAcylation antibodies targeting the corresponding site in human FOXO3 (S297) and performed Western blotting to measure the levels of this modification in BMSCs from SOP patients. The results showed that in BMSCs from SOP patients, the levels of the FOXO3-S297O-GlcNAcylation modification were decreased (Fig. 6H). Overall, the O-GlcNAcylation of FOXO3-Ser296, which is regulated by GLUL, stabilizes FOXO3 and promotes BMSC osteogenic differentiation in vitro, and dysregulation of this pathway occurs in SOP patients.

### Supplementation of GLUL can alleviate bone loss in SOP model mice

**Fig. 7:**
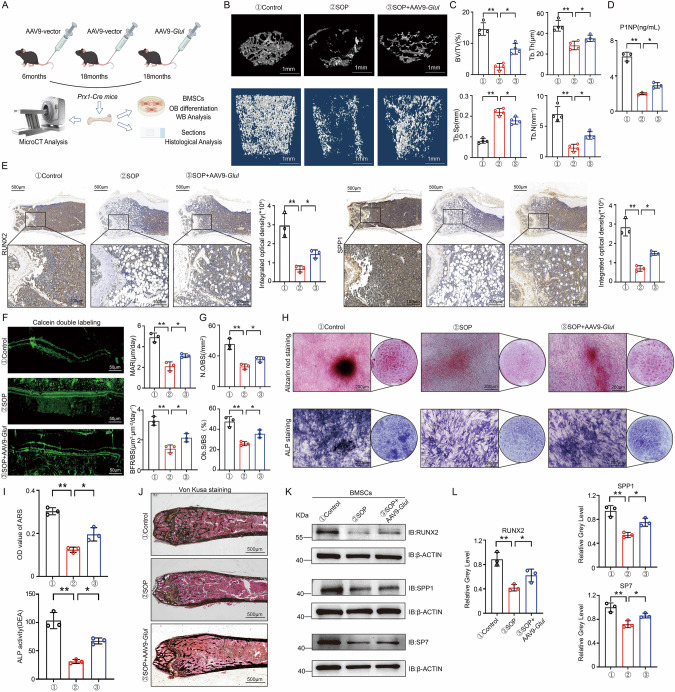
Supplementation of GLUL can alleviate bone loss in SOP model mice. **A** Schematic diagram of in vivo experiment. **B** Representative micro-CT images of trabecular bone from the femoral metaphysis of mice(*n *= 4). **C** Quantitative analysis of BV/TV, Tb.Th, Tb.N and Tb.Sp. **D** ELISA detection results of serum P1NP levels in each group of mice (*n *= 4). **E** Immunohistochemical staining of osteogenesis-related genes in mice femur sections. (*n *= 3). **F** Calcein double-label staining image and quantitative analysis of the MAR and BFR/BS in each group of mice (*n *= 3). **G** Analysis of Osteoblast Density (N.O/BS) and Osteoblast Surface Area (O.S/BS) Data. **H** Representative images of ARS and ALP staining of BMSCs from mice of each group. **I** Quantitative analysis of ARS and ALP staining. **J** Representative images of VON KOSSA staining (*n *= 3). **K** Western blot analysis of the expression of osteogenesis-related genes in BMSCs from mice of each group. **L** Quantitative data analysis of Western blot results. **P < 0.01 vs. other groups; *P < 0.05 vs. other groups.

To evaluate the potential role of GLUL in bone loss in SOP, *Glul*-overexpressing adenovirus (aav) was injected into 18-month-old *Prx1-*Cre mice via the tail vein. Micro-CT analysis revealed that *Glul* overexpression reduced bone loss in SOP model mice (Fig. 7A–C), and the P1NP serum concentration increased (Fig. 7D), indicating increased osteoblast activity. Furthermore, immunohistochemical staining suggested that the expression of osteogenic marker genes was increased (Fig. 7E). Dual-labelling experiments with calcein confirmed that *Glul* overexpression increased bone formation (Fig. 7F). Similarly, after *Glul* overexpression, the number and surface area of osteoblasts significantly increased (Fig. 7G). To evaluate the effect of GLUL supplementation on BMSC osteogenic differentiation, BMSCs were isolated from the femurs of *Prx1*-Cre mice in the control, SOP, and *Glul*-OE aav groups. The results revealed that GLUL expression was downregulated in the BMSCs of SOP model mice, whereas *Glul*-OE aav promoted BMSC osteogenic differentiation and bone formation (Fig. 7H–J). Moreover, GLUL overexpression increased osteogenic marker genes expression in the BMSCs of SOP model mice (Fig. 7K, L). In summary, these results indicate that supplementation with GLUL can alleviate bone loss in SOP model mice.

## Discussion

New evidence suggests that the lack of GLUL is one of the characteristics of BMSCs from SOP patients and that the weakened osteogenic differentiation of BMSCs is an important cause of the occurrence and development of SOP [31]. Promoting the osteogenic differentiation of BMSCs is considered a promising strategy for ameliorating the symptoms of SOP [32]. Our research indicates that the absence of GLUL in BMSCs leads to abnormal development of the skull and phalanges of mice, as well as reduced bone mass in adult mice. Moreover, targeted GLUL supplementation in BMSCs alleviated bone loss in SOP model mice. Therefore, GLUL plays an indispensable role in the osteogenic differentiation of BMSCs. In summary, these data provide the first example of GLUL regulating lineage allocation and bone homeostasis in BMSCs.

Although GLUL is a key enzyme in glutamine synthesis, roles for GLUL independent of metabolic enzymes in gastric cancer, lung cancer, epilepsy, and angiogenesis have been revealed by multiple studies [7, 33–35]. We discovered the unique role of GLUL in regulating UDP-GlcNAc synthesis. Previous studies have shown that in hepatocellular carcinoma, the mechanism by which GLUL increases the HBP flux is that GLUL increases glutamine synthesis via its enzymatic activity, providing a nitrogen source for UTP synthesis and thereby increasing UDP-GlcNAc generation [22]. During the osteogenic differentiation of BMSCs, knocking out *Glul* and supplementing glutamine cannot rescue the abnormal differentiation phenotype. By analysing the synthesis of various intermediate products in the HBP pathway, we ultimately identified and revealed that UAP1 is the regulatory point for GLUL synthesis in BMSCs. Additionally, in this process, the role of GLUL is independent of its catalytic activity.

O-GlcNAcylation is a posttranslational modification that is involved in important functions such as glucose metabolism, transcriptional regulation, subcellular localization, enzyme activity, and immune regulation, and it plays a regulatory role in determining the differentiation fate of bone marrow stromal cells [36–38]. During this process, OGT mediates the O-GlcNAcylation of the transcription factor RUNX2 and supports osteogenic differentiation [21]. In this study, there was no difference in the expression of OGT between *Glul-*KO and control cells. Although OGT is the only enzyme that catalyses this modification, our results suggest that other mechanisms are involved in the decrease in protein O-GlcNAcylation caused by GLUL downregulation. We found that the UAP1-catalysed synthesis of glycosylation substrates plays a more significant regulatory role in this process. However, how UAP1 is regulated by ubiquitination is largely unknown.

FOXO3 is a key factor in the cellular defence against oxidative stress, and it is equally important for the osteogenic differentiation of BMSCs [39–41]. During the differentiation of BMSCs, the increase in mitochondrial respiration leads to an increase in endogenous reactive oxygen species level [42–44]. The instability or downregulation of FOXO3 causes the ability of BMSCs to control ROS levels to be lost, thereby impairing differentiation [45, 46]. Here, we first describe the important role of GLUL in regulating the O-GlcNAcylation of FOXO3 at the S296 site during SOP progression. The modification of this site helps to stabilize FOXO3, thereby participating in the regulation of BMSC differentiation.

This study has certain limitations. The reason for the downregulation of GLUL in the BMSCs of SOP patients has not been revealed. The mechanism by which GLUL specifically regulates the O-GlcNAcylation of FOXO3 is also unclear. Moreover, the mechanism by which S296 O-GlcNAcylation mediates FOXO3 stability has not been extensively explored.

Overall, we revealed a link between disordered BMSC osteogenic differentiation and O-GlcNAcylation in SOP, and we demonstrated that GLUL can promote HBP-mediated UDP-GlcNAc biosynthesis by reducing UAP1 ubiquitination-mediated degradation. In addition, GLUL promotes O-GlcNAcylation at Ser296 through the mechanism described above to stabilize FOXO3 and maintain its control over ROS during BMSC osteogenic differentiation. In addition, these findings increase our understanding of the role of O-GlcNAcylation in SOP pathogenesis and progression and suggest that targeting GLUL in BMSCs may be a strategy for treating SOP.

## Materials and methods

### Reagents and antibodies

The Disodium beta-glycerol phosphate pentahydrate (S27785) was purchased from Shanghai yuanye Bio-Technology Co., Ltd (Shanghai, China). Ascorbic acid (A92902) and dexamethasone (D4902) were obtained from Merck (Darmstadt, Germany). BCIP/NBT Alkaline Phosphatase Color Development Kit and Alkaline Phosphatase Assay Kit were obtained from Beyotime Biotechnology (Shanghai, China). Cy3 goat anti-mouse IgG (A22210), DyLight 488 goat anti-rabbit IgG (A23220) and DyLight 549 goat anti-mouse IgG (A23310) were obtained from Abbkine Scientific (Wuhan China). Cycloheximide (GC17198) were obtained from GLPBIO (Montclair, USA), and MG-132 (HY-13259), Thiamet G (HY-12588), Chloroquine (HY-17589A) were obtained from MedChemExpress LLC (New Jersey, USA). TRIM25 (NM_005082) Human Recombinant Protein, Glutamine Synthetase (GLUL) (NM_002065) Human Recombinant Protein and UAP1 (NM_003115) Human Recombinant Protein were obtained from OriGene (Maryland, USA). Biacore Series S Sensor Chip CM5 were obtained from Cytiva (Washington, D.C. USA).

The information on the primary antibodies applied in this research are listed in Supplementary Table 2.

### Mice

Conditional *Glul*-KO (*Glul*^fl/fl^) mouse (S-CKO-02655) and *Prx1-Cre* mouse (C001029) strains were obtained from Cyagen Biotechnology Co., Ltd. These strains were generated as previously described [47–49]. *Glul*^fl/fl^ mice were crossed with the *Prx1-Cre* strain to generate *Glul*^*Prx1*^ mice. During the breeding process, the mice were maintained on the C57BL/6 background. The mice were fed in an air-conditioned room at 23–25 °C with 12-hour light‒dark cycles and free access to water and food. The animal experiments were carried out in strict accordance with protocols approved by the Medical Science Research Ethics Committee of the Affiliated Hospital of Jining Medical University.

### Alizarin Red-Alcian Blue double staining

On the first day after birth, neonatal mice were euthanized via the injection of pentobarbital and fixed with 95% alcohol for at least 48 hours. After termination of fixation, the skin and internal organs of the neonatal mice were carefully removed and fixed in acetone for one week. Alizarin Red Alizarin Blue staining solution was prepared as follows: 0.3% Alizarin Blue, 0.1% Alizarin Red, glacial acetic acid, and 70% ethanol were mixed in a 1:1:1:17 ratio. The samples were then placed in the staining solution and incubated for 24–48 hours. The samples were then washed with pure water, soaked in 1% KOH solution for 48 hours, soaked in 20% glycerol for one week, transferred to 50% glycerol, and photographed with a Zeiss Axio Zoom. V16 microscope.

### Bone dynamic analysis

The mice were intraperitoneally injected with calcein (20 mg/kg) 10 and 3 days before sacrifice. After sampling, the femur was embedded, and nondecalcified sections were prepared. These sections were observed and photographed using a fluorescence microscope (Zeiss Axio Zoom V16).

### Micro-CT

After the mice were euthanized, the femurs were separated and fixed with 4% paraformaldehyde. A micro-CT imaging system (PerkinElmer; Quantum GX2) was used to scan femoral tissue. The scanning parameters were set as follows: voltage of 90 kV, 88 μA, 14 minutes, and resolution of 36 μm pixels. Skyscan NRecon software was used to reconstruct the images, and CTVox software was used to analyse the sample parameters.

### Bone histomorphometry and immunohistochemistry

All the procedures were carried out as previously reported [50]. The static reconstruction parameters in Goldner three-colour stained sections were quantified using Osteomeasure (OsteoMetrics, Inc., US) software. All the histomorphological parameters were measured in accordance with the recommendations of the ASBMR Nomenclature Committee.

### Lentivirus-mediated KO and aav-mediated overexpression

The *Foxo3*-KO lentivirus based on CRISPR/Cas9 technology was provided by Shanghai Genechem Co., Ltd. *Glul*- aav was also purchased from the same company. The *Glul*-aav construction strategy was as follows: CAG-FLEX-EGFP-FT2A-NM_008131-3Flag-SV40 Polya. The *Foxo3*-KO lentiviral construction strategy was as follows: GV392-U6-sgRNA-Foxo3-EF1a-Cas9-FLAG-P2A-puro. For lentivirus transfection, 80% confluent cells were incubated with lentivirus and polybrene (8 μg/mL), puromycin (P8230, Solarbio) was used for screening, and target gene expression was measured by Western blotting.

### Plasmid and siRNA transfection

For a single well of a six-well plate, 4 µg of plasmid and 10 μL of Lipofectamine 2000 (11668019; Thermo Fisher Scientific) were mixed with 250 μL of Opti-MEM I (31985070; Thermo Fisher Scientific), and the mixture was incubated for 5 minutes at room temperature. During transfection, 1.5 mL of Opti-MEMI and transfer solution were added to a single well of a six-well plate. After 4 hours, the transfection mixture was removed and replaced with fresh medium. Analysis of overexpression efficiency and other experiments were performed 48 hours after transfection. Plasmids overexpressing Flag-*TRIM25*, Myc-*UAP1*, HA-*GLUL*, V5-Ubiquitin, Flag-*Uap1*, Flag-*Foxo3*-WT, Flag-*Foxo3*-S296V, Flag-*FOXO3*-WT, Flag-*FOXO3*-S297V, Flag-*TRIM25*(Δ13-54aa), Flag-*TRIM25*(Δ217-307aa), Flag-*TRIM25*(Δ439-630aa), GFP-*TRIM25*, RFP-*GLUL*, and BFP-*UAP1*, were synthesised by WZ Biosciences Inc. Plasmids overexpressing YFP(1-154aa)-TRIM25, YFP(155-238aa)-UAP1, YFP(155-238aa)-GLUL were synthesised by GenePharma Corporation.

For *TRIM25*-siRNA (GenePharma) transfection, the procedure was the same as that for plasmid transfection. The amounts of plasmid and Lipofectamine 2000 that were used were 100 pmol and 5 μL, respectively.

### Quantitative real-time polymerase chain reaction

Quantitative real-time polymerase chain reaction (RT-qPCR) was performed as previously described [51].

### Western blotting and co-immunoprecipitation

Western blotting was performed according to previously described methods [52]. After electrophoresis and membrane transfer, the PVDF membranes were shaken and incubated overnight with the primary antibody at 4 °C. The next day, the PVDF membranes were incubated at room temperature with HRP-labelled Goat Anti Rabbit IgG or Goat anti Mouse IgG (1:5000) for 1 hour. The PVDF membrane was subsequently washed thoroughly. A Tanon-5200 chemiluminescence imaging system (Shanghai, China) and ImageJ software (National Institutes of Health, USA) were used to record and quantify the signal intensities. The protein levels were normalized to those of β-actin (1:3000) as a control.

For co-IP, whole-cell extracts were lysed with Pierce IP lysis buffer (87787; Thermal Science). Then, the extraction solution and the corresponding antibodies were added and incubated with the extracts overnight at 4 °C. Then, Protein A&G beads (Bersinbio) were added, and the solutions were incubated at 4 °C for 4 hours. The coprecipitated proteins were washed with SDS loading buffer at 95 °C for 5 minutes. The subsequent results were obtained using Western blotting, as described above. The difference was that a Mouse anti-Rabbit IgG (Conformation Specific) mAb (HRP Conjugate) (#5127, CST) was used as a secondary antibody.

### Generation of an anti-O-GlcNAc antibody targeting the S297 site of human FOXO3 (FOXO3 S297-O-GlcNAc)

Two modified peptide sequences were designed and synthesized, ensuring their immunogenicity and specificity. A negative unmodified peptide was designed for antibody detection. FOXO3 S297 O-GlcNAc-modified peptide A: C WPGSPT-(O-GlcNAc)S-RSSDEL 291-303; FOXO3 S297 O-GlcNAc-modified peptide B: PGSPT-(O-GlcNAc)S-RSSDE C 292-302; FOXO3 unmodified peptide: C WPGSPTSRSSDEL 291-303. The peptide samples were separately purified. These peptides were subsequently conjugated with KLH. Two New Zealand white rabbits were immunized with each peptide, for a total of four rabbits. The immunization method involved injecting the antigens into two subcutaneous points on both shoulders and two muscle points on both hind legs. A total of six immunizations were performed before and after the fourth immunization, and blood samples were collected four times. The antibody titres in the serum were measured by ELISA, and Western blotting was performed on the serum. When the serum (OD450 > 1.0) reached 1:10000 (after the third or fourth immunization), it was considered to meet the standard, and subsequent purification was carried out. On the basis of the ELISA and Western blotting results, 2-4 rabbit serum samples were selected for two-step affinity purification of Protein A and antigen peptides. The specificity and viability of the antibodies were confirmed by Western blotting with FOXO3- and S297-mutant FOXO3-overexpressing HEK293T cells.

### Surface plasmon resonance (SPR) binding assay

BiacoreT200 was used for the SPR experiments. The recombinant TRIM25 protein was fixed on the S-series sensor chip CM5 (Cytiva). The protein was injected into the sample channel at 25 °C at a flow rate of 30 μL/min (180 seconds for association, 300 seconds for dissociation) to measure the binding of the recombinant proteins UAP1, GLUL and TRIM25. After each interaction analysis cycle, the sensor chip surface was completely regenerated for 10 s with 10 mM glycine-HCl as the injection buffer at a flow rate of 150 μL/min to remove the analyte.

### Immunofluorescence

All the procedures were carried out as previously reported [53], and images were captured with an LSM800 laser confocal microscope (Zeiss). To stain TRIM25, GLUL, and UAP1 simultaneously, on the first day, the cells were incubated with a rabbit anti-UAP1 antibody and a mouse anti-TRIM25 antibody. On the second day, the cells were incubated with Cy3-conjugated goat anti-mouse IgG and DyLight 488-conjugated goat anti-rabbit IgG. Then, the cells were incubated with an Alexa Fluor 647-conjugated anti-GLUL antibody again as the primary antibody.

### Statistics and reproducibility

Statistical analysis was performed using the unpaired two-tailed Student’s t-test, one-way ANOVA or two-way ANOVA, followed by post-hoc analysis using Tukey’s or Dunnet’s methods, were employed using Prism9 (GraphPad Software Inc., San Diego, CA, USA). All the data are shown as the means ± standard deviations (SDs). All the experiments were repeated at least three times unless otherwise indicated. A *p * value < 0.05 was considered statistically significant.

## Supplementary information

Supplementary Materials

Supplementary original blots

Video1

Video2

Video3

## Data Availability

The datasets generated during the current study are available in the Figureshare repository. 10.6084/m9.figshare.28105184.v1. All data supporting the findings of this study are available from the corresponding author upon reasonable request.
