# Supplementary material for: GLUL mediates FOXO3 O-GlcNAcylation to regulate the osteogenic differentiation of BMSCs and senile osteoporosis
Source: Cell Death Differ. 2025 Jul 11;32(12):2399–411. doi: 10.1038/s41418-025-01543-2 (PMC12669651; doi:10.1038/s41418-025-01543-2)
Supplement: Supplementary file 1 — Supplementary Materials [file 41418_2025_1543_MOESM1_ESM.docx]

Supplementary Materials for

GLUL mediates FOXO3 O-GlcNAcylation to regulate the osteogenic differentiation of BMSCs and senile osteoporosis

Lu Zhang, Bao Qi, Yanpeng Li, Xiao Liang, Zifang Zhang, Tao Yang, Shu Jia, Xu Gao, Shang Chen, Guangjun Jiao, Yangyang Li, Hongming Zhou, Yunzhen Chen, Yanming Li, Bin Zhang, Gang Li, Chunyang Meng *

Correspondence to: mengchunyang1600@mail.jnmc.edu.cn

**This file includes:**

Supplementary Methods

Supplementary Figure and Figure Legends

Video Descriptions

Supplementary Tables

**Supplementary Methods**

***Cell culture***

HEK293T cells were purchased from Shanghai Zhong Qiao Xin Zhou Biotechnology Co., Ltd. They were cultured in DMEM supplemented with 10% FBS and 1% penicillin/streptomycin. This cell line weas regularly tested negative for mycoplasma contamination and authenticated using STR DNA profiling.

***Preparation of osteogenic induction medium***

F12/DMEM complete medium containing 1% penicillin and streptomycin mixture and 10% fetal bovine serum was prepared. Next, 50μM ascorbic acid,10mM beta-glycerol phosphate and 100nM dexamethasone were added into the complete medium.

***Extraction of mouse bone marrow derived macrophage***

After euthanasia, the mice were sterilized in 75% ethanol for 5 minutes. The femur and tibia were extracted and placed in 75% alcohol for 5 minutes. The ethanol was washed off the surface of the bone tissue with PBS. The ends of the bone tissue were cut and the bone marrow was well flushed out with culture medium. The liquid was filtered into sterile tubes using a 70 μm cell filter and centrifuged at 1500 rpm/min for 5 min to precipitate the cells. Discarded supernatant and added erythrocyte lysate. Mixed thoroughly and left for 5 minutes. Centrifuged again and discarded the supernatant. Then cell suspensions were prepared with culture medium. Inoculated cells into culture plates. Half of the medium was replaced after 72 hours of incubation. All medium was replaced after 120 hours. After 168 hours, the cells were ready for experiments.

***ROS detection***

Stain cells with ROS sensitive dye DCFH-DA (10 mmol/L) at 37 ° C in the dark for 30 minutes. After washing with serum-free culture medium, the cells were observed and photographed under a fluorescence microscope (Zeiss)

***Metabolites Extraction of Cells***

The cell pellets (about 10^7^ cells) were taken, mixed with 1000 μL of extraction solution (MeOH: ACN: H2O, 2:2:1 (v/v)), the extraction solution contain deuterated internal standards, the mixed solution were vortexed for 30 s and incubated in liquid nitrogen for 1 min. The samples were then allowed to thaw at room temperature and vortexed for 30 s. This freeze–thaw cycle was repeated three times. Then the samples were sonicated for 10 min in 4 ℃ water bath, and incubated for 1 h at -40 ℃ to precipitate proteins. The samples were centrifuged at 12000 rpm (RCF=13800(×g), R= 8.6cm) for 15 min at 4 ℃. The supernatant was transferred to a fresh glass vial for analysis. The quality control (QC) sample was prepared by mixing an equal aliquot of the supernatant of samples.

***LC-MS/MS Analysis***

The cell pellets (about 10^7 cells) were taken, mixed with 1000 μL of extraction solution (MeOH: ACN: H2O, 2:2:1 (v/v)), the mixed solution was vortexed for 30 s and incubated in liquid nitrogen for 1 min. The samples were then allowed to thaw at room temperature and vortexed for 30 s. This freeze–thaw cycle was repeated three times.Then the samples were sonicated for 10 min in 4 ℃ water bath, and incubated for 1 h at -40 ℃ to precipitate proteins. The samples were centrifuged at 12000rpm (RCF=13800(×g), R= 8.6cm) for 15 min at 4 ℃. The supernatant was transferred to a fresh glass vial for analysis. The quality control (QC) sample was prepared by mixing an equal aliquot of thesupernatant of samples.

For polar metabolites, LC-MS/MS analysis was performed using a UHPLC system (Vanquish, Thermo Fisher Scientific). Connect Waters ACQUITY UPLC BEH amide (2.1 mm × 50 mm, 1.7 μ m) to an Orbitrap Exploris 120 mass spectrometer (Orbitrap MS, Thermo). The mobile phase consists of 25 mmol/L ammonium acetate and 25% ammonia solution in water (pH=9.75) (A) and acetonitrile (B). The automatic injection temperature is 4 ℃ and the injection volume is 2 μ L. The Orbitrap Exploris 120 mass spectrometer is used to collect MS/MS spectra in information dependent acquisition (IDA) mode under the control of acquisition software (Xcalibur, Thermo). In this mode, the collection software continuously evaluates the completeness Scan MS spectra. The ESI source conditions are set as follows: sheath gas flow rate of 50Arb, auxiliary gas flow rate of 15Arb, capillary temperature of 320 ℃, full MS resolution of 60000, MS/MS resolution of 15000, collision energy: SNCE 20/30/40, Spray voltage is 3.8 kV (positive) or 3.4 kV (negative) respectively.

***Modification enrichment***

Dissolve the peptide segment in IP buffer solution (100 mM NaCl, 1 mM EDTA, 50 mM Tris HCl, 0.5% NP-40, pH 8.0), transfer the supernatant to pre washed resin (PTM0954, Hangzhou Jingjie Biotechnology Co., Ltd.), Place on a rotating shaker at 4 ° C, gently shake and incubate overnight. After incubation, wash the resin four times with IP buffer solution and twice with deionized water. Finally, use 0.1% trifluoroacetic acid eluent to elute the peptide segments bound to the resin, three times in total. Collect the eluent and vacuum freeze dry it. After draining, desalt according to the C18 ZipTips instructions, vacuum freeze and dry, and provide for liquid chromatography-mass spectrometry analysis.

The peptide segments were dissolved in liquid chromatography mobile phase A and separated using the Easy-nLC1000 ultra-high performance liquid chromatography system. Mobile phase A is an aqueous solution containing 0.1% formic acid and 2% acetonitrile; Mobile phase B is an acetonitrile water solution containing 0.1% formic acid. Liquid phase gradient setting: 0-18 min, 9% -24% B; 18-22 mi, 24%-35%B; 22-26 min, 35%-90%B; 26-30 min, 90% B, maintain flow rate at 450 nl/min. The peptide segments were separated by an ultra-high performance liquid chromatography system and injected into a Capillary ion source for ionization before being subjected to timsTOF Pro mass spectrometry for data collection. The ion source voltage is set to 1.6 kV, and TOF is used for detection and analysis of peptide parent ions and their secondary fragments. The data collection mode uses the data independent parallel accumulation serial fragmentation (dia PASEF) mode, with the primary mass spectrometry scanning range set to 100-1700m/z. After one primary mass spectrometry acquisition, 8 PASEF mode acquisitions are performed. The secondary mass spectrometry scanning is in the range of 425-1025, with every 25 m/z window.

***Animal Grouping and Randomization***

Randomization was performed using a computer-generated random number table (GraphPad Prism 9.0). Each mouse received a unique identification number, and animals were sequentially allocated to groups based on random number sorting until balanced group sizes were achieved. Researchers performing subsequent analyses were blinded to group assignments throughout the experiment. This randomization protocol ensured equivalent baseline body weights and behavioral scores across all groups prior to intervention.

***Sample size***

The clinical sample size was determined based on preliminary data from our experiments. Using the observed effect sizes (e.g., differences in GLUL levels between SOP and control groups) with an alpha level of 0.05 and 80% power (beta = 0.20), we calculated the required sample size via GPower for two-tailed t-tests/ANOVA, as appropriate. The estimated effect sizes suggested n≥5/group would suffice.

The sample sizes in our animal experiments were chosen based on preliminary data from similar osteoporosis models in prior studies^1, 2^, and ethical considerations to minimize animal use while ensuring reproducibility.

**References**

1. Li C, Qiu M, Chang L, Qi J, Zhang L, Ryffel B et al. The osteoprotective role of USP26 in coordinating bone formation and resorption. *Cell Death Differ* 2022; **29**:1123-1136.

2. Li Q, Yue T, Du X, Tang Z, Cui J, Wang W et al. HSC70 mediated autophagic degradation of oxidized PRL2 is responsible for osteoclastogenesis and inflammatory bone destruction. *Cell Death Differ* 2023; **30**:647-659.

**Supplementary Figure and Figure Legends**


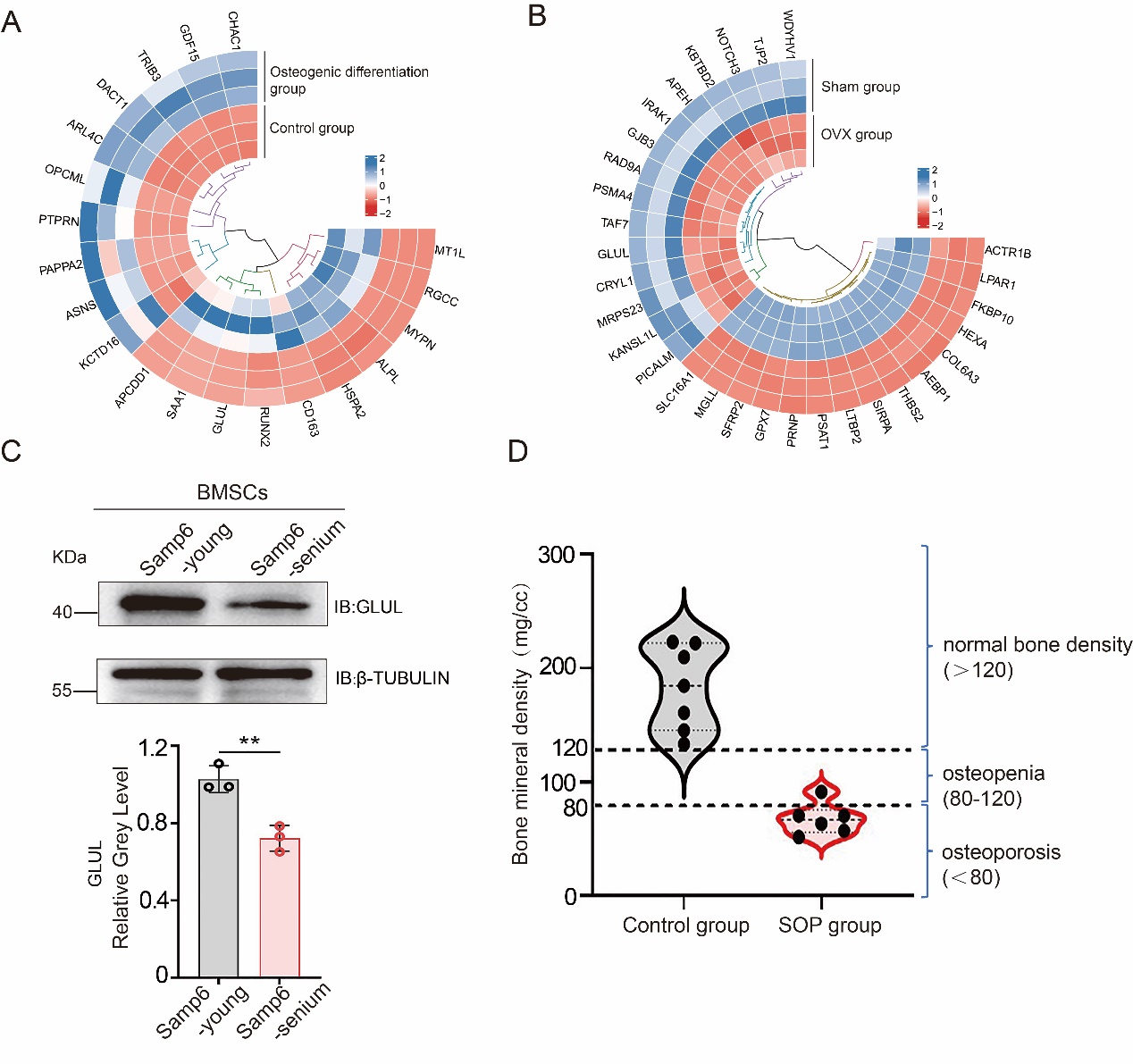


**Supplementary Fig. 1.** **GLUL plays an important role in the osteogenic differentiation of BMSCs and the progression of SOP**

A. Heatmap of the transcriptome expression of human bone marrow mesenchymal stem cells during osteogenesis according to the GSE178679 dataset. B. Heatmap of the transcriptome expression of bone marrow mesenchymal stem cells in postmenopausal osteoporosis models and in healthy control models according to the GSE 248225 dataset. C. Western blotting analysis of GLUL expression in BMSCs from young and old samp6 mice. D. Bone mineral density data of patients in control group (n=7) and SOP group (n=6). Abbreviations: ** represents P < 0.01 vs. other groups.


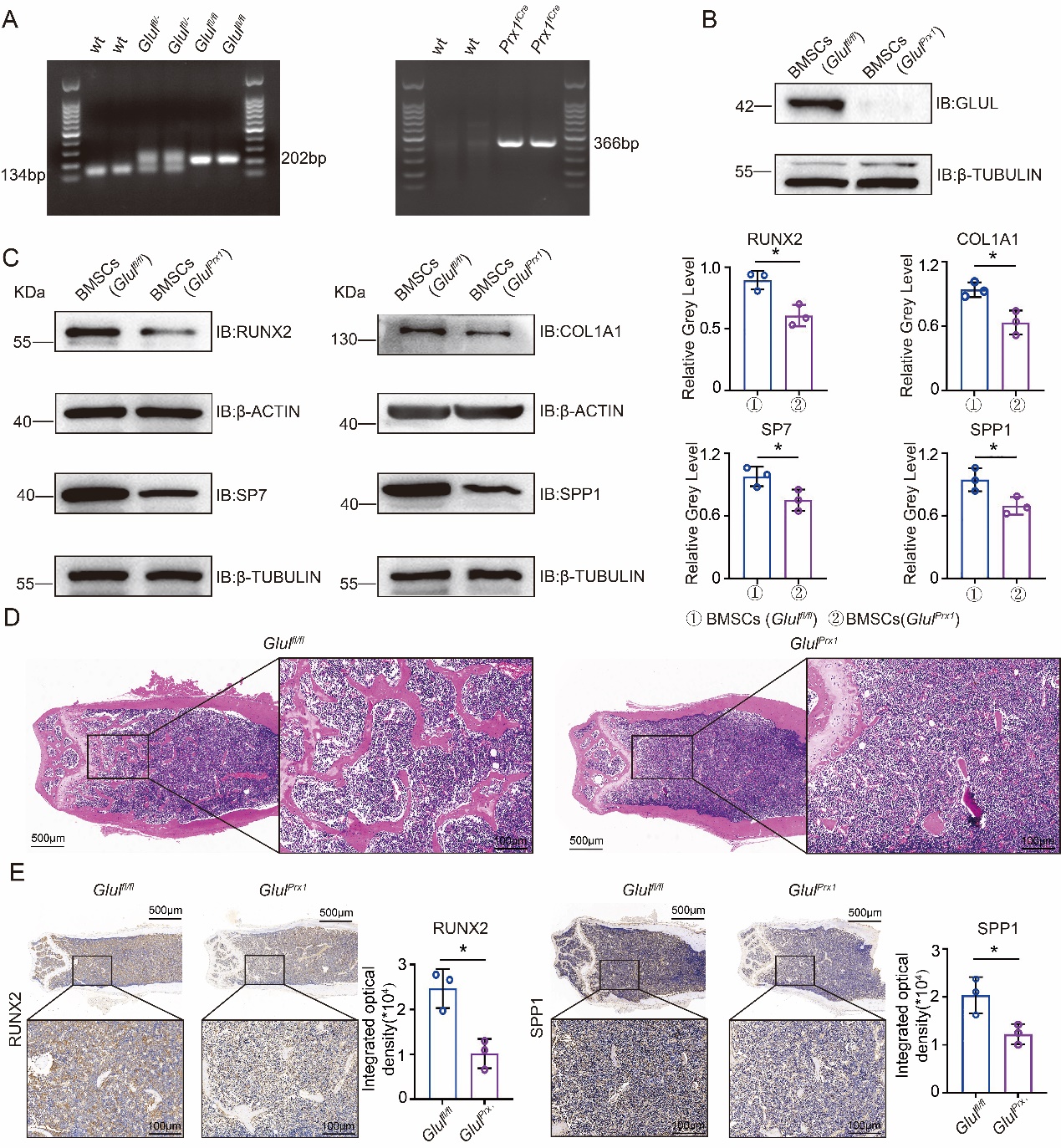


**Supplementary Fig. 2. Mesenchymal stem cell-specific Glul deficiency leads to abnormal bone formation**

A. Images of agarose gel electrophoresis identification of *Glul^Prx1^* mice. B. Western blotting analysis of GLUL expression in BMSCs from *Glul^Prx1^* and *Glul^fl/fl^* mice. C. Western blotting analysis of osteogenesis-related genes expression in BMSCs from *Glul^Prx1^* and *Glul^fl/fl^* mice. D. Haematoxylin-eosin (H&E) staining of femurs from the metaphysis of mice from *Glul^Prx1^* and *Glul^fl/fl^* mice (n=3). E. Immunohistochemical staining of osteogenesis-related genes in mice femur sections (n=3). Abbreviations: ** represents P < 0.01 vs. other groups; * represents P < 0.05 vs. other groups.

**
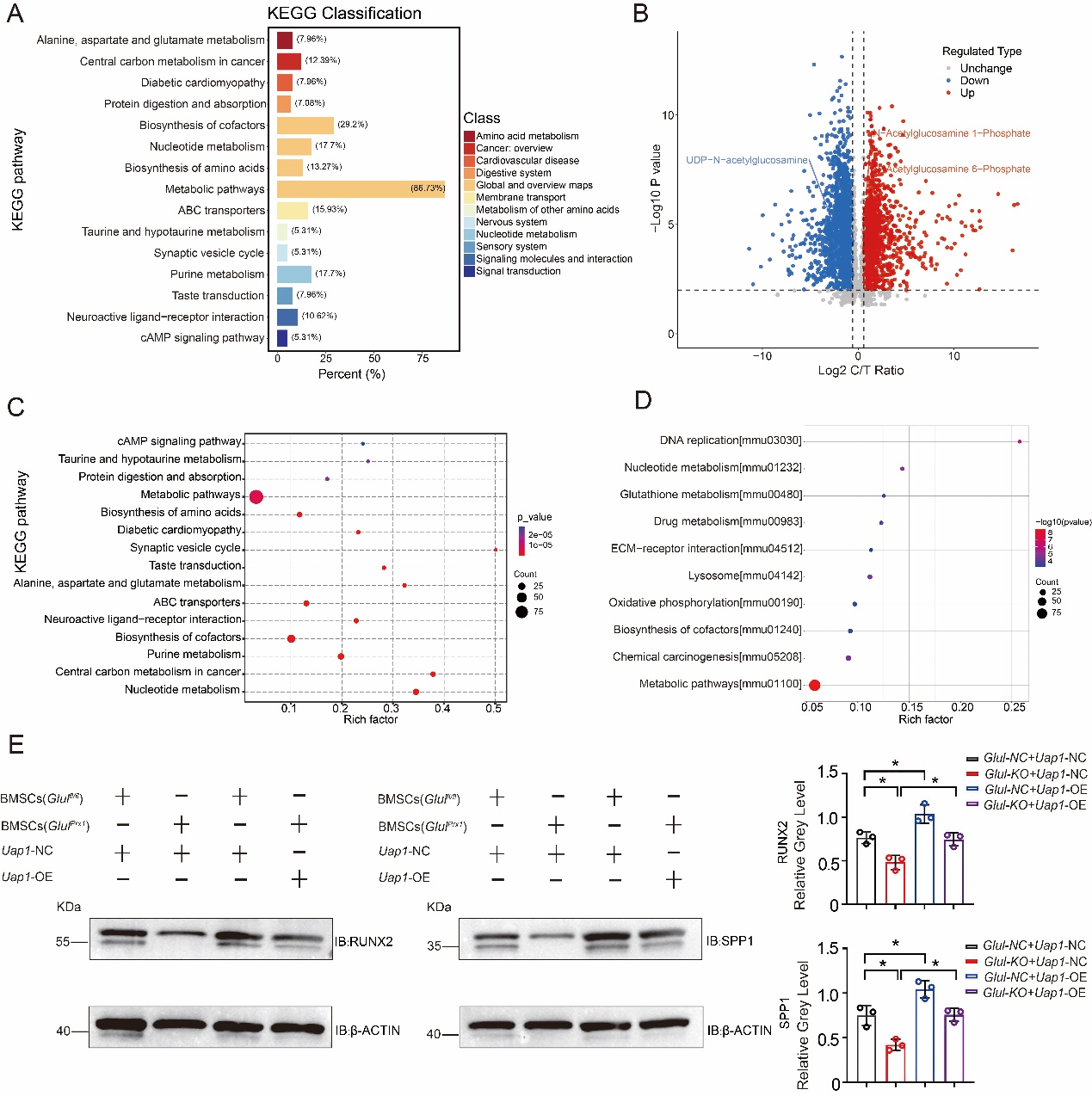
**

**Supplementary Fig. 3. GLUL regulates HBP metabolism and affects UDP-GlcNAc generation**

A. KEGG Classification for the differential metabolites of BMSCs from *Glul^Prx1^* and *Glul^fl/fl^* mice. B. Volcano plot for the differential metabolites of BMSCs from *Glul^Prx1^* and *Glul^fl/fl^* mice. C. KEGG Enrichment for the differential metabolites of BMSCs from *Glul^Prx1^* and *Glul^fl/fl^* mice. D. KEGG Enrichment for the differential proteins of BMSCs from *Glul^Prx1^* and *Glul^fl/fl^* mice. E. Western blot analysis of the expression of osteogenesis-related genes in BMSCs from *Glul^Prx1^* and *Glul^fl/fl^* mice after transfection with UAP1 plasmid. Abbreviations: ** represents P < 0.01 vs. other groups; * represents P < 0.05 vs. other groups.

**
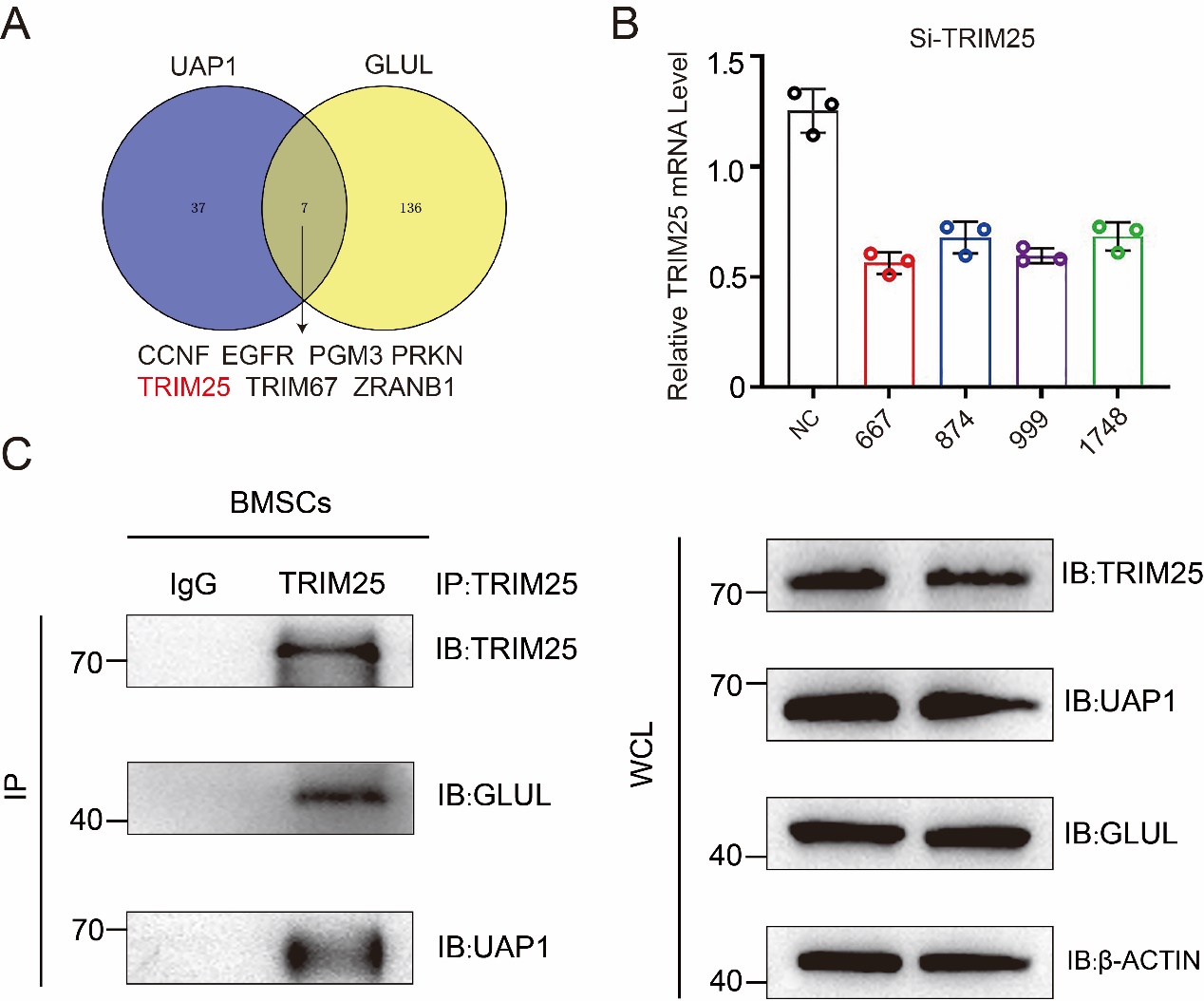
**

**Supplementary Fig. 4. GLUL regulates UAP1 expression by interacting with TRIM25**

A. The intersection result of proteins that interact with GLUL and proteins that interact with UAP1. B. Screening image of TRIM25 small interfering RNA sequence. C. Coimmunoprecipitation of TRIM25 with GLUL/UAP1 from BMSCs.


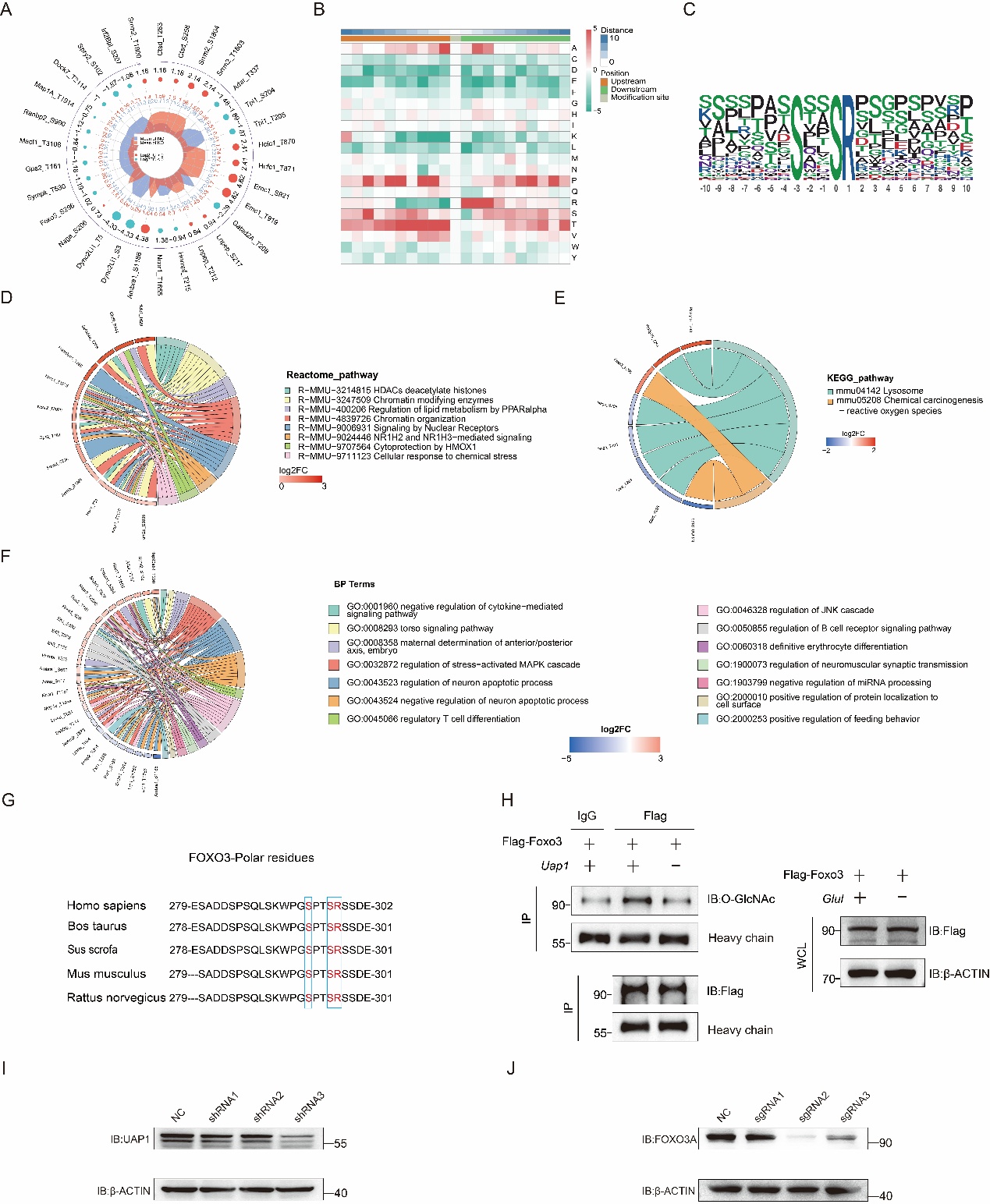


**Supplementary Fig. 5. O-GlcNAcylation of FOXO3-Ser296, which is regulated by GLUL, promotes the osteogenic differentiation of BMSCs**

A. Radar map of differential O-GlcNAcylation modification site in proteins. B. Heat map of the degree of change in frequency of amino acid occurrence near the modification site. C. Motif logo of xxxxxxxSxxSRxxxxxxxxx sequence. D. Up-Reactome pathway cirplot. E. Regulated-KEGG pathway cirplot. F. Regulated Biological Process cirplot. G. Comparison of the polar residue sequences of FOXO3 proteins from multiple species. H. The results of the co-immunoprecipitation experiment on the effect of *Uap1* knockdown on the level of O-GlcNAcylation modification of FOXO3 in BMSCs. I. Western blot image of *Uap1* knockdown lentivirus screening. J. Western blot image of *Foxo3* knockout lentivirus screening.

**Video Descriptions**

**Video 1** Representative video of bimolecular fluorescence complementation experiments between TRIM25 carrying YFP (aa 1--154) and GLUL carrying YFP (aa 155--238) in HEK-293T cells.

**Video 2** Representative video of bimolecular fluorescence complementation experiments between TRIM25 carrying YFP (aa 1--154) and UAP1 carrying YFP (aa 155--238) in HEK-293T cells.

**Video 3** Representative video of live-cell imaging after cotransfection of GFP-TRIM25, RFP-GLUL, and BFP-UAP1 into BMSC.

**Supplementary Tables 1 Motif analysis**

| **Motif Logo** | | | | **Motif** | **Motif Score** | **Foreground** | | **Background** | | **Fold Increase** |
| --- | --- | --- | --- | --- | --- | --- | --- | --- | --- | --- |
|  |  |  |  |  |  | **Matches** | **Size** | **Matches** | **Size** |  |
| 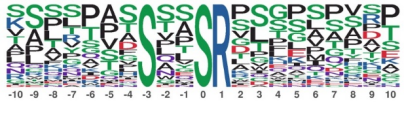 | | | | xxxxxxxSxx_S_Rxxxxxxxxx | 23.16 | 44 | 474 | 4254 | 776819 | 17.0 |
| 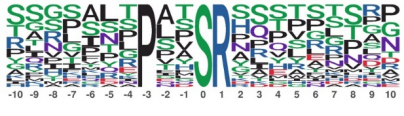 | | | | xxxxxxxPxx_S_Rxxxxxxxxx | 22.36 | 29 | 430 | 2772 | 772565 | 18.8 |
| 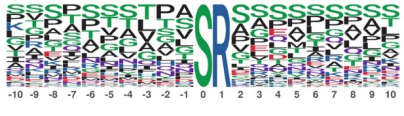 | | | | xxxxxxxxxx_S_Rxxxxxxxxx | 16.00 | 114 | 401 | 35335 | 769793 | 6.2 |
| 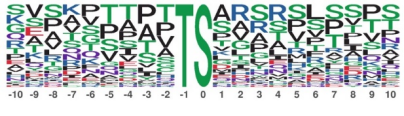 | | | | xxxxxxxxxT_S_xxxxxxxxxx | 14.88 | 55 | 287 | 41076 | 734458 | 3.4 |
| 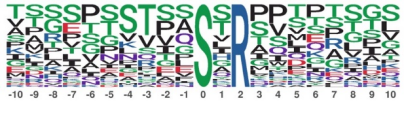 | | | | xxxxxxxxxx_S_xRxxxxxxxx | 10.90 | 40 | 232 | 35038 | 693382 | 3.4 |
| 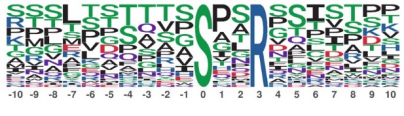 | | | | xxxxxxxxxx_S_xxRxxxxxxx | 12.77 | 39 | 192 | 33758 | 658344 | 4.0 |
| 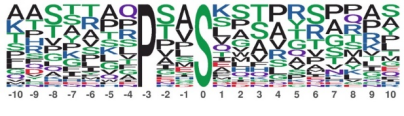 | | | | xxxxxxxPxx_S_xxxxxxxxxx | 8.69 | 33 | 153 | 41849 | 624586 | 3.2 |
| 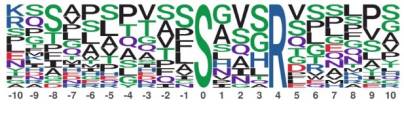 | | | | xxxxxxxxxx_S_xxxRxxxxxx | 6.05 | 21 | 120 | 30053 | 582737 | 3.4 |
| 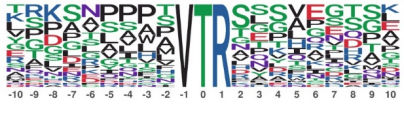 | | | | xxxxxxxxxV_T_Rxxxxxxxxx | 25.26 | 38 | 544 | 1667 | 494714 | 20.7 |
| 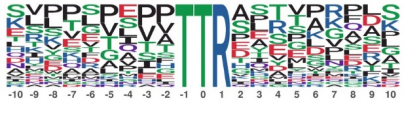 | | | | xxxxxxxxxT_T_Rxxxxxxxxx | 26.88 | 31 | 506 | 1200 | 493047 | 25.2 |
| 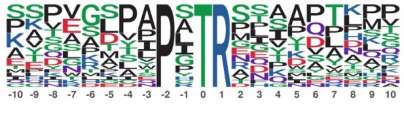 | | | | xxxxxxxxPx_T_Rxxxxxxxxx | 26.88 | 23 | 475 | 1340 | 491847 | 17.8 |
| 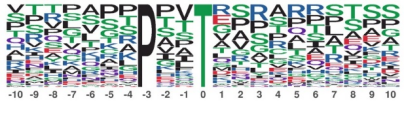 | | | | xxxxxxxPxx_T_xxxxxxxxxx | 16.00 | 86 | 452 | 32656 | 490507 | 2.9 |
| 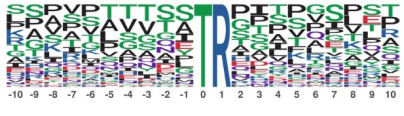 | | | | xxxxxxxxxx_T_Rxxxxxxxxx | 16.00 | 68 | 366 | 17421 | 457851 | 4.9 |
| 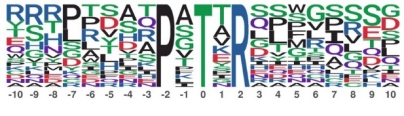 | | | | xxxxxxxxPx_T_xRxxxxxxxx | 25.97 | 20 | 298 | 1538 | 440430 | 19.2 |
| 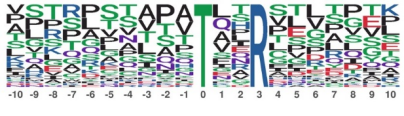 | | | | xxxxxxxxxx_T_xxRxxxxxxx | 10.15 | 43 | 278 | 22056 | 438892 | 3.1 |
| 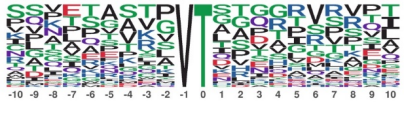 | | | | xxxxxxxxxV_T_xxxxxxxxxx | 7.13 | 42 | 235 | 30511 | 416836 | 2.4 |
| 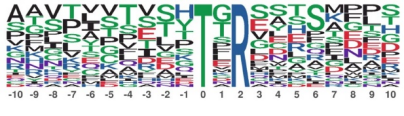 | | | | xxxxxxxxxx_T_xRxxxxxxxx | 6.82 | 28 | 193 | 18262 | 386325 | 3.1 |
| 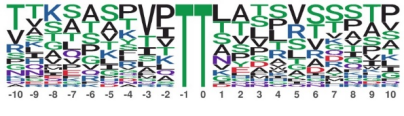 | | | | xxxxxxxxxT_T_xxxxxxxxxx | 6.70 | 29 | 165 | 22127 | 368063 | 2.9 |
| 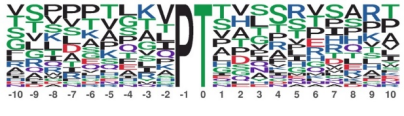 | | | | xxxxxxxxxP_T_xxxxxxxxxx | 7.89 | 29 | 136 | 22502 | 345936 | 3.3 |
|  |  |  |  |  |  |  |  |  |  |  |

**Supplementary Tables 2 List of primary antibodies**

| ***Antibody*** | ***Catalog Number*** | ***Manufacturer*** | ***RRID*** |
| --- | --- | --- | --- |
| *Anti-Glutamine Synthetase antibody* | *ab176562* | *abcam* | *AB****_****2868472* |
| *Glsyn mouse mAb Alexa Fluor647* | *sc-74430* | *Santa Cruz* | *AB_1127501* |
| *Anti-Runx2 antibody* | *ab236639* | *abcam* | *AB****_****2937078* |
| *Anti-Sp7/Osterix antibody* | *ab209484* | *abcam* | *AB_2892207* |
| *Anti-Osteopontin antibody* | *ab283656* | *abcam* | *AB****_****2894861* |
| *Anti-Ubiquitin (linkage-specific K48)*  *antibody* | *ab140601* | *abcam* | *AB_2783797* |
| *Anti-UAP1 antibody*  *UAP1 Monoclonal antibody* | *ab95949*  *67545-1-IG* | *Abcam*  *proteintech* | *AB_10681040*  *AB_2882762* |
| *Anti-TRIM25/EFP antibody*  *EFP antibody (E-4)* | *ab167154*  *sc-166926* | *Abcam*  *Santa Cruz* | *AB_2721902*  *AB_10608081* |
| *O-GlcNAc Monoclonal Antibody (RL2)* | *MA1-072* | *ThermoFisher* | *AB_326364* |
| *Mouse anti DDDDK-Tag mAb* | *AE005* | *abclonal* | *AB_2770401* |
| *Mouse anti Myc-Tag mAb* | *AE010* | *abclonal* | *AB_2770408* |
| *Mouse anti HA-Tag mAb* | *AE008* | *abclonal* | *AB_2770404* |
| *Mouse anti V5-Tag mAb* | *AE017* | *abclonal* | *AB_2770413* |
| *Rabbit anti DDDDK-Tag pAb* | *AE004* | *abclonal* | *AB_2771921* |
| *Ubiquitin Rabbit mAb* | *A19686* | *abclonal* | *AB_2862735* |
| *FOXO3A Rabbit mAb* | *A9270* | *abclonal* | *AB_2863703* |
| *β-Actin Rabbit mAb* | *AC026* | *abclonal* | *AB_2768234* |
| *Anti-OGT Antibody* | *11576-2-ap* | *proteintech* | *AB_2156943* |
| *Anti-HA-Tag Antibody* | *51064-2-ap* | *proteintech* | *AB_11042321* |
| *Anti-MYC-Tag Antibody* | *16286-1-ap* | *proteintech* | *AB_11182162* |
| *V5-tag Polyclonal Antibody* | *14440-1-ap* | *proteintech* | *AB_2878059* |
| *Beta Tubulin Polyclonal antibody* | *10068-1-ap* | *proteintech* | *AB_2303998* |
| *Rabbit Anti-Collagen I antibody* | *bs-7158R* | *Bioss* | *AB_3674358* |

**Supplementary Tables 3 List of secondary antibodies**

| ***Antibody*** | ***Catalog Number*** | ***Manufacturer*** | ***RRID*** |
| --- | --- | --- | --- |
| *Mouse Anti-rabbit IgG (Conformation Specific) (L27A9) mAb (HRP Conjugate)* | *5127* | *Cell Signaling Technology* | *AB_10892860* |
| *Dylight 549, Goat Anti-Mouse IgG* | *A23310* | *Abbkine* | *AB_2813874* |
| *Dylight 488, Goat Anti-Rabbit IgG* | *A23220* | *Abbkine* | *AB_2737289* |
| *Cy3, Goat Anti-Mouse IgG* | *A22210* | *Abbkine* | *AB_2923040* |
| *HRP-labeled Goat Anti-Mouse IgG(H+L)* | *A0216* | *Beyotime* | *AB_2860575* |
| *HRP-labeled Goat Anti-Rabbit IgG(H+L)* | *A0208* | *Beyotime* | *AB_2892644* |

**Supplementary Tables 4 Nucleotide sequences of q-PCR primers**

| ***Names*** | ***Species*** | ***Sequence (5′ to 3′)*** |
| --- | --- | --- |
| *TRIM25* | *Homo sapiens* | Sense: CCAGTCTACATCCCCGAGGT  Antisense: GGATTTGTGTGTGGACGCTG |
| *β-ACTIN* | *Homo sapiens* | Sense: GAAGAGCTACGAGCTGCCTGA  Antisense: CAGACAGCACTGTGTTGGCG |
| *GLUL* | *Homo sapiens* | Sense: GTGAGAAAGTCCAGGCCATGTAT  Antisense: CTGTTGGAACCCTCAGACTGTAA |

**Supplementary Tables 5 Sequences of siRNA**

| ***Names*** | ***Species*** | ***Sequence (5′ to 3′)*** |
| --- | --- | --- |
| *SiTRIM25* | *Homo sapiens* | S: GGCACAAACUAACUGUCAUTT  AS: AUGACAGUUAGUUUGUGCCTT |

**Supplementary Tables 6 Primers for Mouse Gene Identification**

| ***Names*** | ***Species*** | ***Sequence (5′ to 3′)*** |
| --- | --- | --- |
| *Glul* | *Mus musculus* | Sense: CTAGAAGCAGTTGTCCTGTGAATC  Antisense: TATAATTAAAGTCAGCCACGCAGC |
| *Prx1* | *Mus musculus* | Sense: GCTCTGATGTTGGCAAAGGGGT  Antisense: AACATCTTCAGGTTCTGCGGG |
