# Supplementary material for: GLUL mediates FOXO3 O-GlcNAcylation to regulate the osteogenic differentiation of BMSCs and senile osteoporosis
Source: Cell Death Differ. 2025 Jul 11;32(12):2399–411. doi: 10.1038/s41418-025-01543-2 (PMC12669651; doi:10.1038/s41418-025-01543-2)

Fig_1B

IB:GLUL

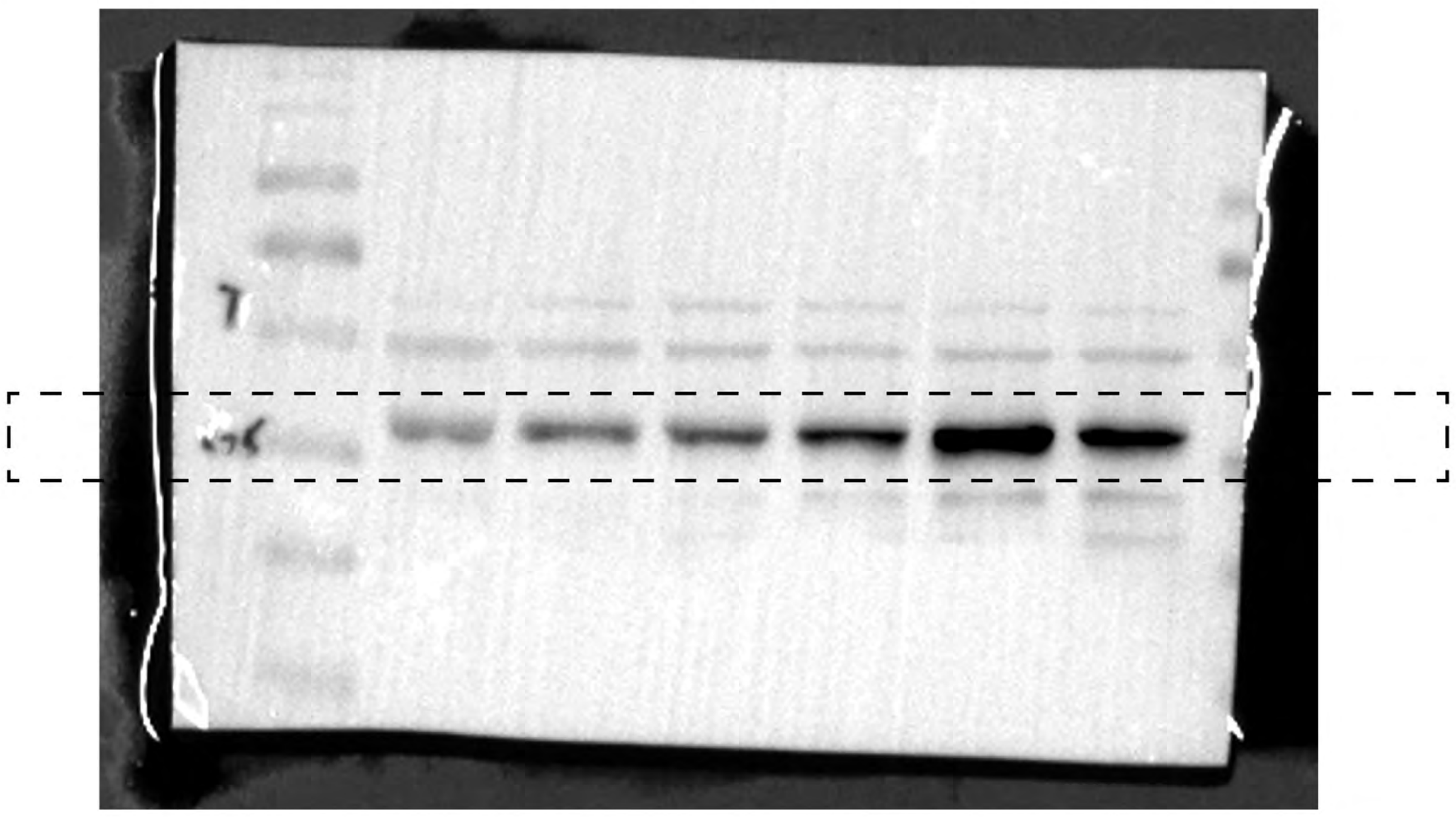

IB:β-TUBULIN

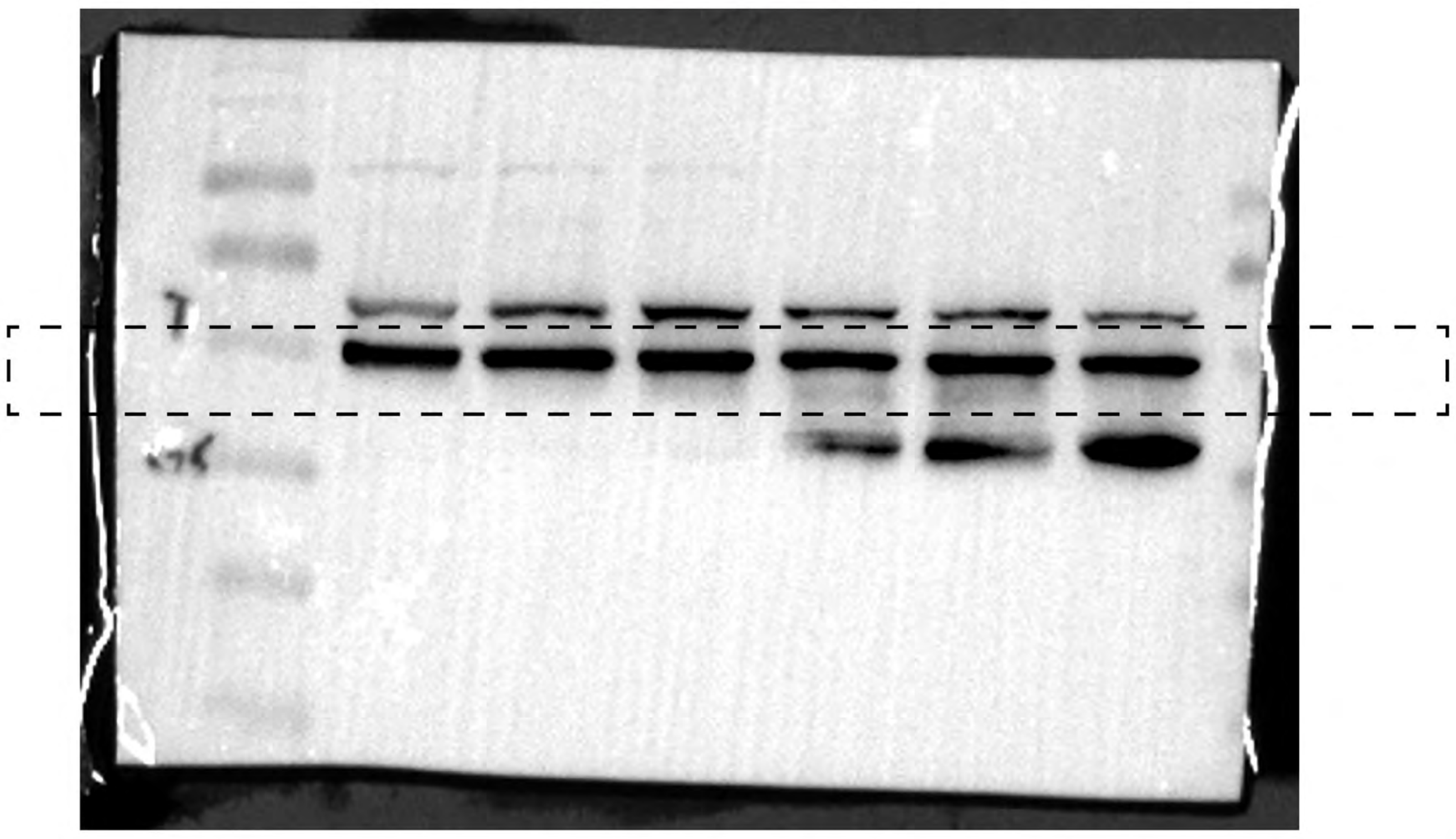

Fig_1G

IB:GLUL

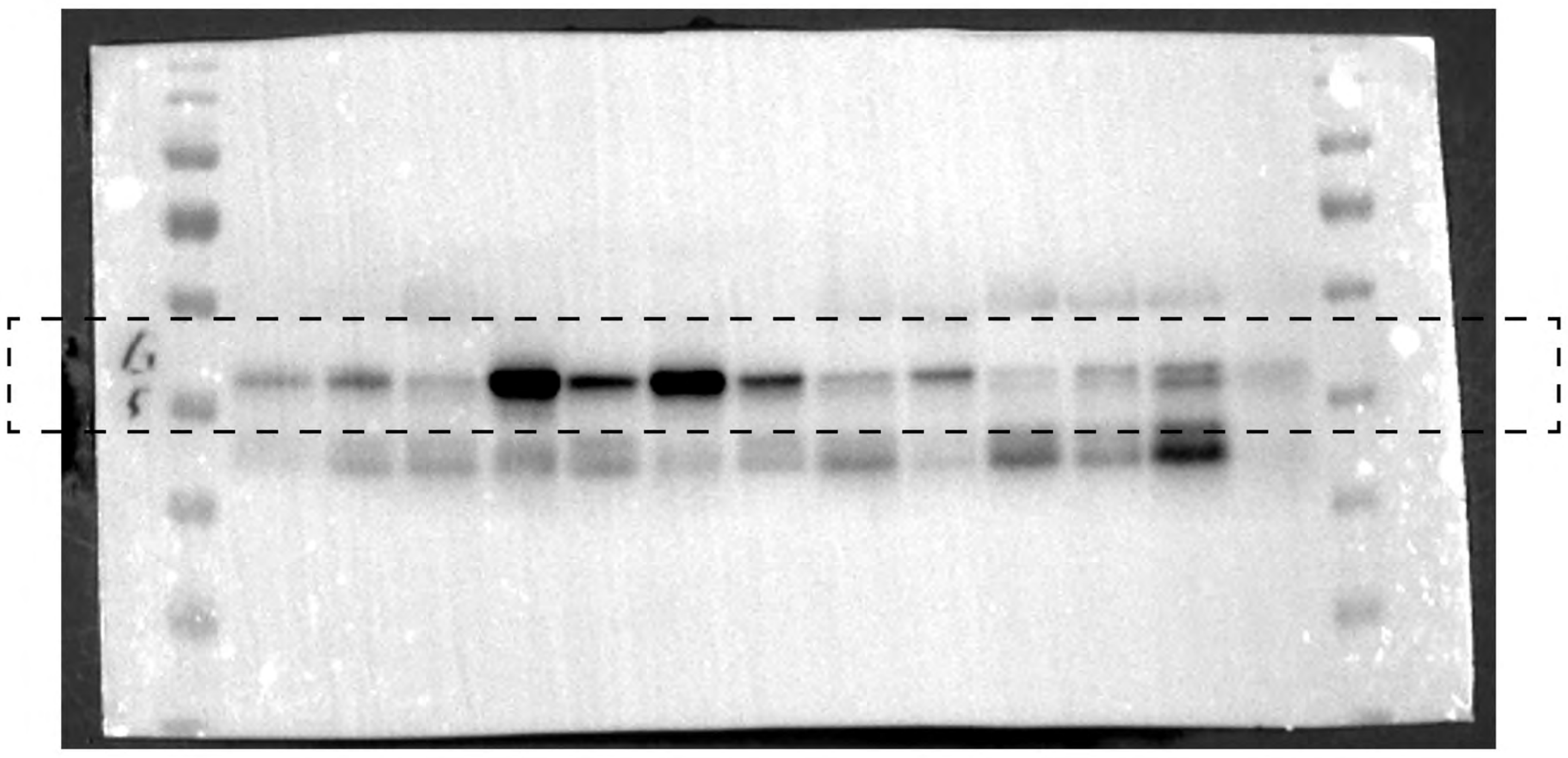

IB:β-TUBULIN

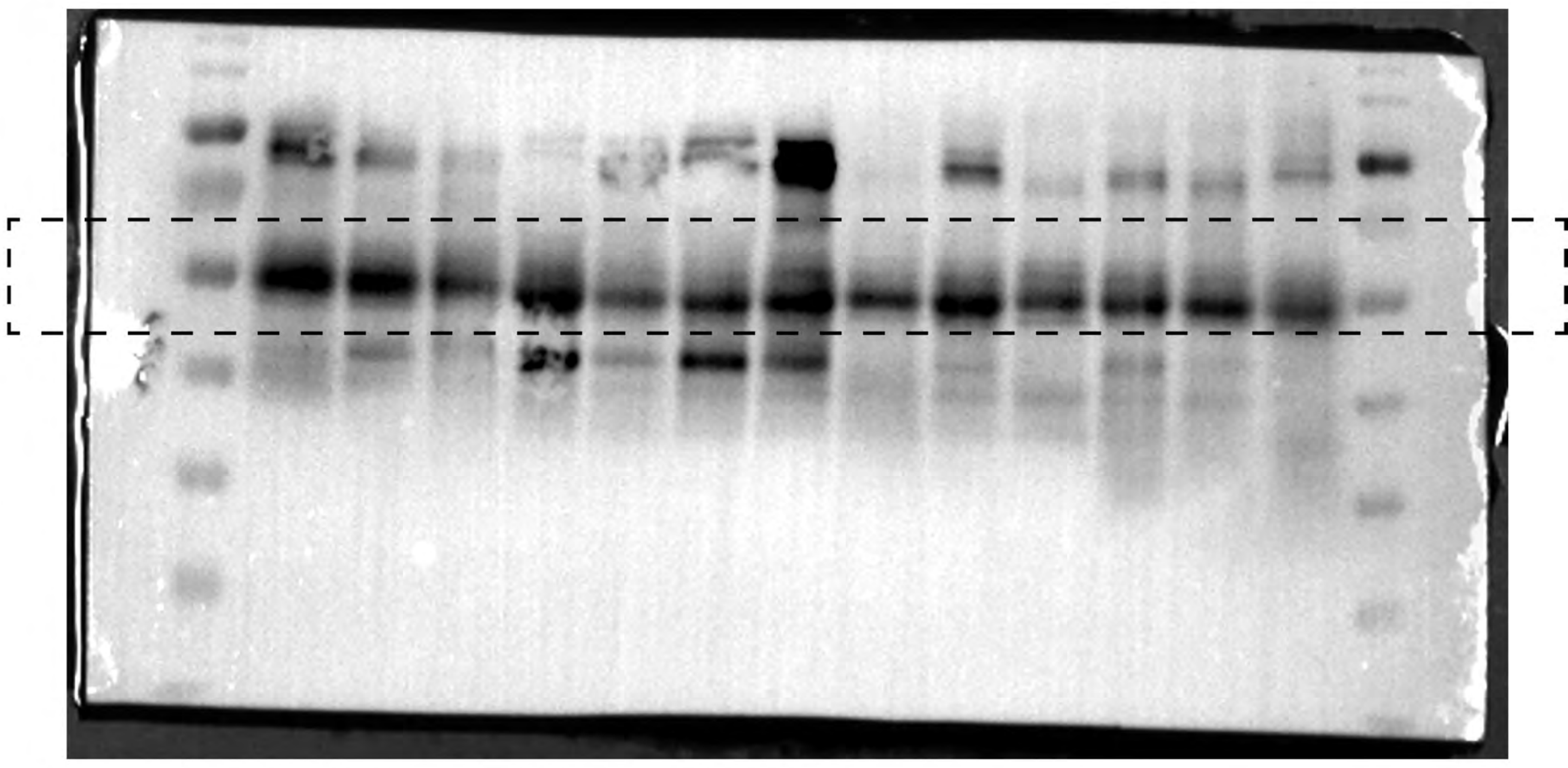

Supplemental Fig_1C

IB:GLUL

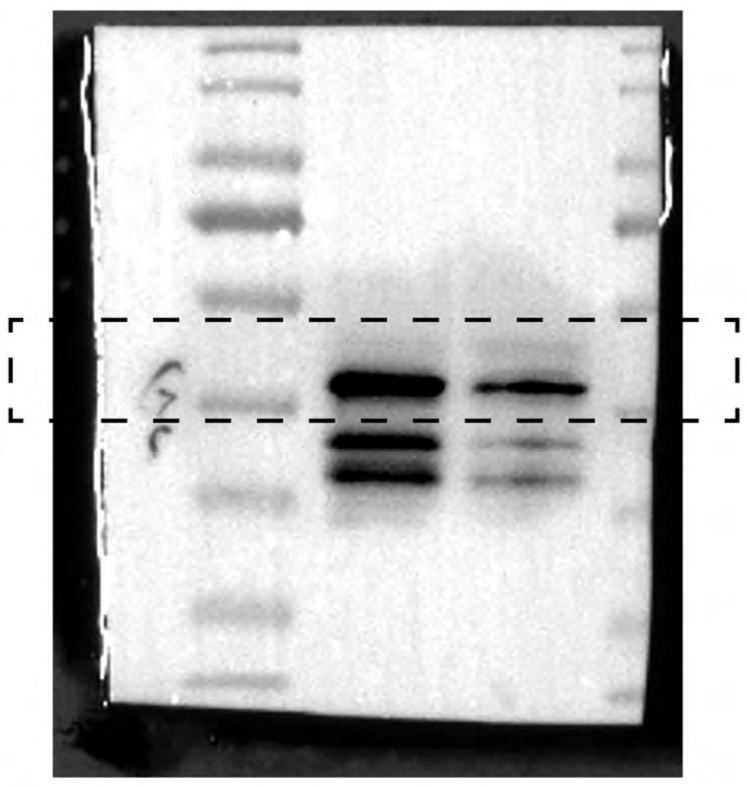

IB:β-TUBULIN

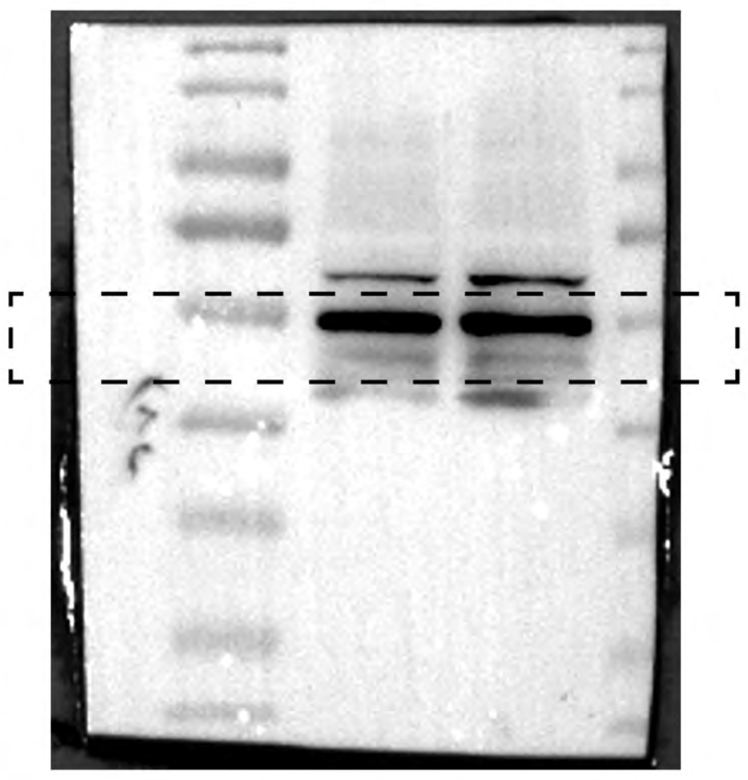

Supplemental Fig_2B

IB:GLUL

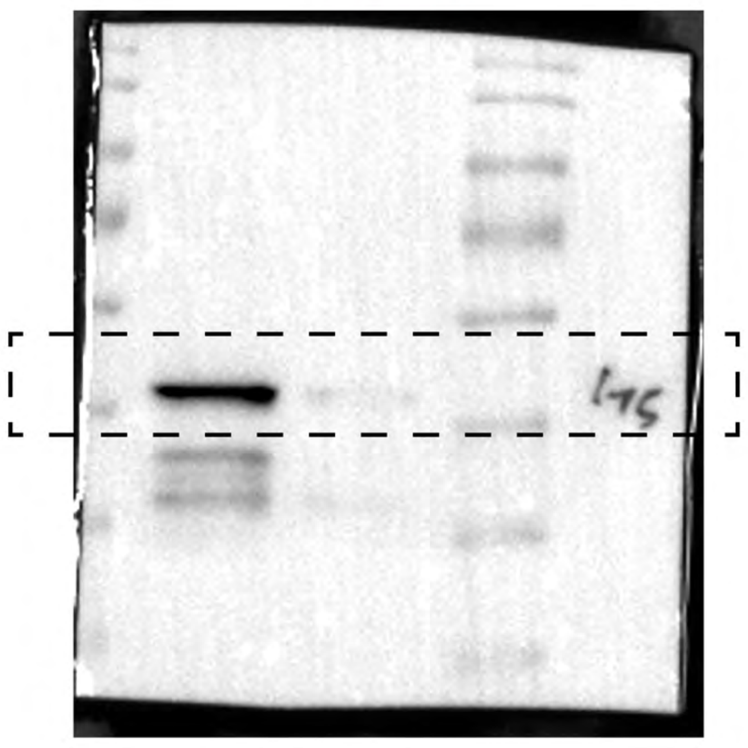

IB:β-TUBULIN

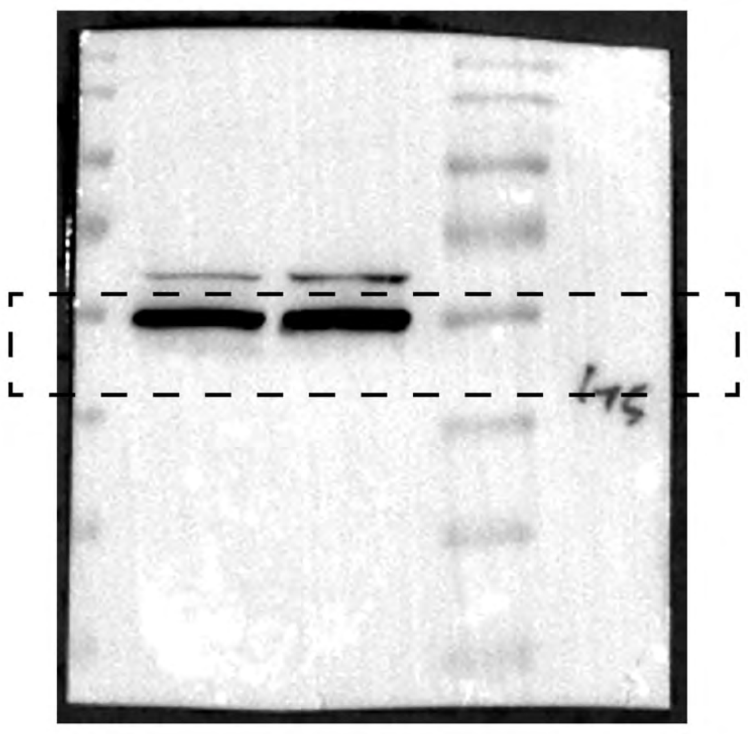

Supplemental Fig_2C

IB:RUNX2

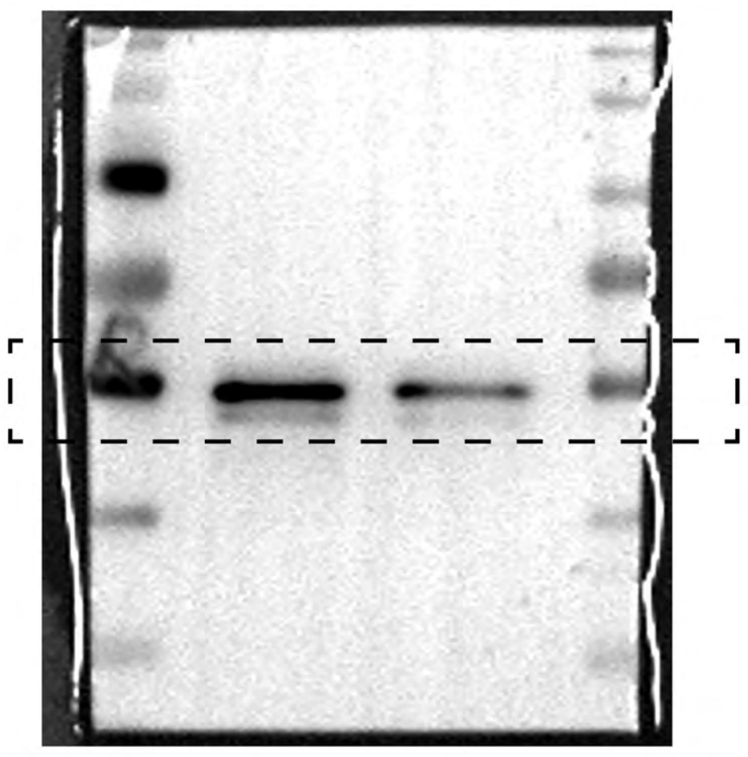

IB:β-ACTIN

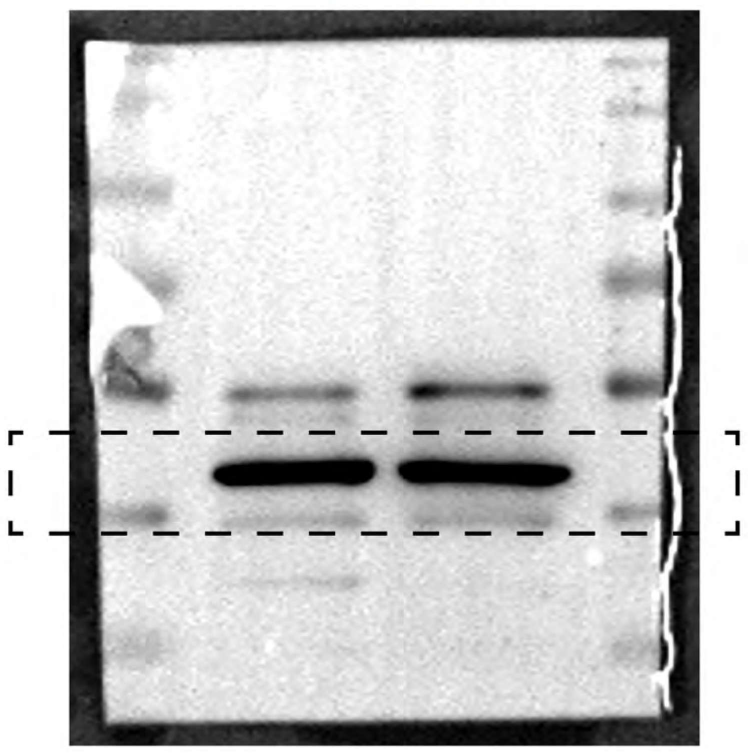

IB:OSX

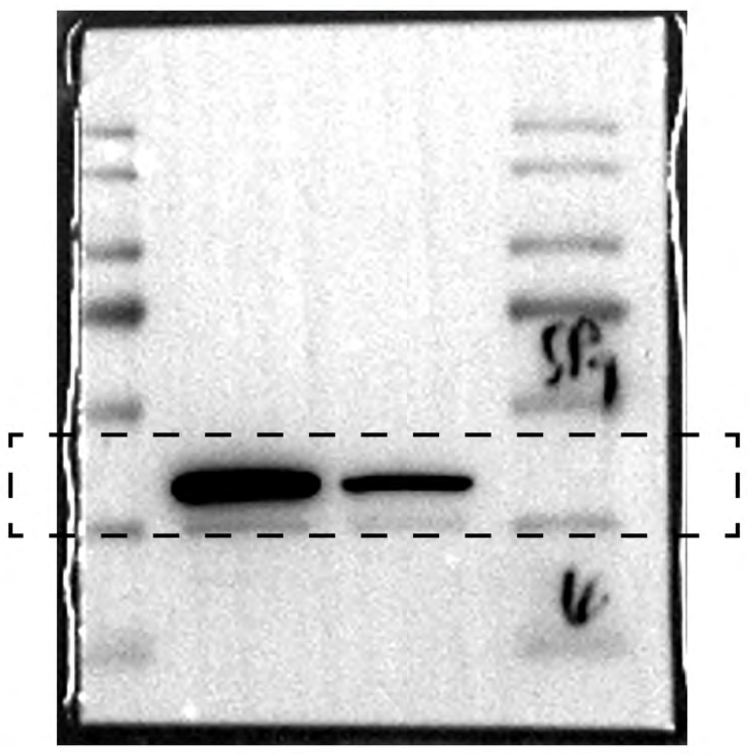

IB:β-TUBULIN

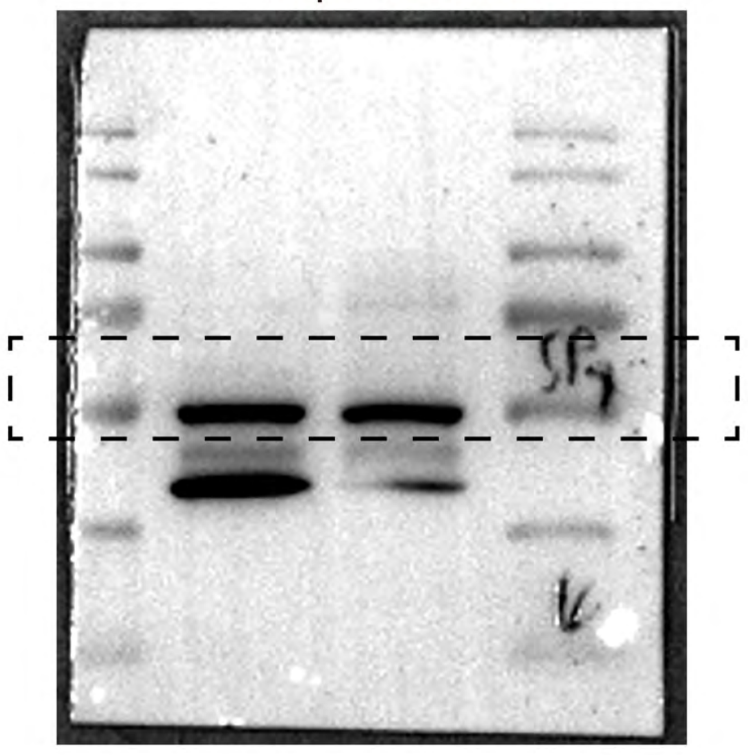

Supplemental Fig_2C

IB:COL1A1

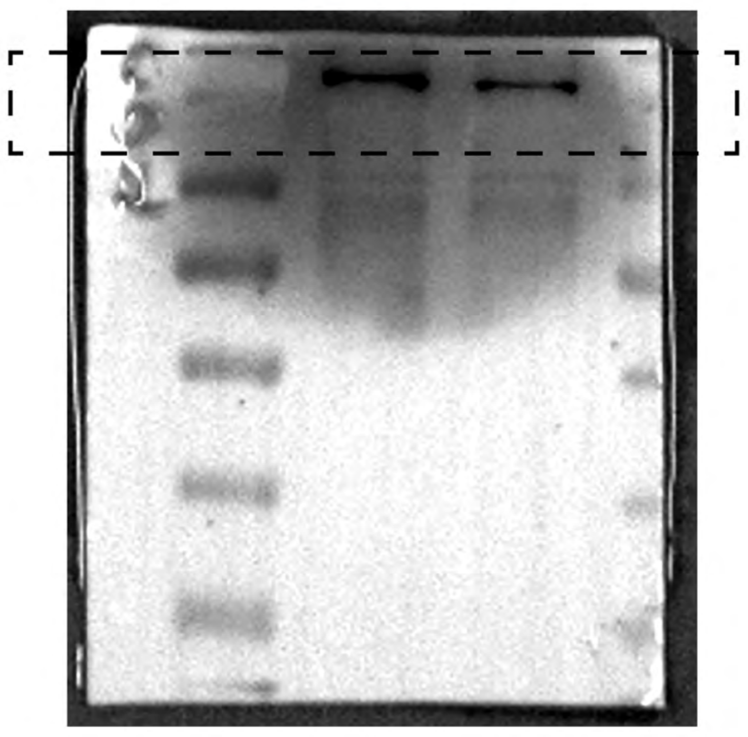

IB:β-ACTIN

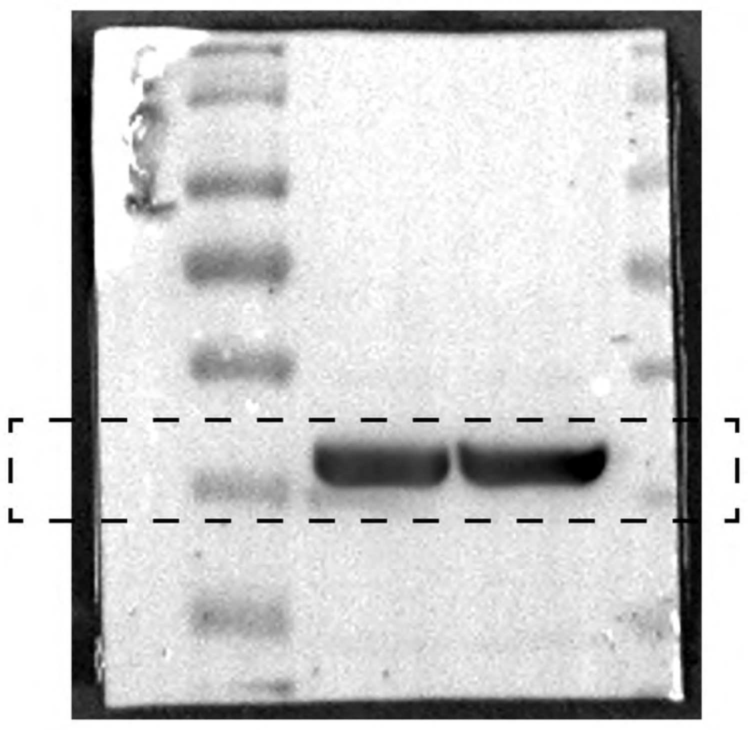

IB:SPP1

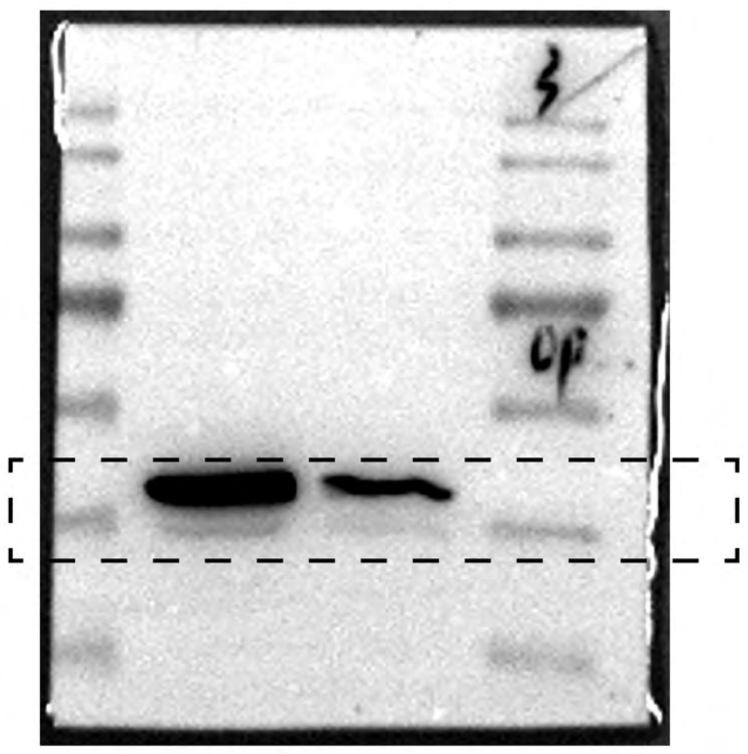

IB:β-TUBULIN

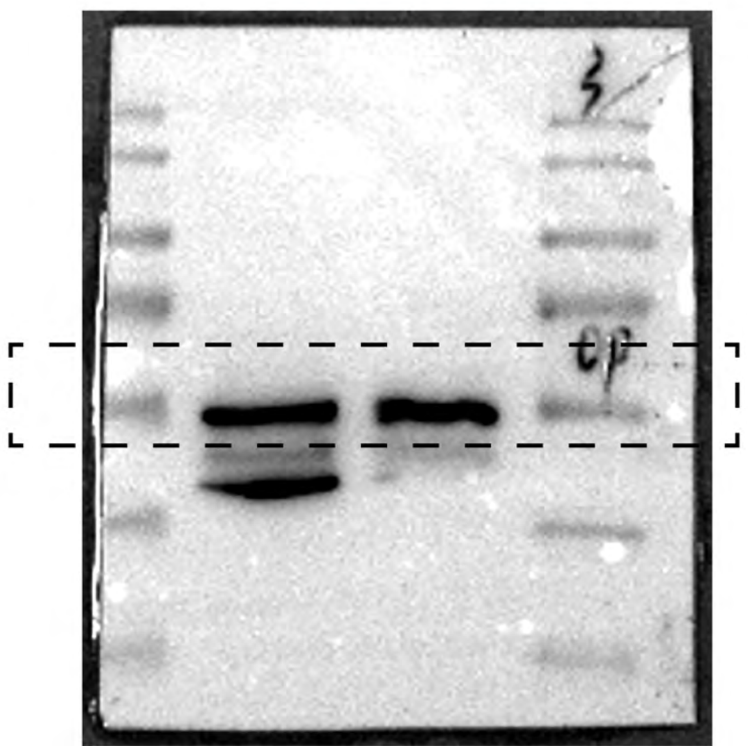

Fig_3D

IB:O-GlcNAc

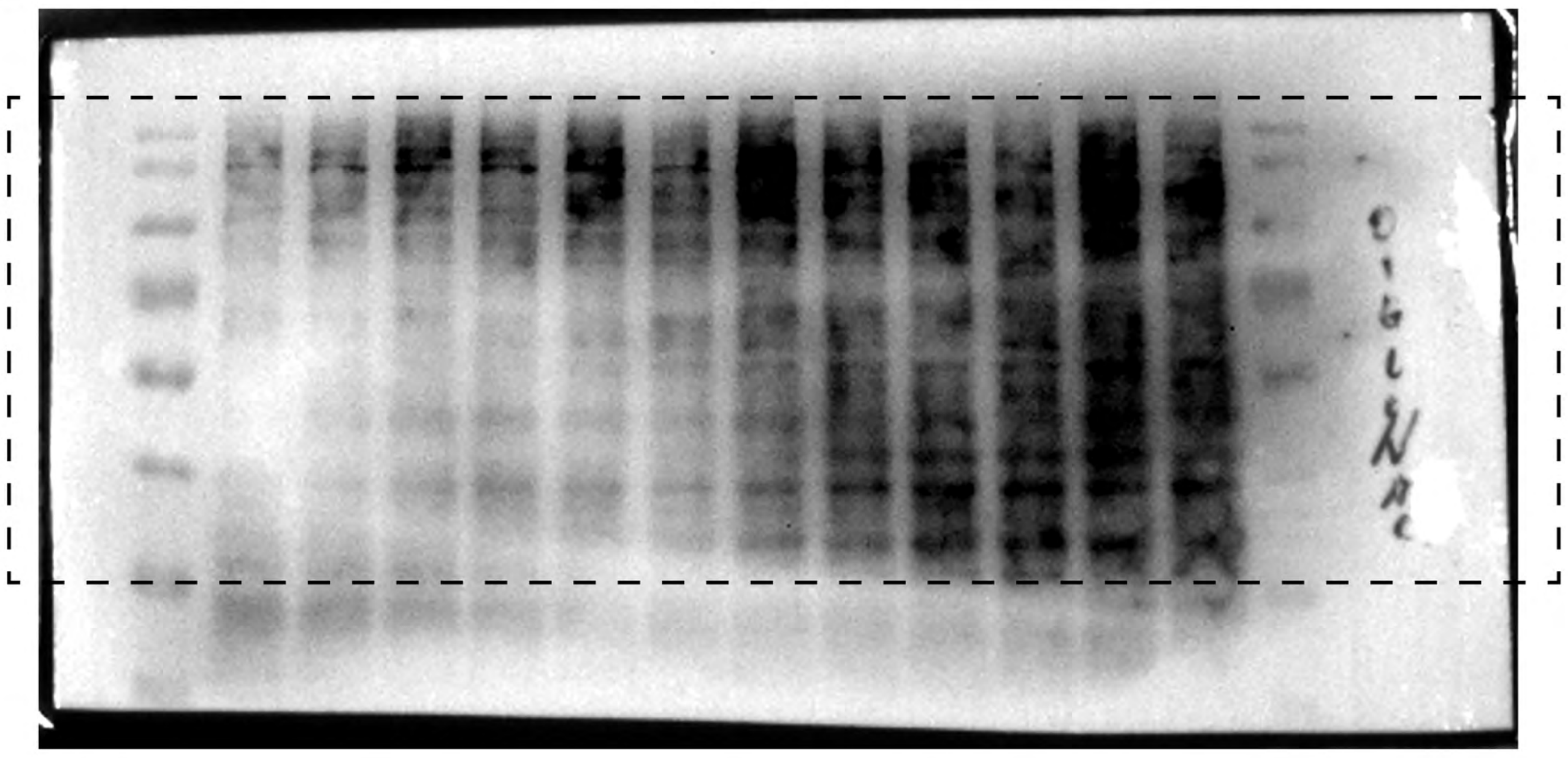

IB:β-ACTIN

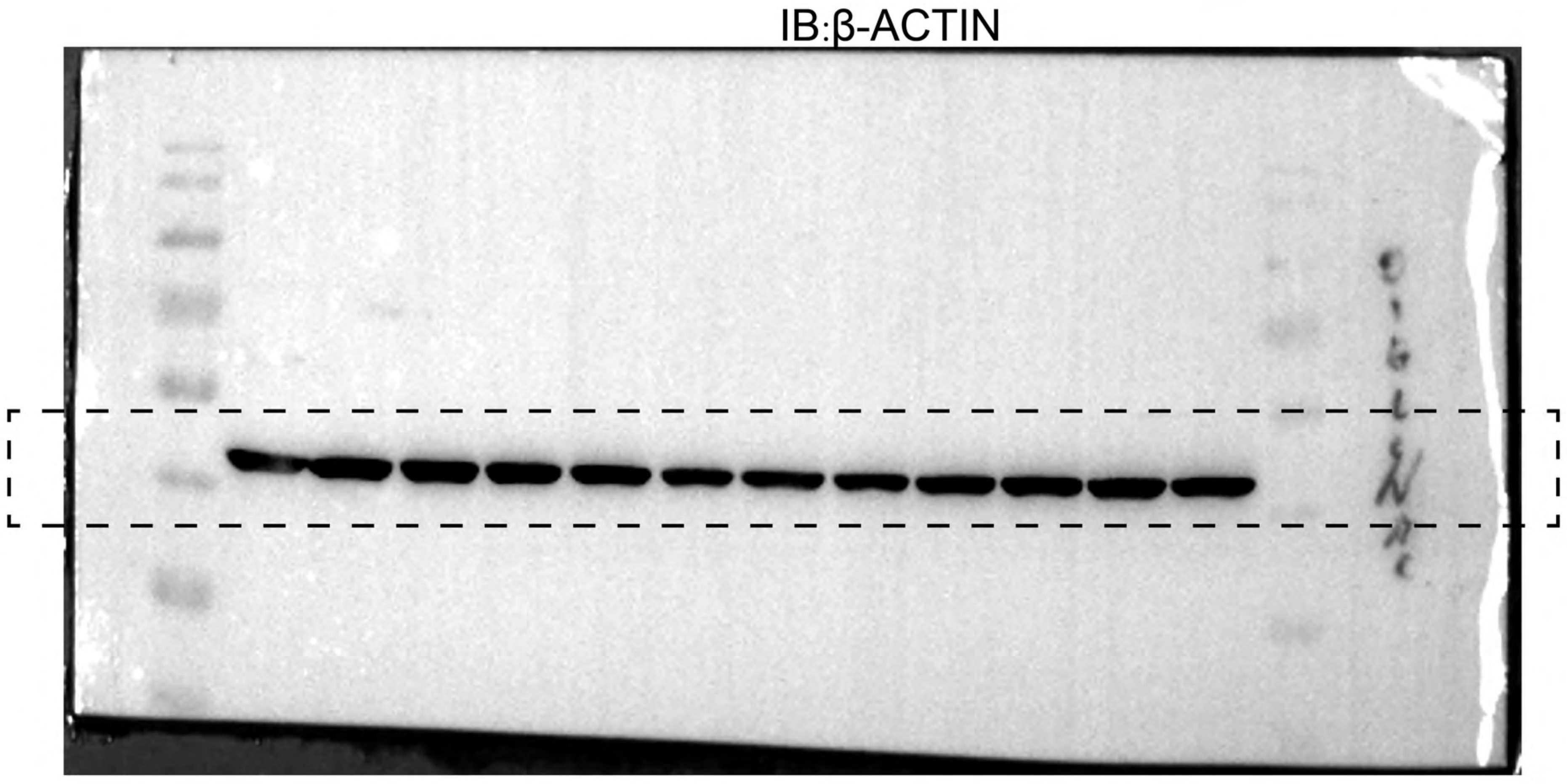

Fig_3E

IB:UAP1

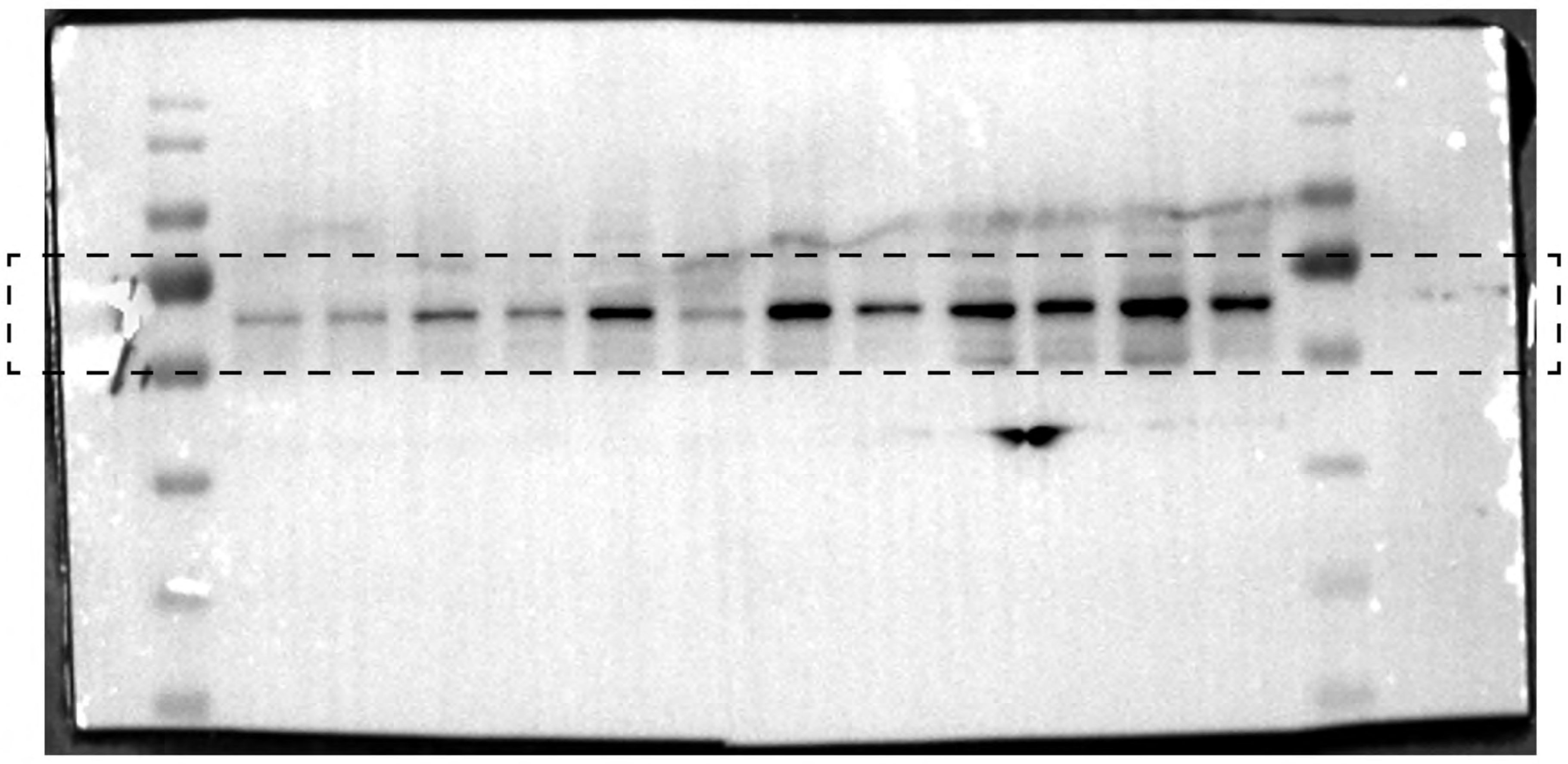

IB:β-ACTIN

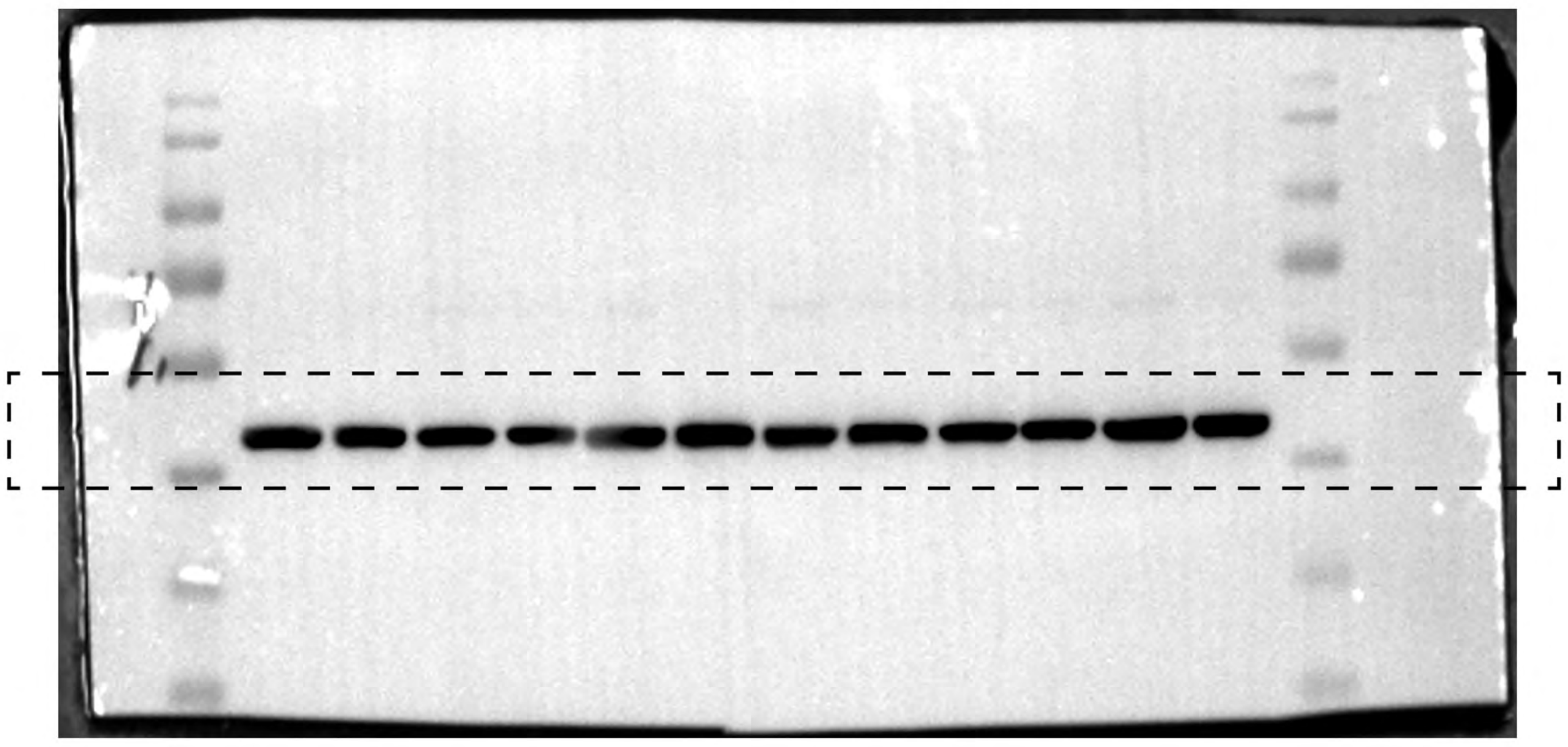

Fig_3F

IB:UAP1

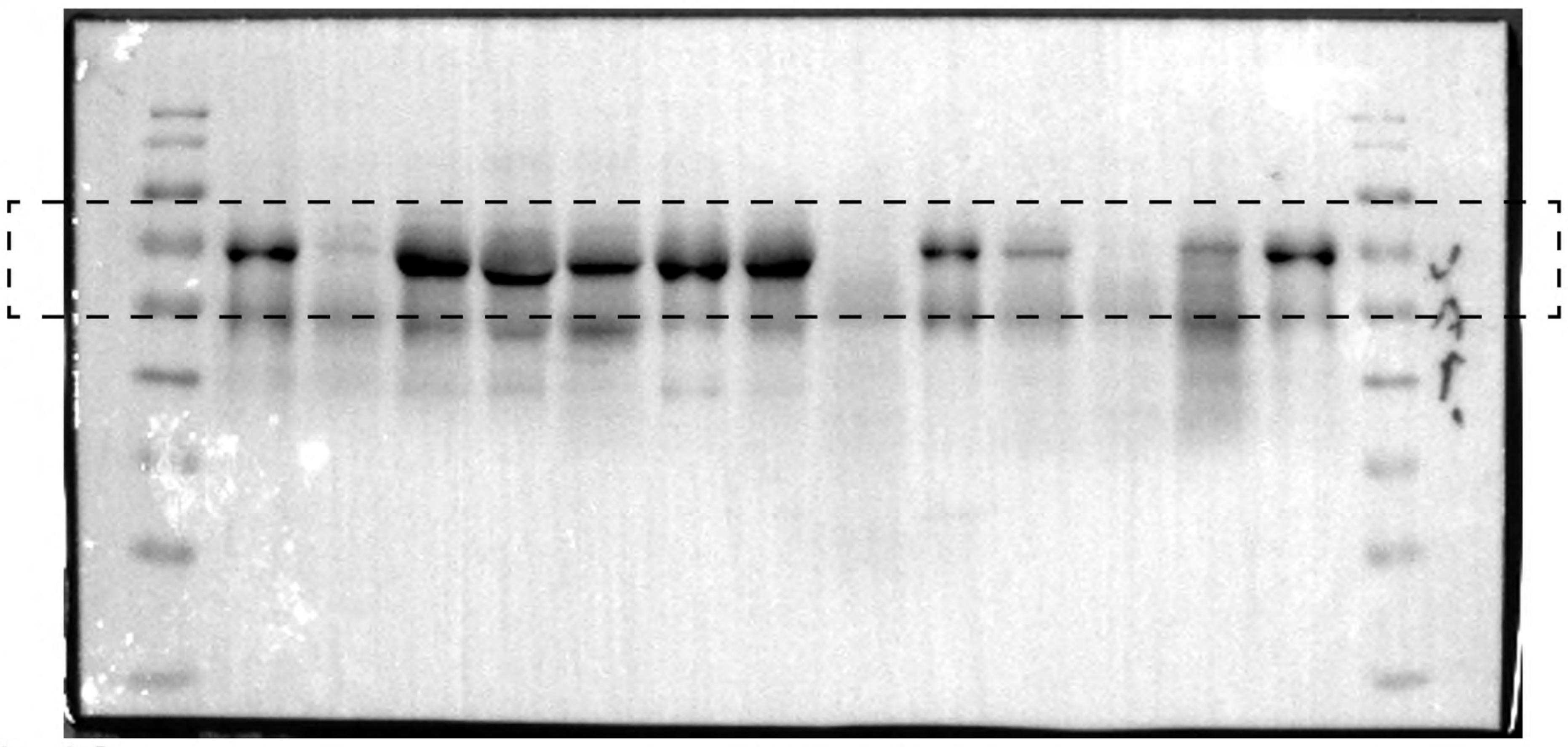

IB:β-TUBULIN

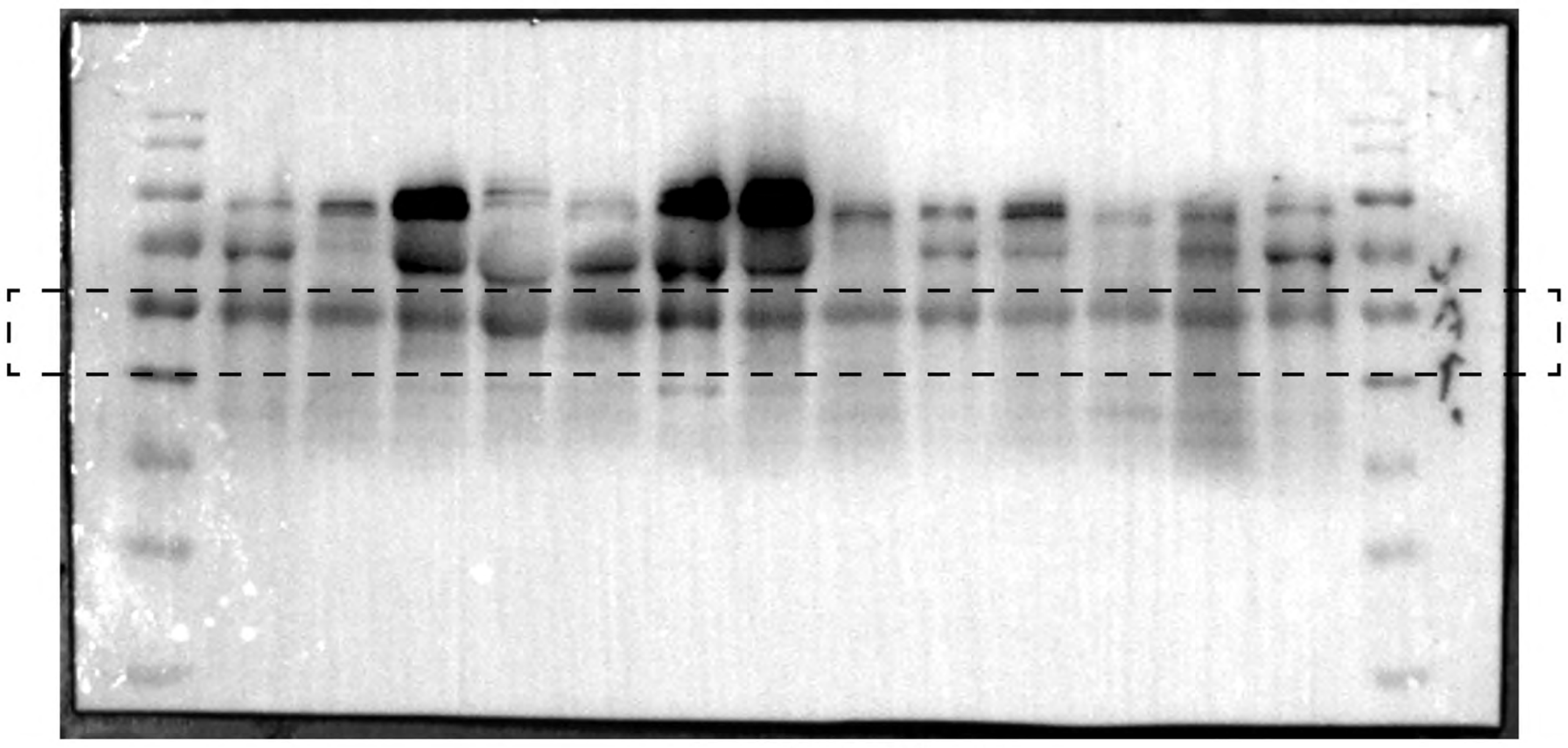

Fig_3G

IB:OGT

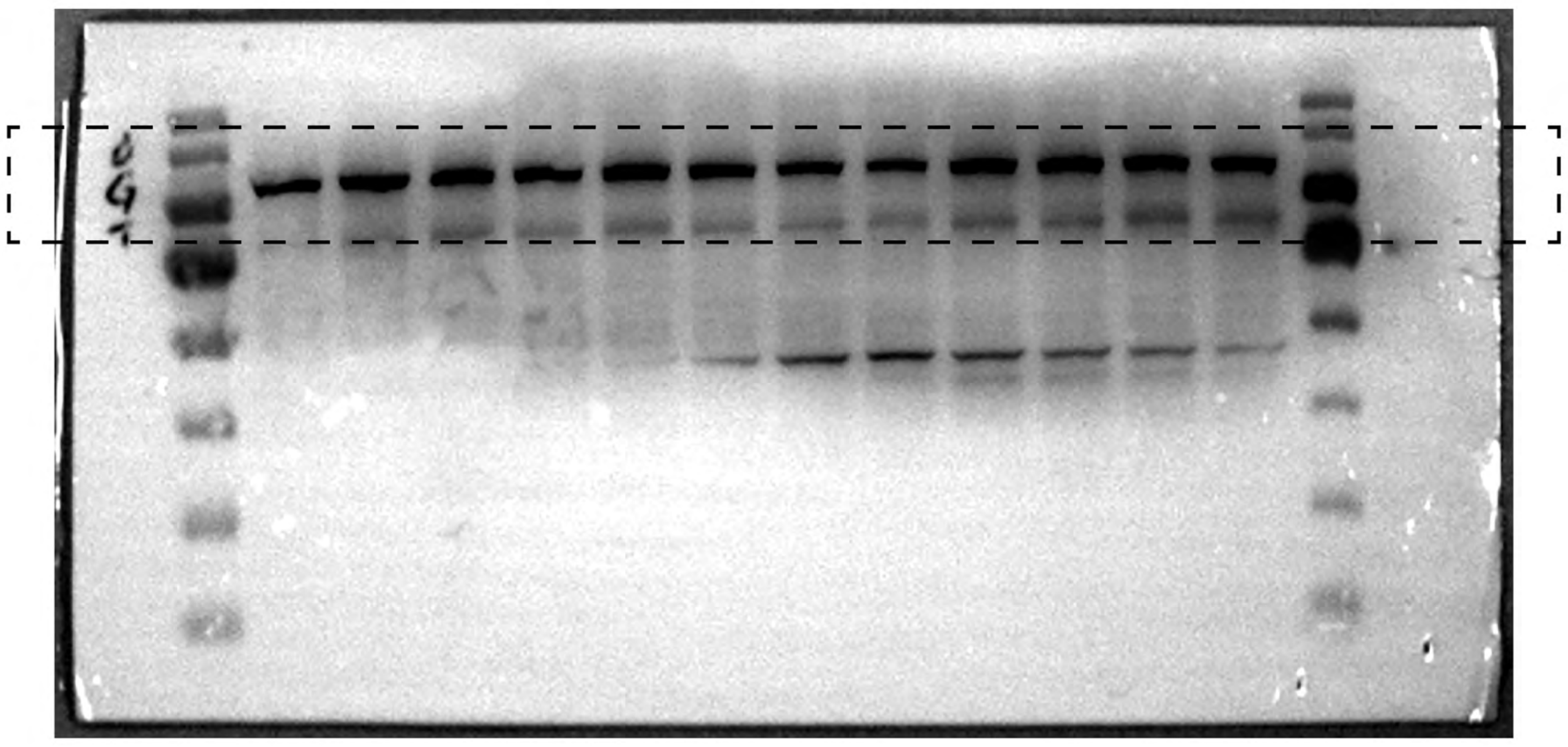

IB:β-ACTIN

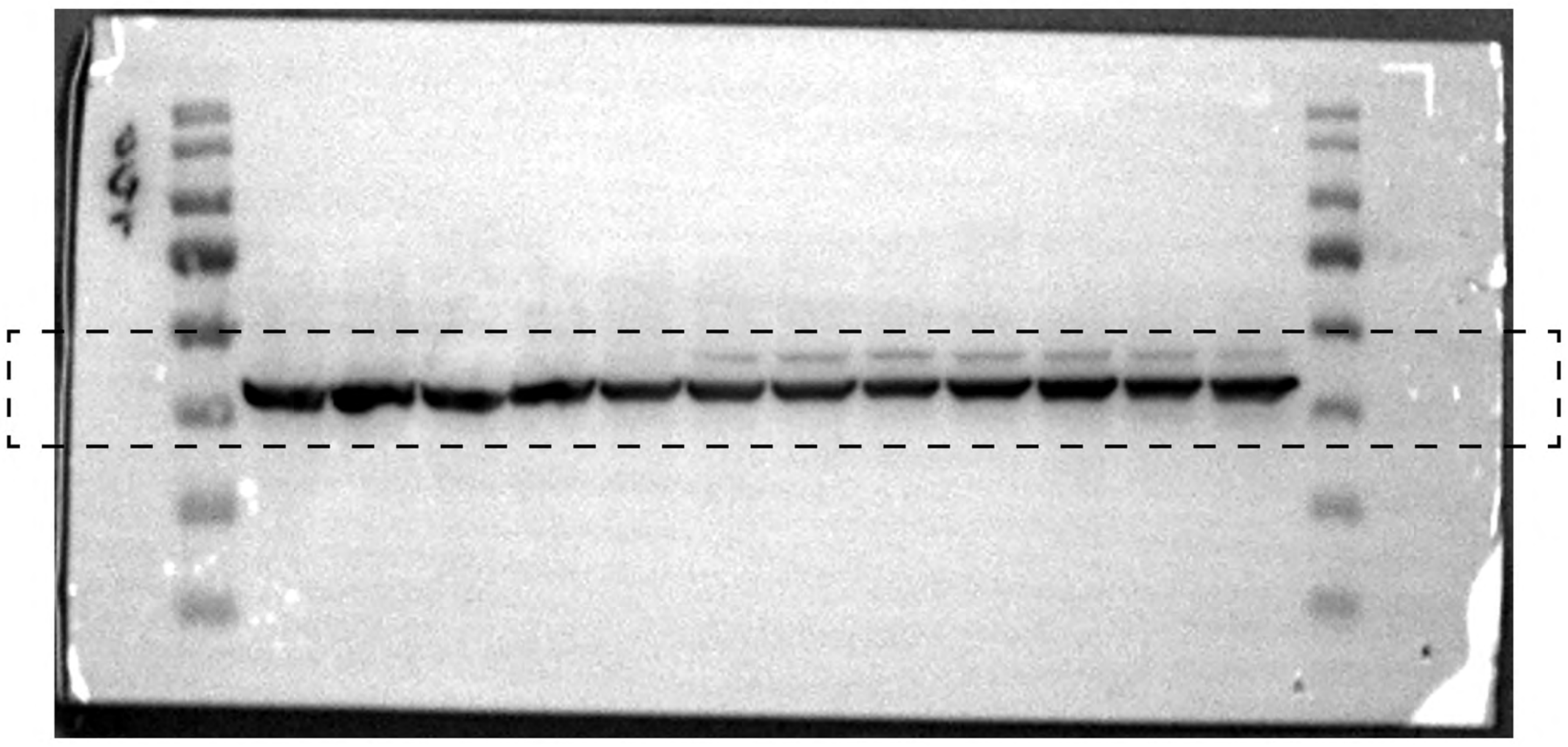

Supplemental Fig_3E

IB:RUNX2

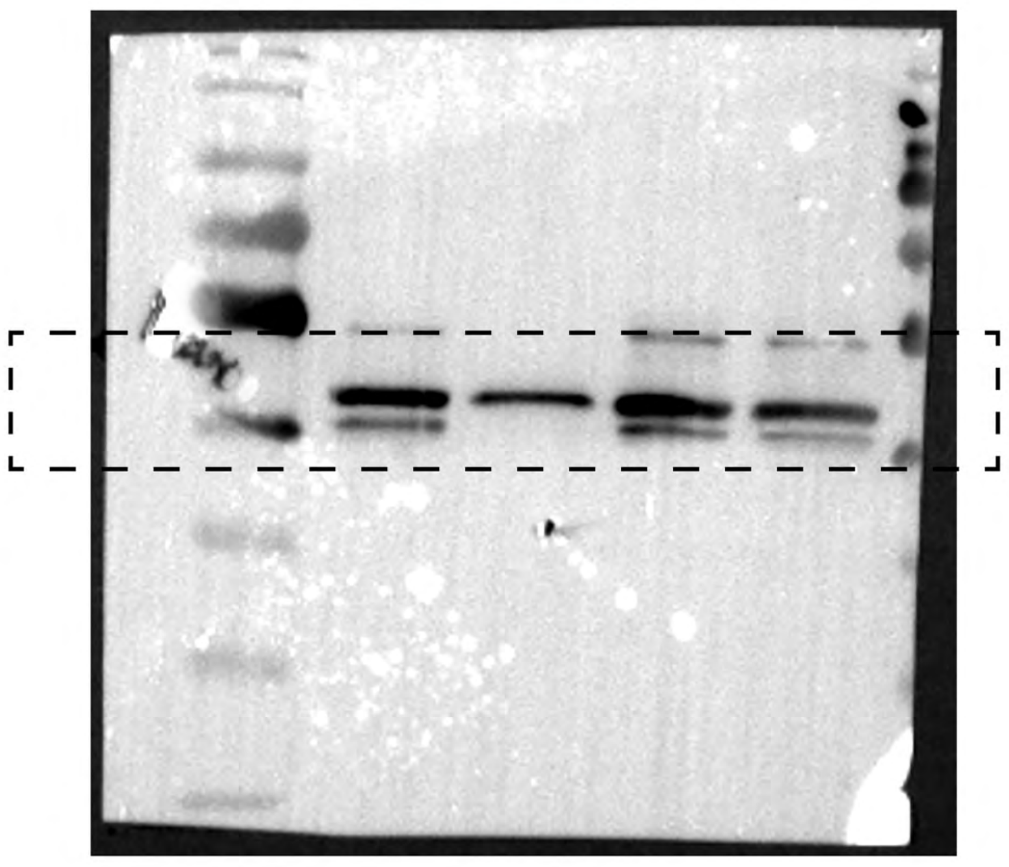

IB:β-ACTIN

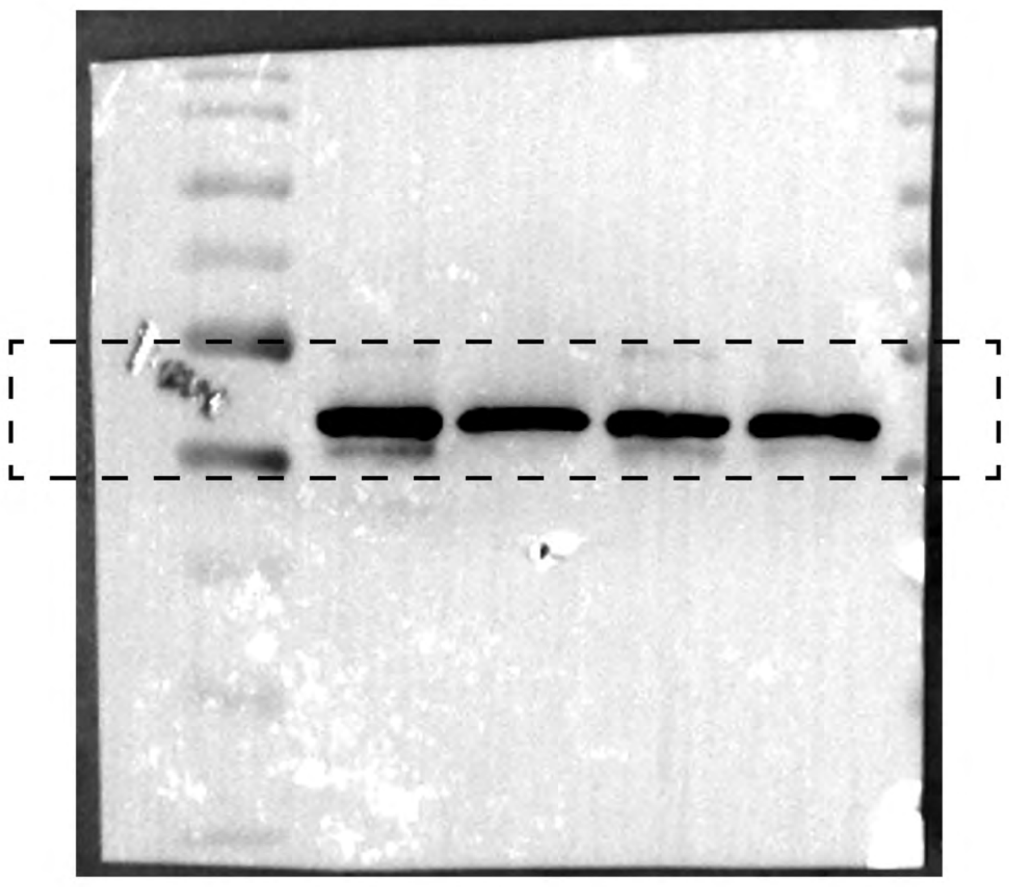

IB:SPP1

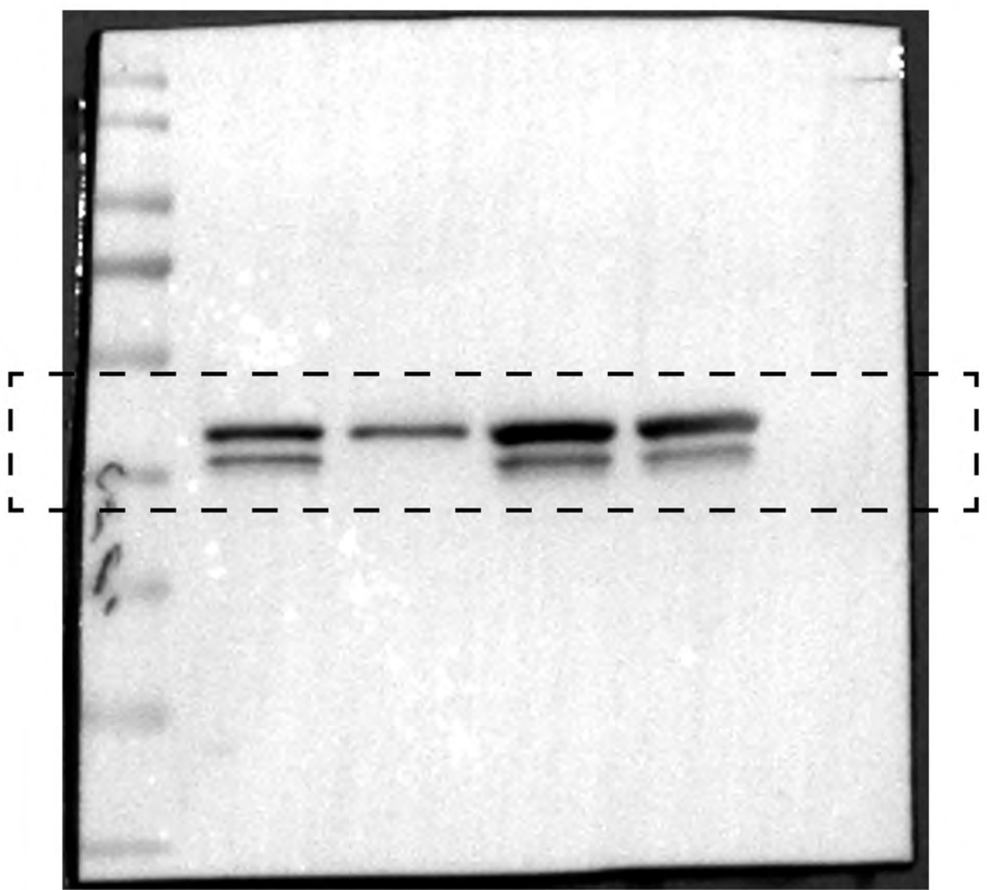

IB:β-ACTIN

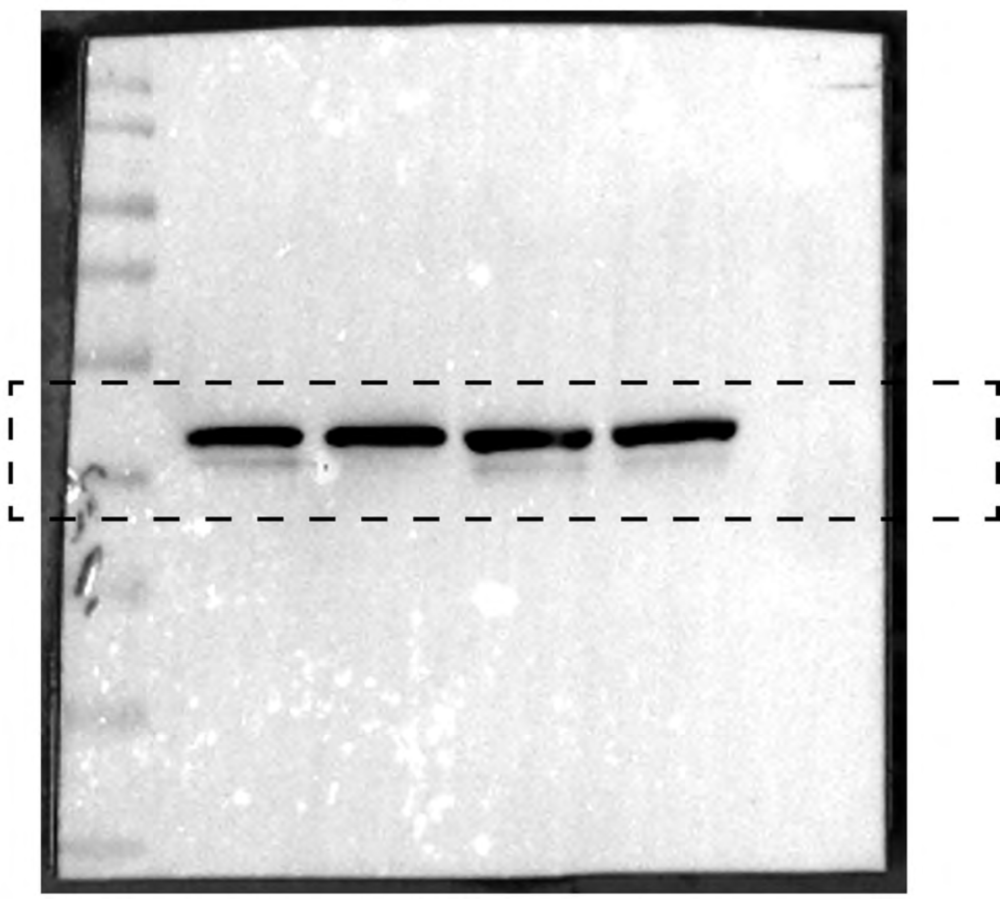

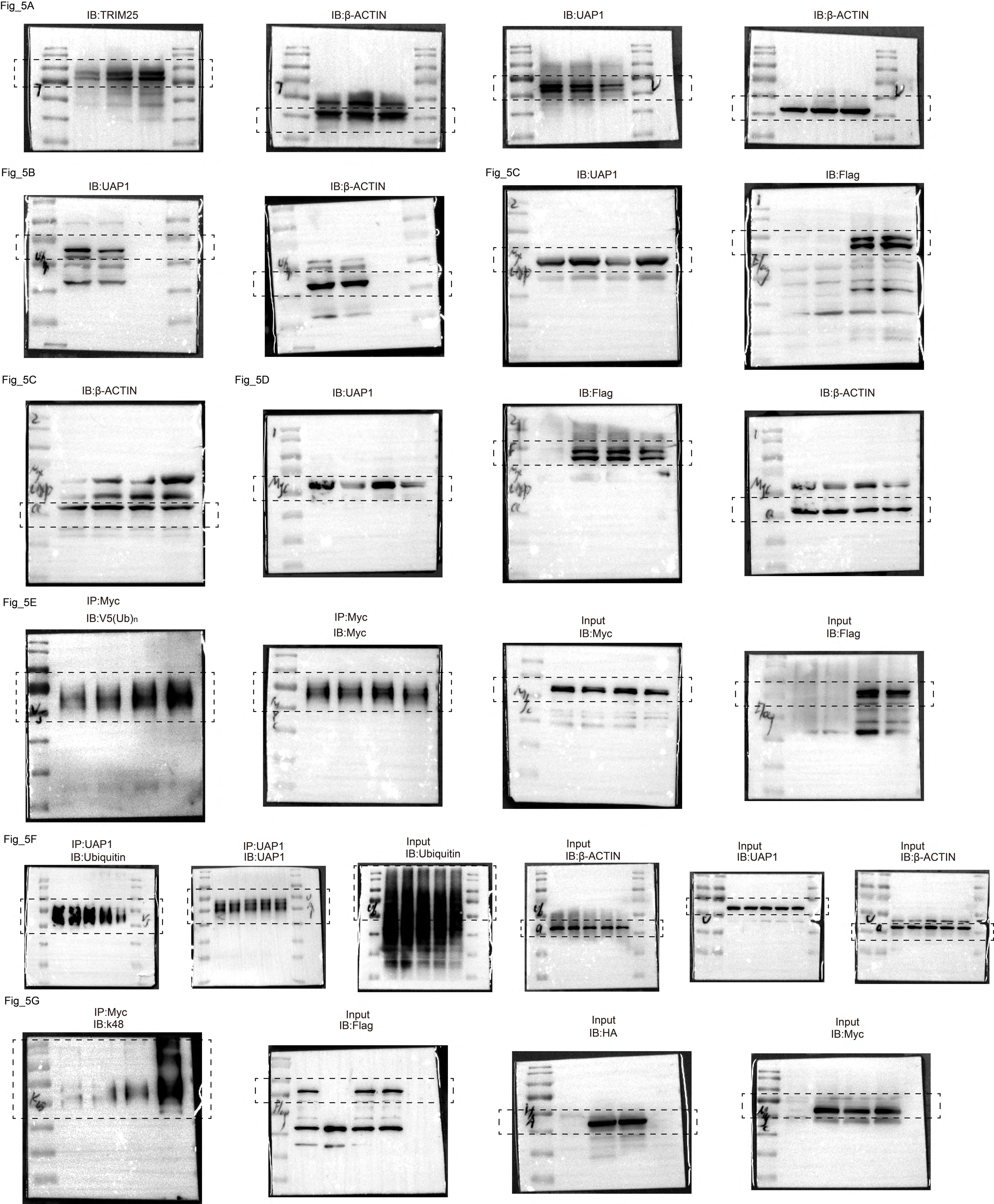
Fig_5A
IB:TRIM25
IB:β-ACTIN
IB:UAP1
IB:β-ACTIN
Fig_5B
IB:UAP1
IB:β-ACTIN
Fig_5C
IB:UAP1
IB:Flag
Fig_5C
IB:β-ACTIN
Fig_5D
IB:UAP1
IB:Flag
IB:β-ACTIN
Fig_5E
IP:Myc
IB:V5(Ub)n
IP:Myc
IB:Myc
Input
IB:Myc
Input
IB:Flag
Fig_5F
IP:UAP1
IB:Ubiquitin
IP:UAP1
IB:UAP1
Input
IB:Ubiquitin
Input
IB:β-ACTIN
Input
IB:UAP1
Input
IB:β-ACTIN
Fig_5G
IP:Myc
IB:k48
Input
IB:Flag
Input
IB:HA
Input
IB:Myc

Fig_6C

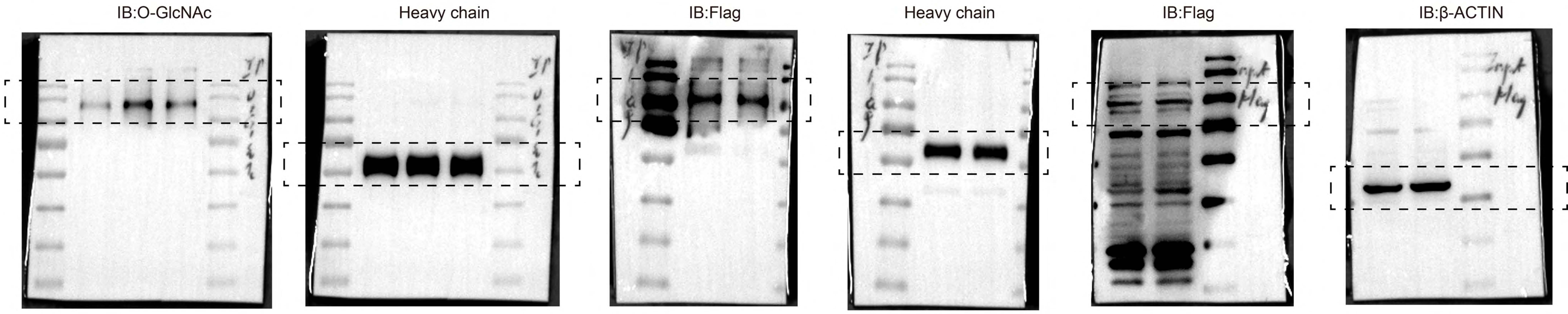

Fig_6D

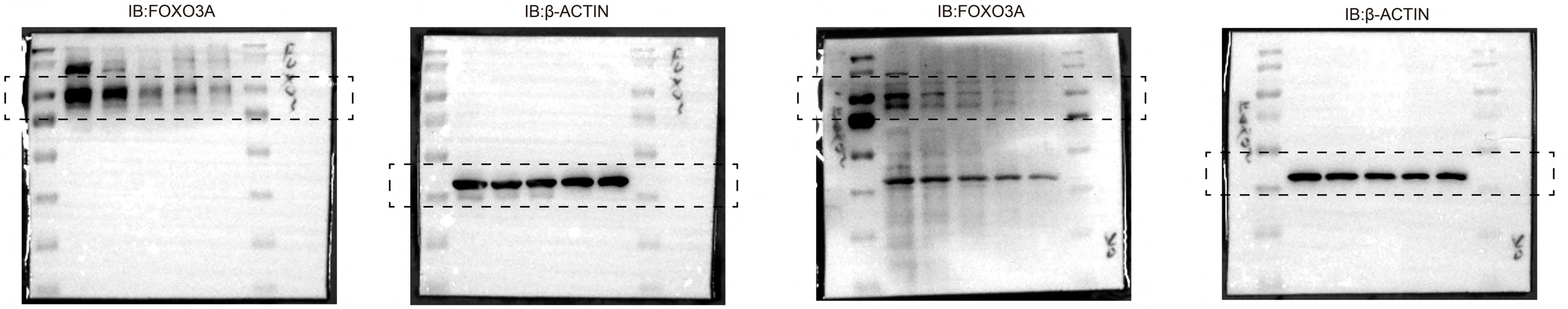

Fig_6E

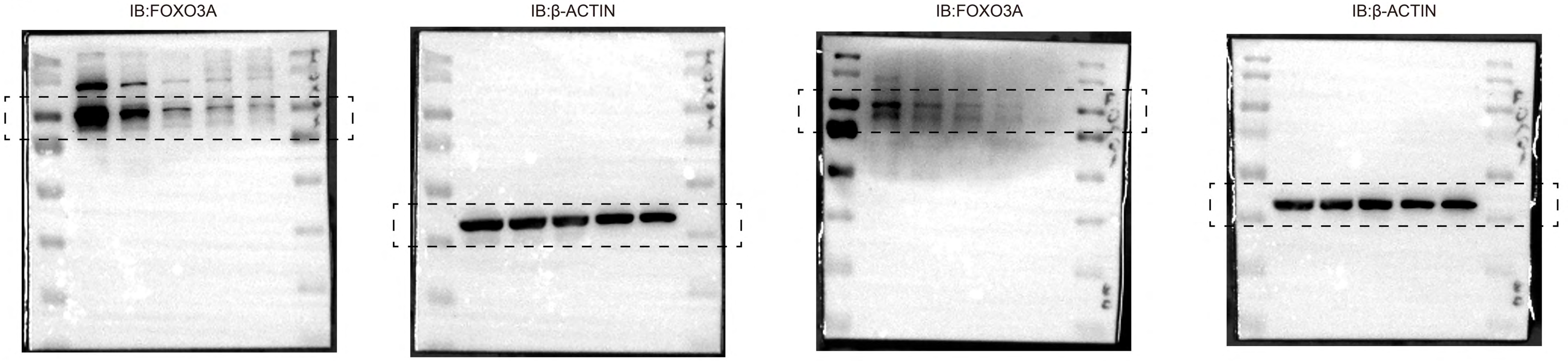

Fig_6H

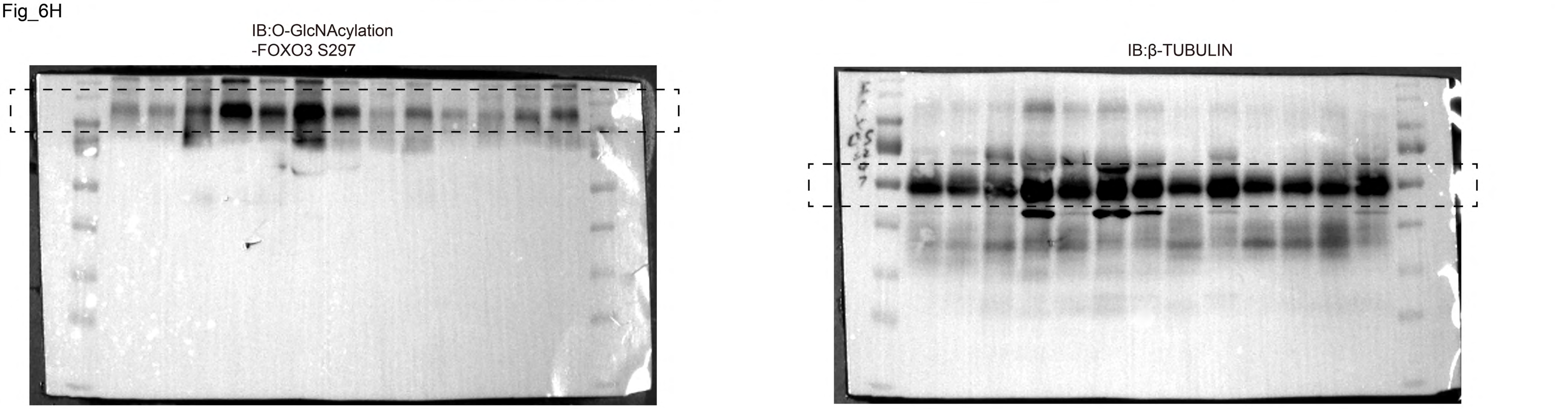

Fig_6I

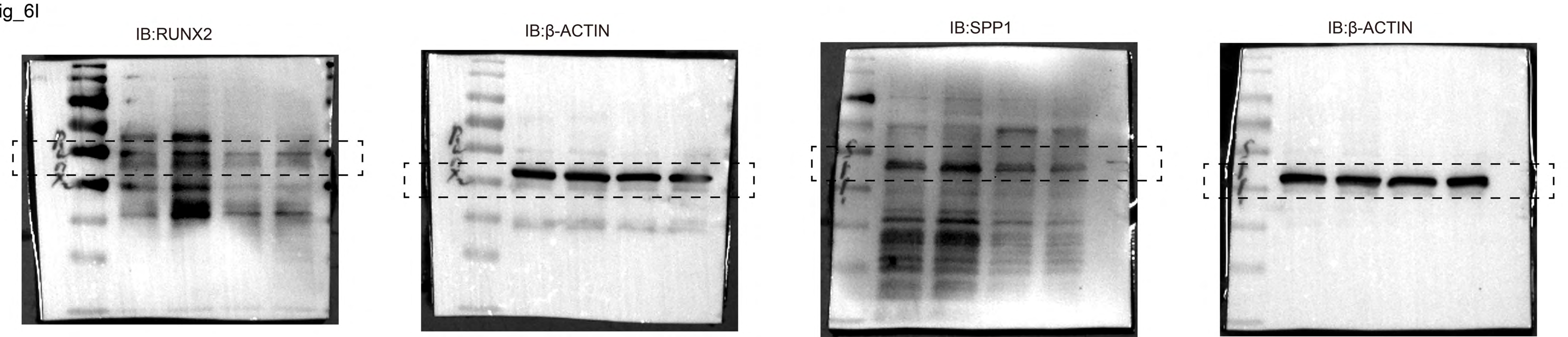

Fig_4C（left）

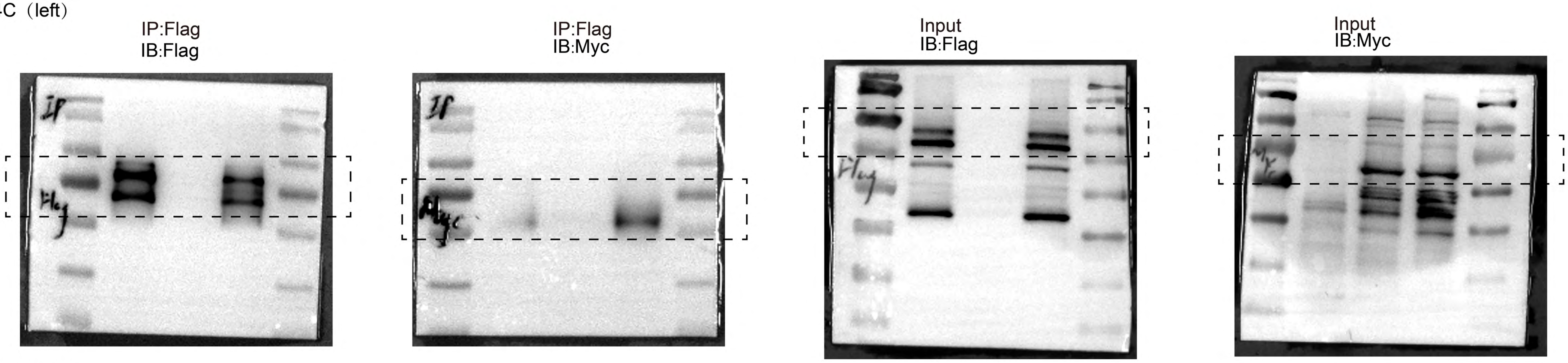

Fig_4C（right）

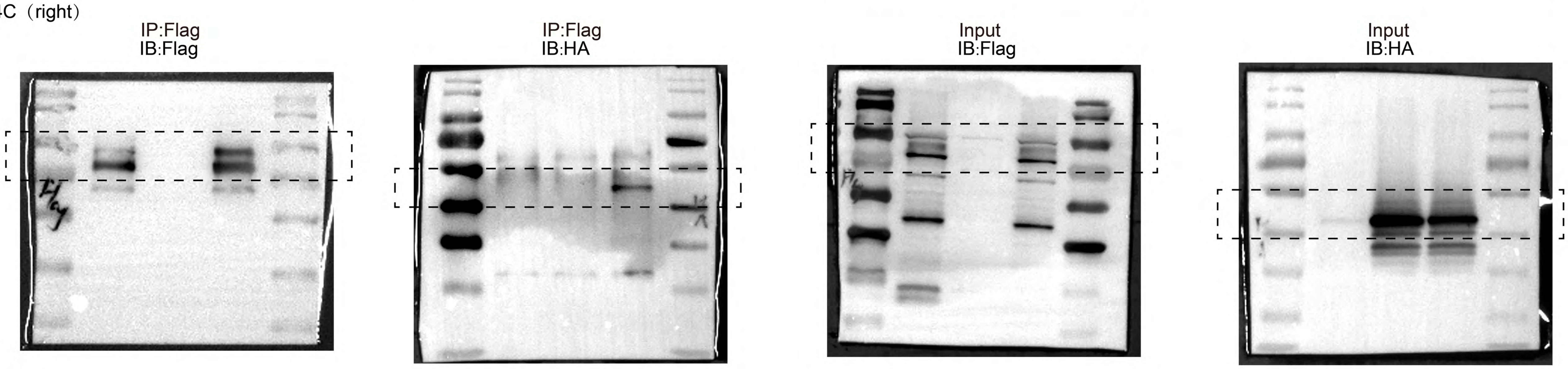

Fig_4H（left）

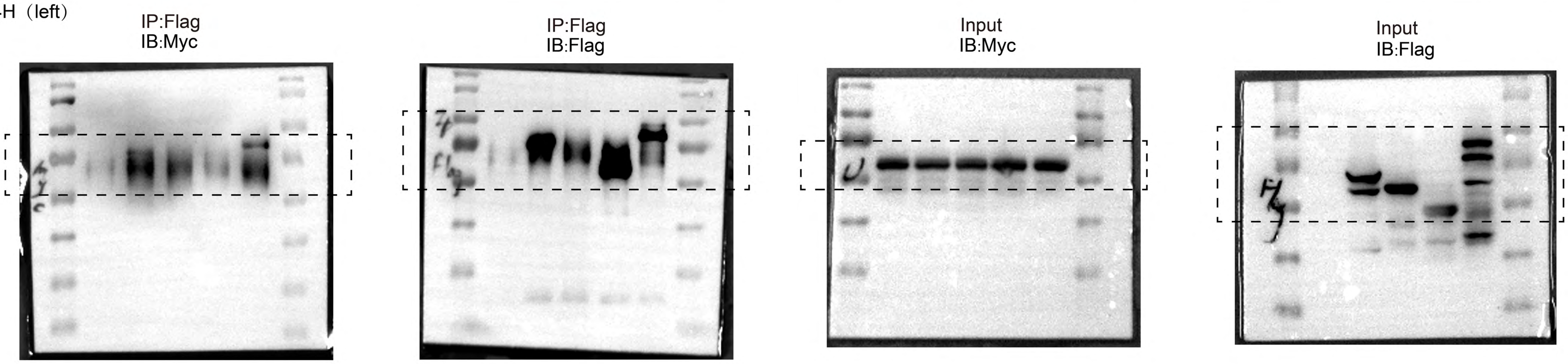

Fig_4H（right）

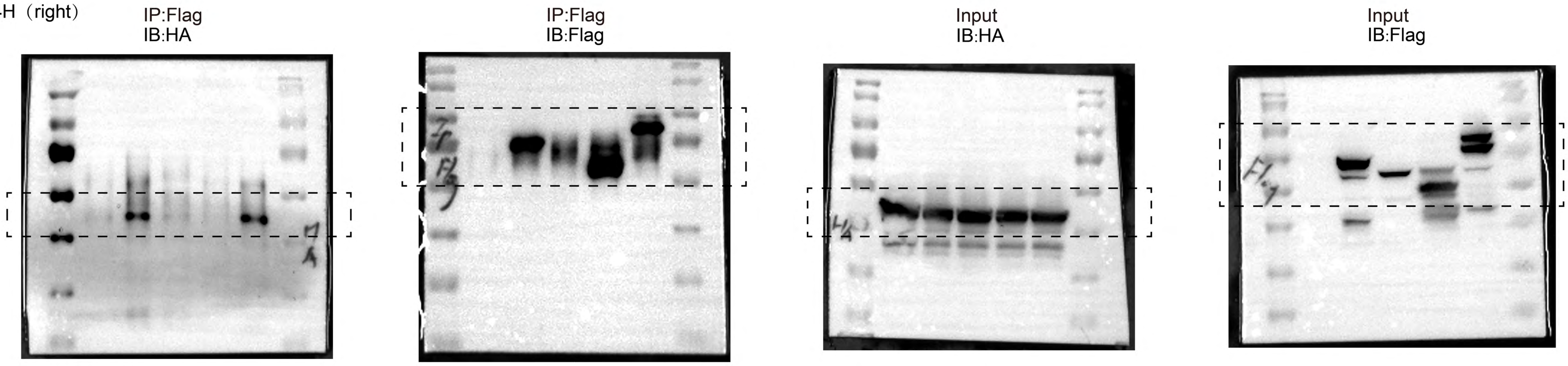

Supplemental Fig_4C

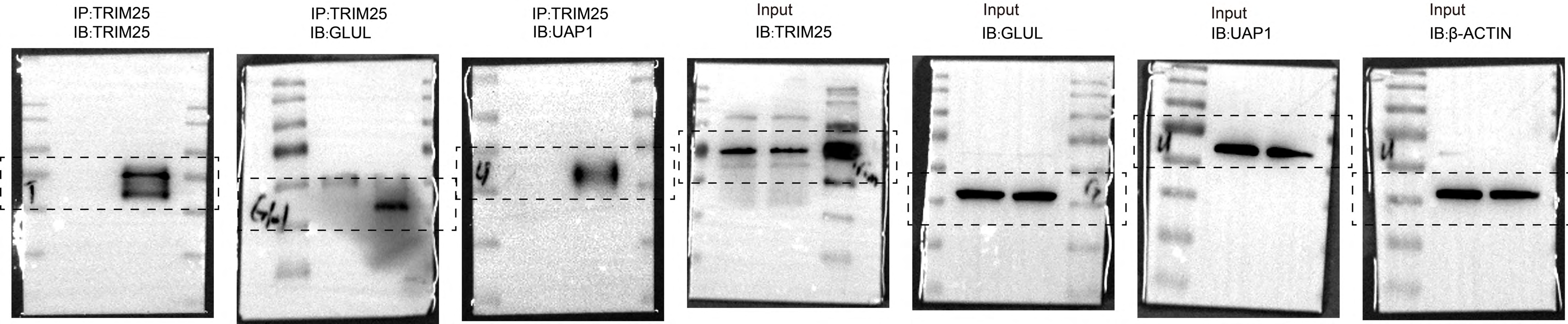

Supplemental Fig_5H

IB:O-GlcNAc

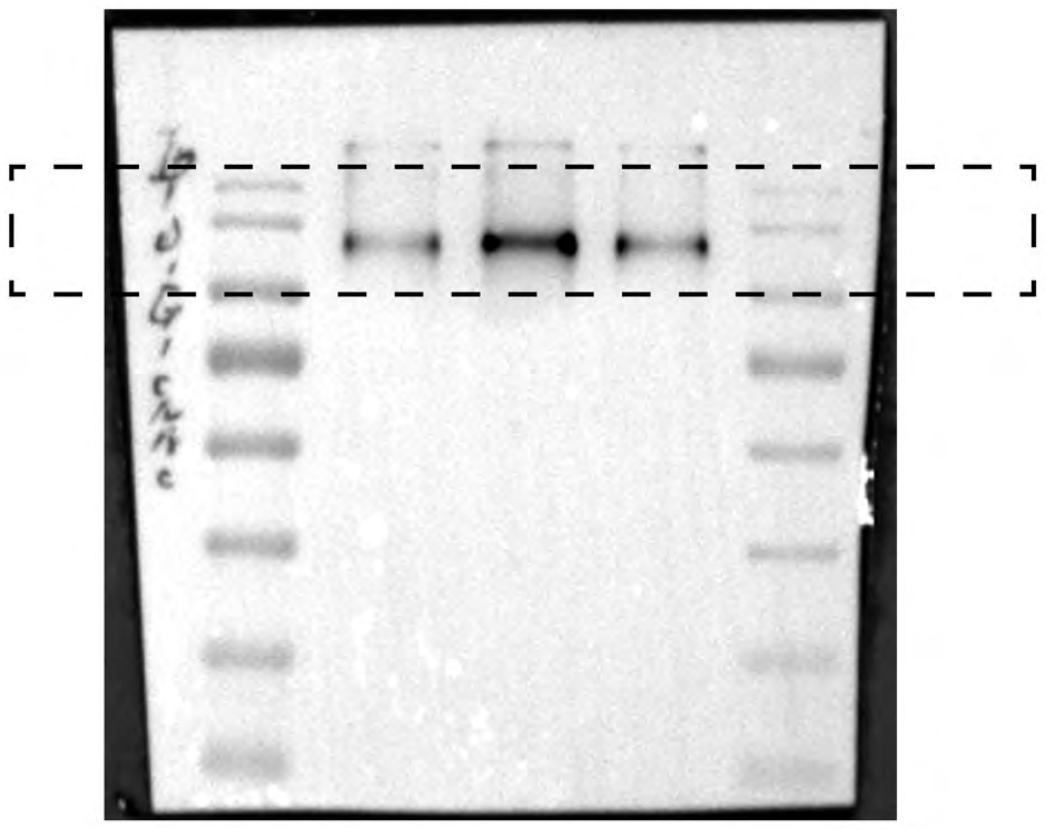

Heavy chain

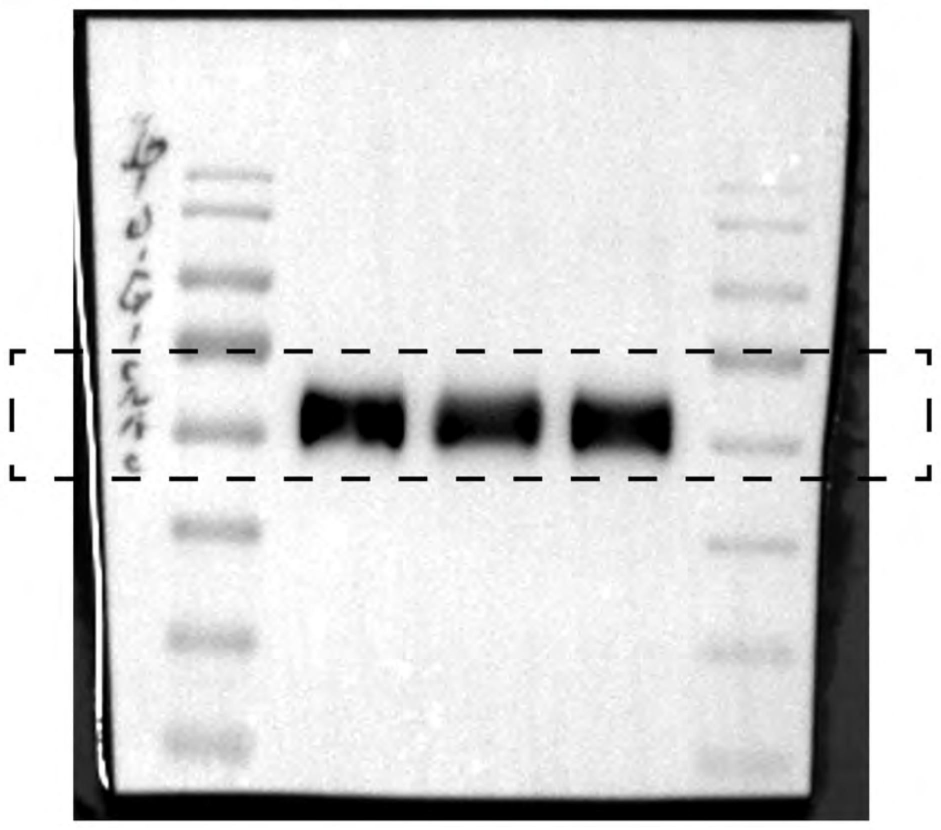

IB:Flag

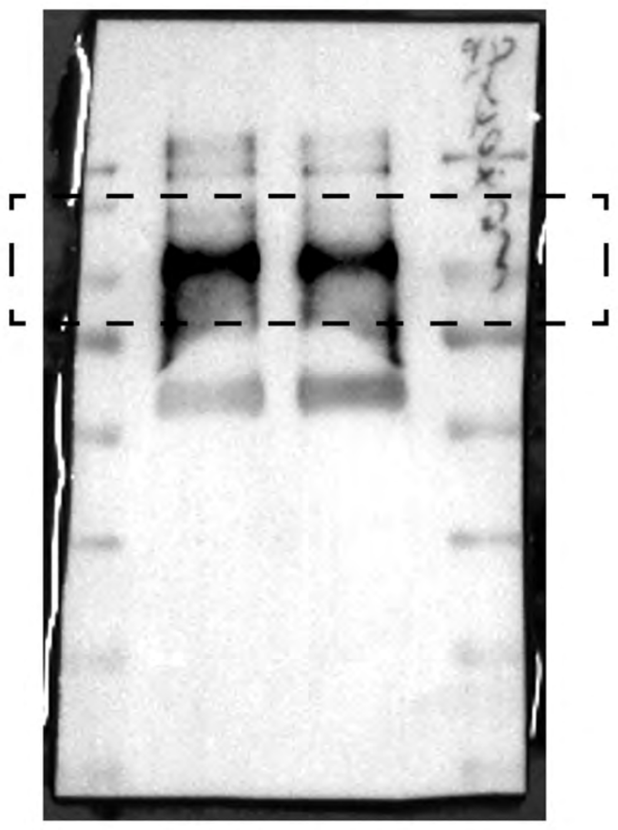

Heavy chain

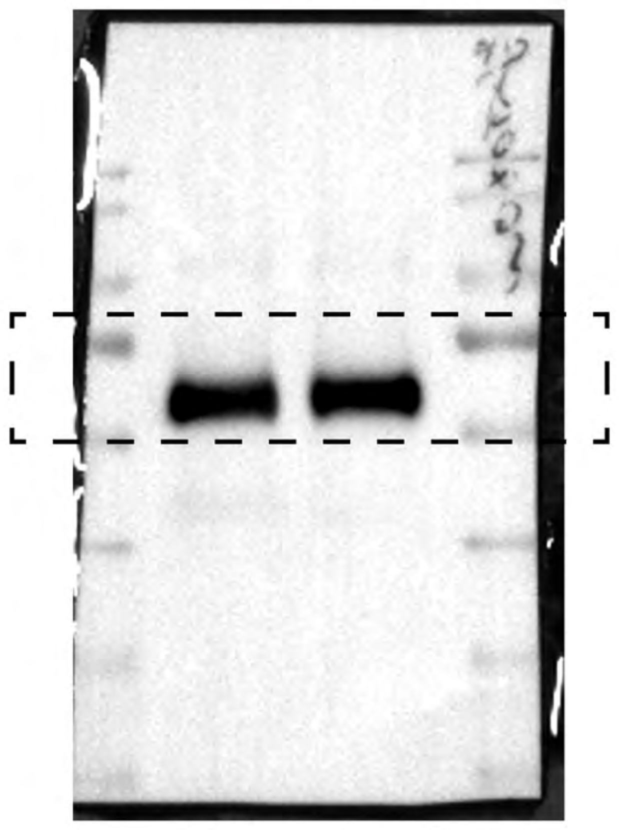

Supplemental Fig_5I

IB:Flag

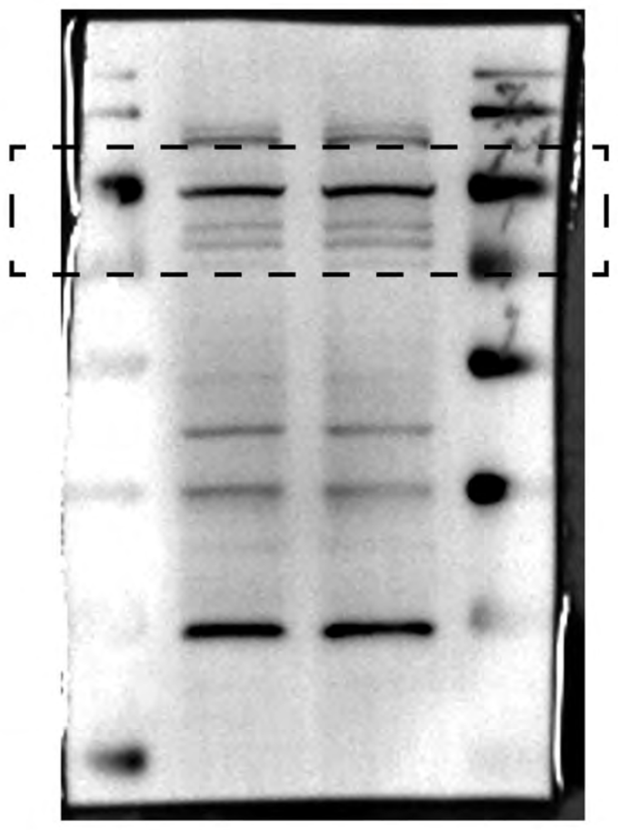

IB:β-ACTIN

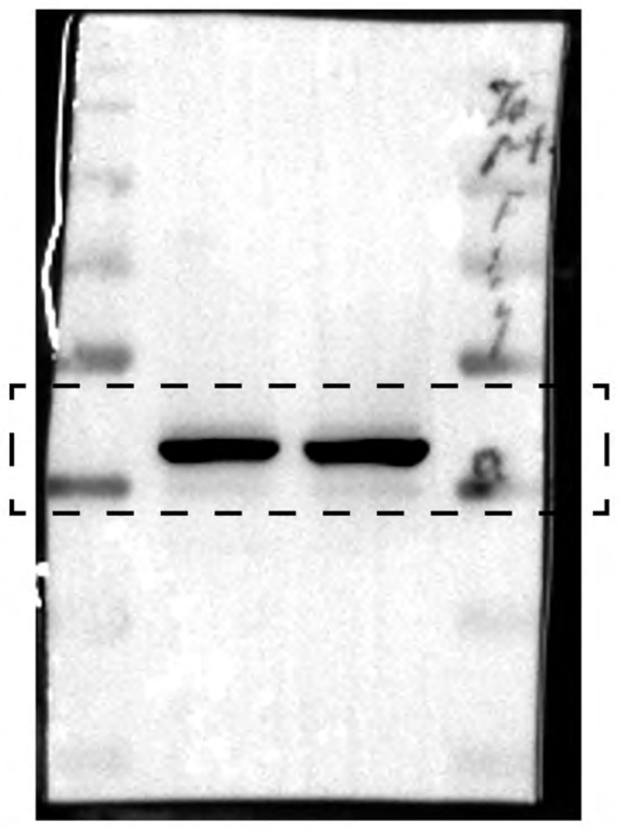

IB:UAP1

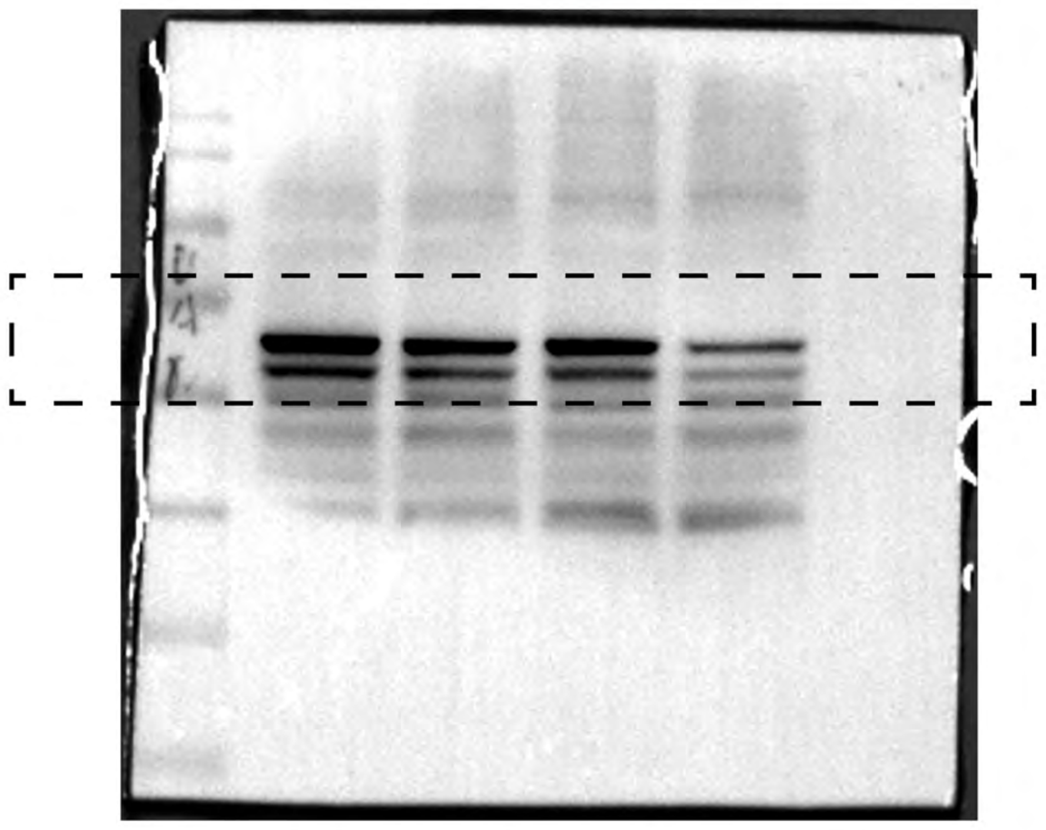

IB:β-ACTIN

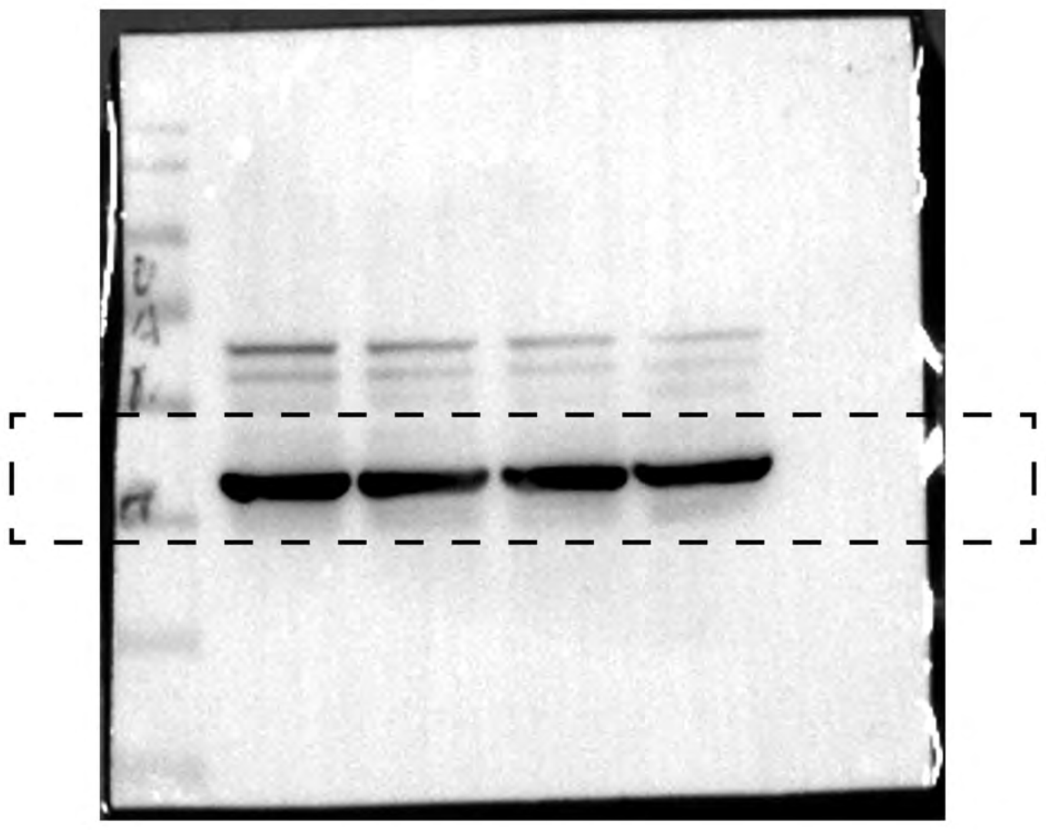

Supplemental Fig_5J

IB:FOXO3A

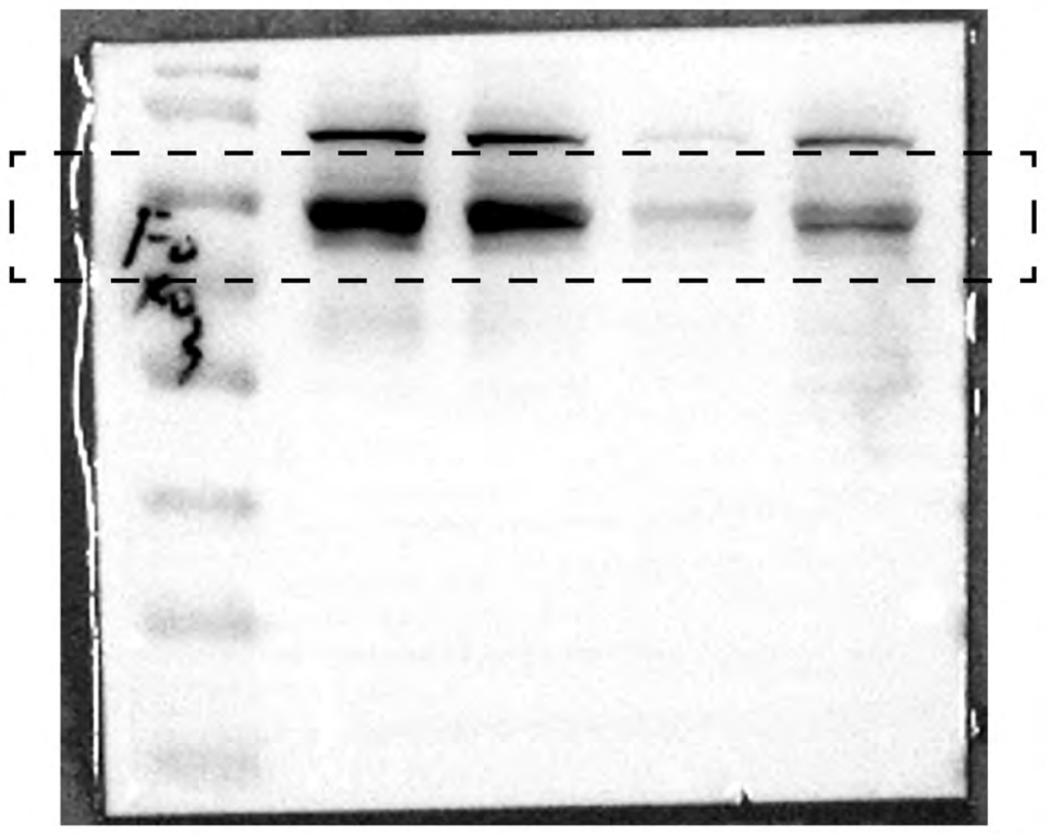

IB:β-ACTIN

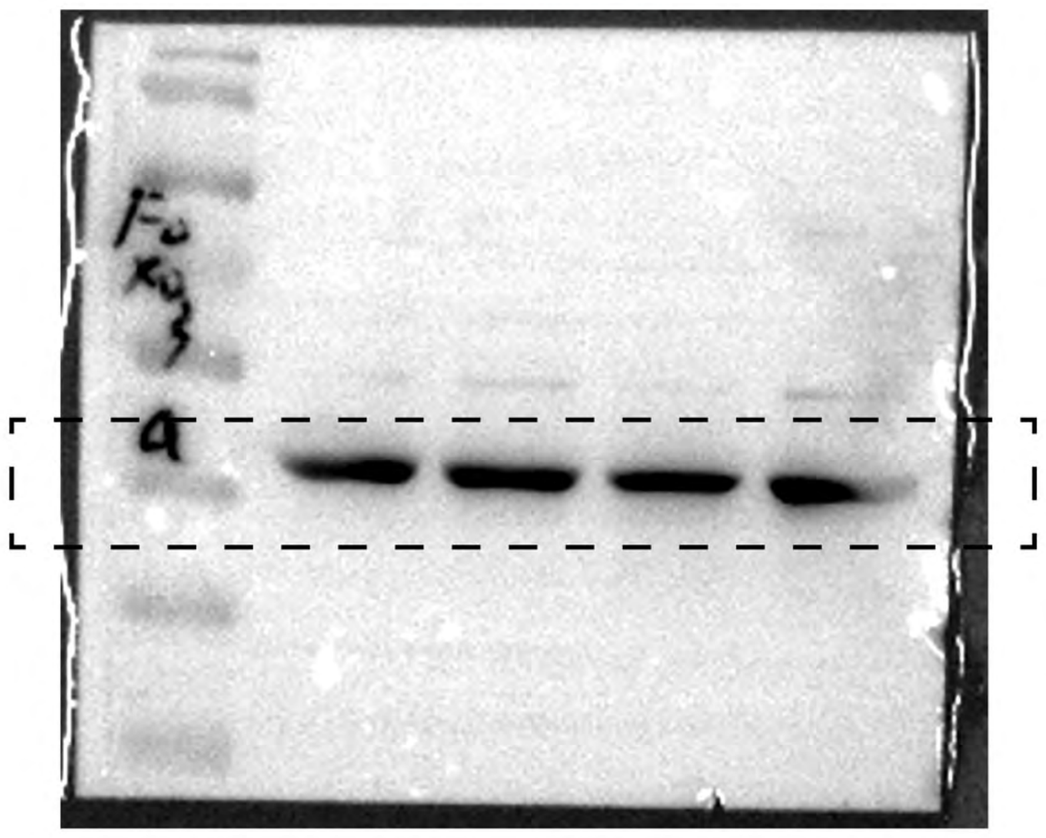

Fig_7K

IB:RUNX2

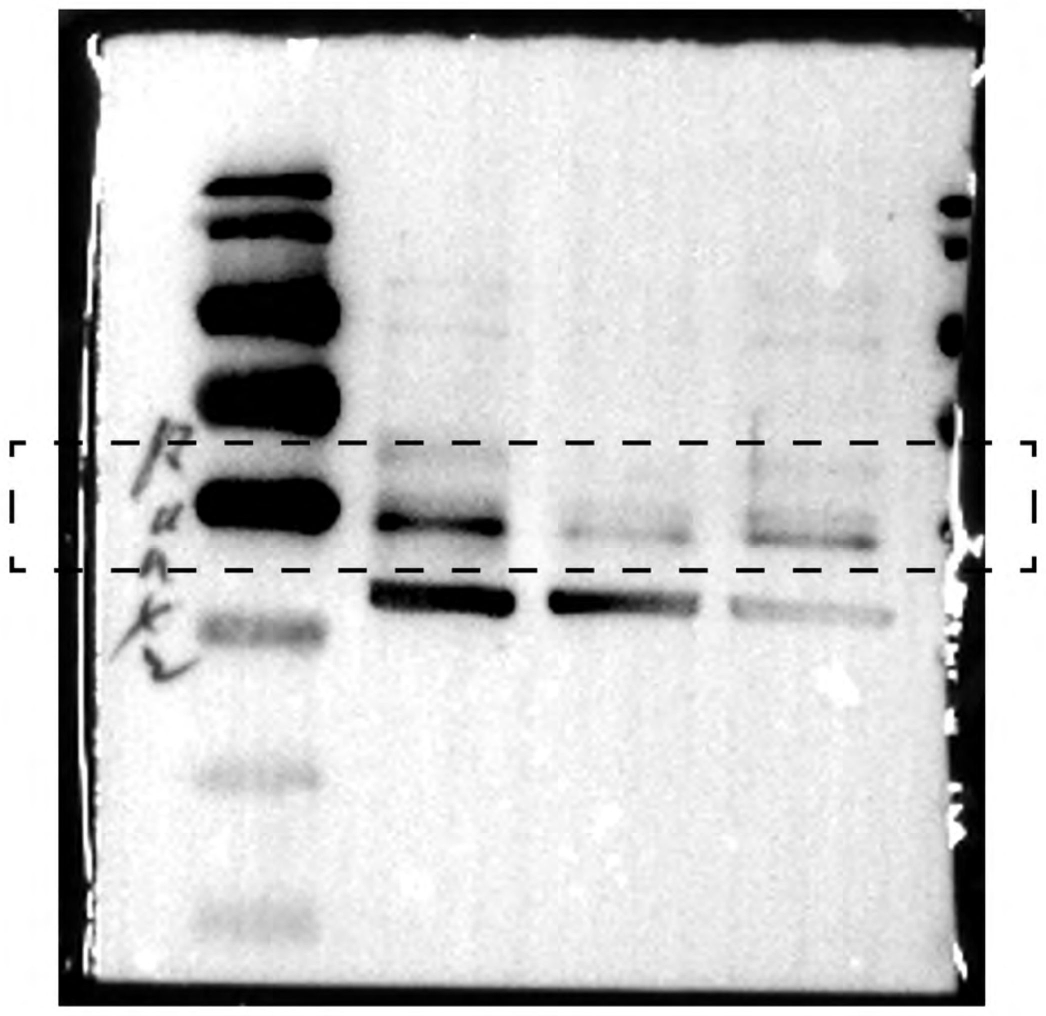

IB:β-ACTIN

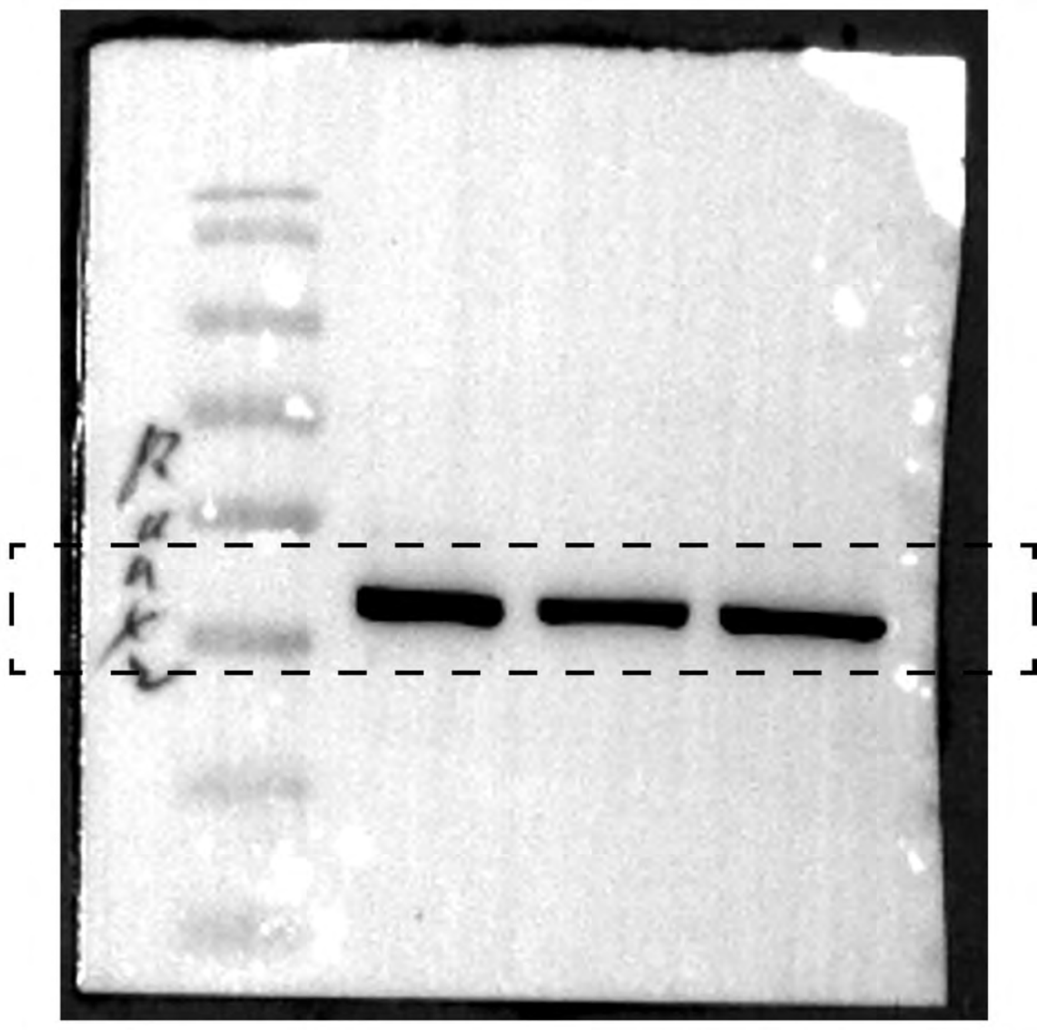

Fig_7K

IB:OSX

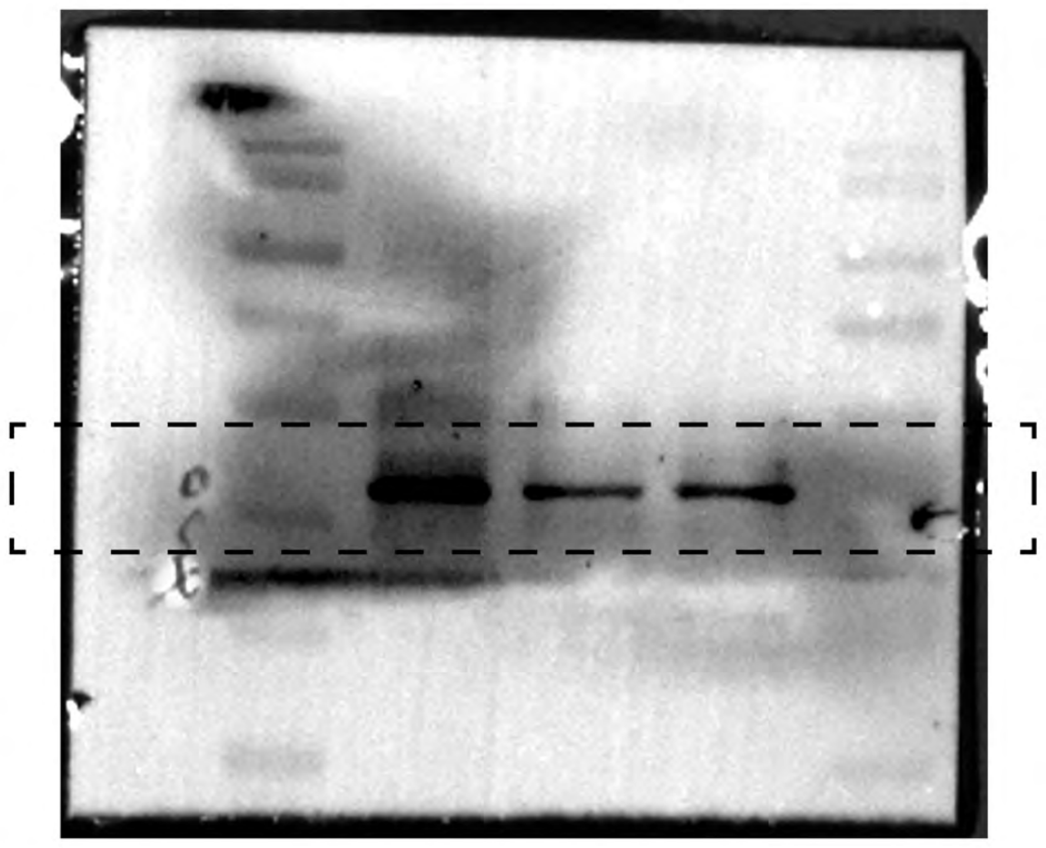

IB:β-ACTIN

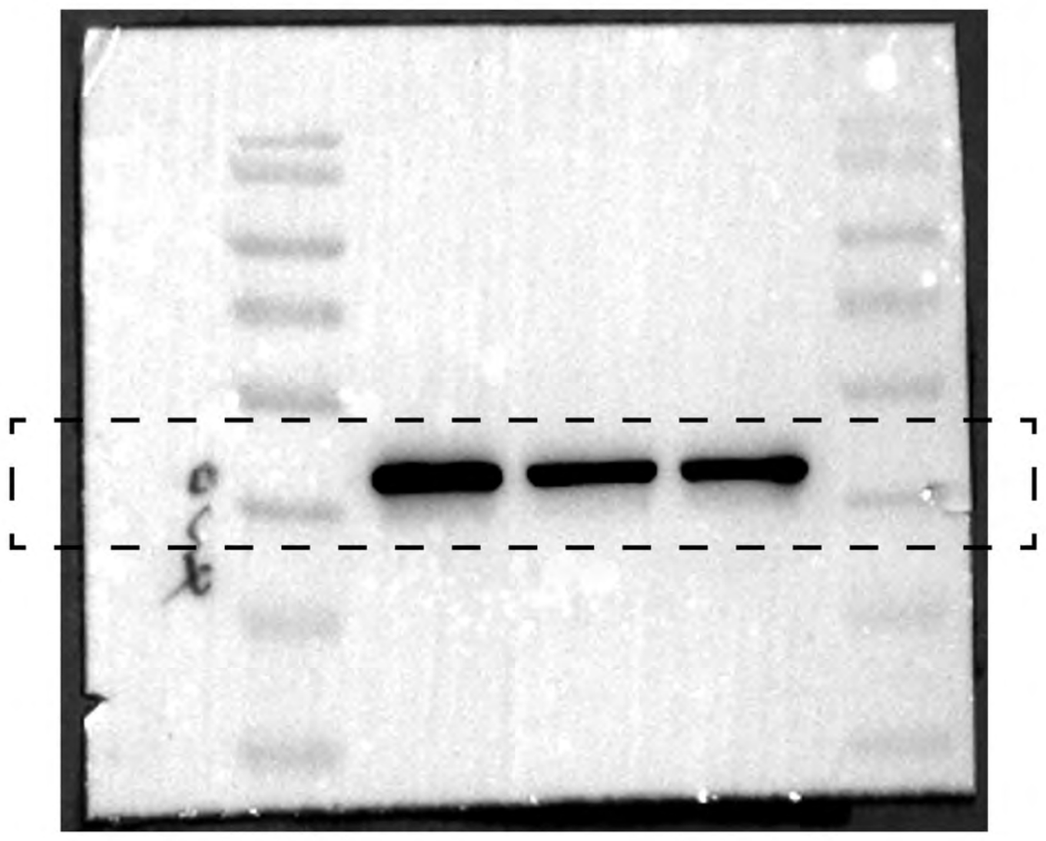

IB:SPP1

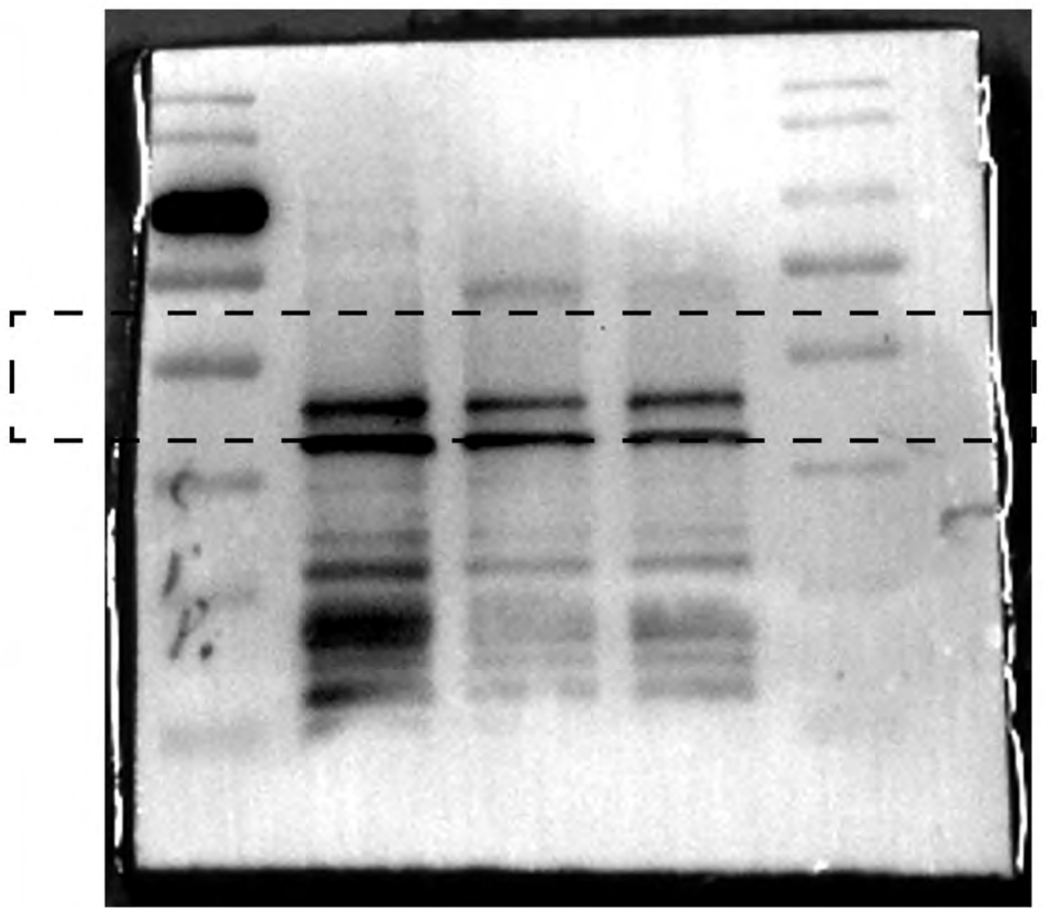

IB:β-ACTIN

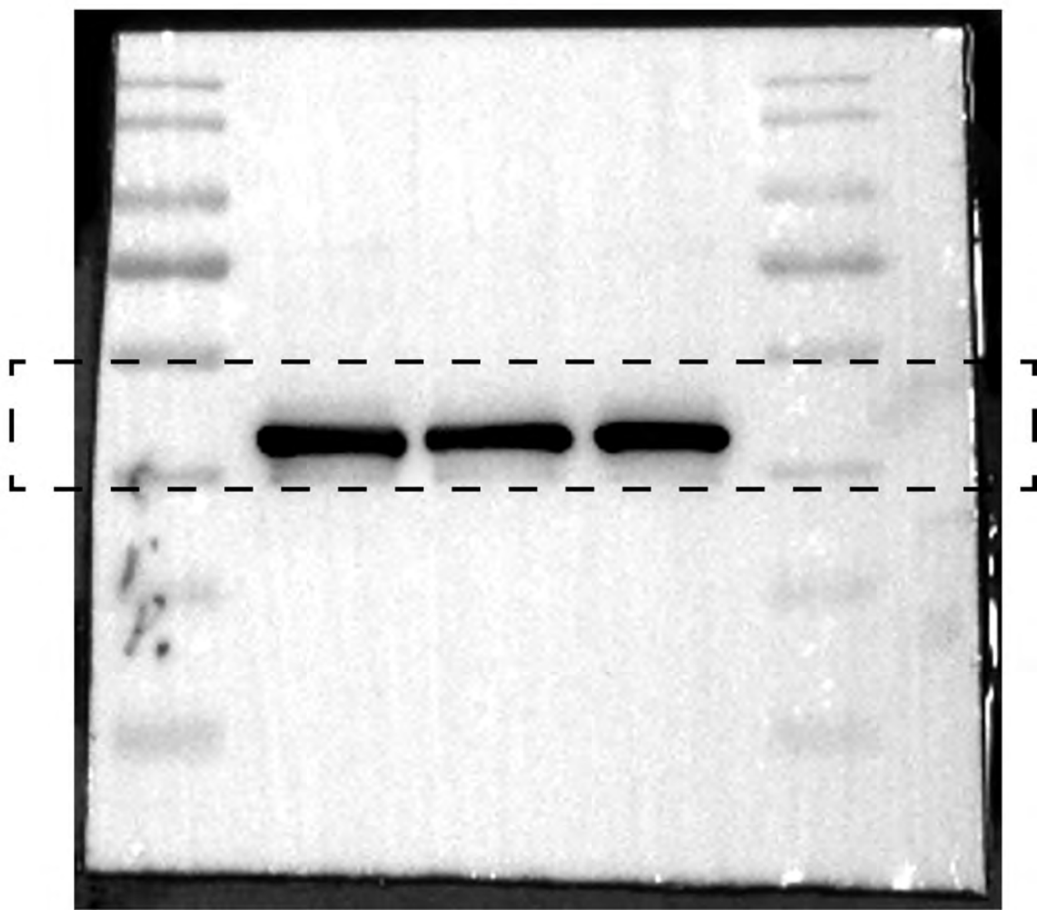

Supplement: Supplementary file 2 — Supplementary original blots [file 41418_2025_1543_MOESM2_ESM.pdf]
